# Stereorandomized Oncocins with Preserved Ribosome Binding and Antibacterial Activity

**DOI:** 10.1021/acs.jmedchem.4c01768

**Published:** 2024-10-24

**Authors:** Bee Ha Gan, Etienne Bonvin, Thierry Paschoud, Hippolyte Personne, Jérémie Reusser, Xingguang Cai, Robert Rauscher, Thilo Köhler, Christian van Delden, Norbert Polacek, Jean-Louis Reymond

**Affiliations:** † Department of Chemistry, Biochemistry and Pharmaceutical Sciences, University of Bern, Freiestrasse 3, 3012 Bern, Switzerland; ‡ Department of Microbiology and Molecular Medicine, University of Geneva, Service of Infectious Diseases, University Hospital of Geneva, 1211 Geneva, Switzerland

## Abstract

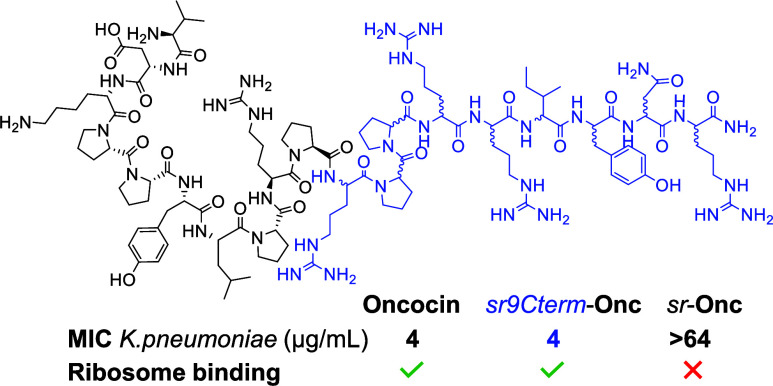

We recently showed that solid-phase peptide synthesis using racemic amino acids yields stereorandomized peptides comprising all possible diastereomers as homogeneous, single-mass products that can be purified by HPLC and that stereorandomization modulates activity, toxicity, and stability of membrane-disruptive cyclic and linear antimicrobial peptides (AMPs) and dendrimers. Here, we tested if stereorandomization might be compatible with target binding peptides with the example of the proline-rich AMP oncocin, which inhibits the bacterial ribosome. Stereorandomization of up to nine *C*-terminal residues preserved ribosome binding and antibacterial effects including activities against drug-resistant bacteria and protected against serum degradation. Surprisingly, fully stereorandomized oncocin was as active as L-oncocin in dilute growth media stimulating peptide uptake, although it did not bind the ribosome, indicative of an alternative mechanism of action. These experiments show that stereorandomization can be compatible with target binding peptides and can help understand their mechanism of action.

## Introduction

Despite being easily accessed by solid-phase peptide synthesis (SPPS),^1^ peptide drugs have long been the problem child of drug discovery due to their poor pharmacokinetics.^2^ Nevertheless, recent successes have turned the interest around in this important modality and encouraged the development of new approaches to modulate peptide properties.^3,4^ Considering that introducing any non-natural building block in a natural peptide sequence may lead to undesirable metabolism and toxicity, one of the preferred modifications is the inversion of stereochemistry at a few specific residues. This minor modification often increases protease stability and, if well chosen, preserves the secondary structure and activity. However, except for the complete inversion of all stereocenters to make the full D-sequence, which is most often stable but entirely inactive, the effect of multiple stereochemical inversions on peptide properties is usually not investigated because the number of possibilities is overwhelming; for instance, there are over one million possible diastereomers for a 20-mer peptide.

Recently, we showed that the vast chemical space of peptide diastereomers can be addressed globally by synthesizing all possible diastereomers simultaneously using racemic building blocks in SPPS.^5^ The resulting stereorandomized (*sr-*) peptides are single-peak single-mass products that can be purified by preparative HPLC-like homochiral peptides. We applied this approach to membrane-disruptive antimicrobial peptides (AMPs) and peptide dendrimers (AMPDs). Stereorandomization abolished the activity of α-helical AMPs but preserved the activity of random coil AMPs such as indolicidin and partially of cyclic peptides such as polymyxin B. In the case of AMPDs, stereorandomization abolished hemolysis but preserved antibacterial effects, implying that their bioactive antibacterial conformation was intrinsically disordered.^6^ In all cases, *sr-*peptides were resistant to serum degradation, showing that the perturbation of homochirality led to resistance to proteases.

The above-mentioned studies of *sr*-peptides were dedicated to membrane-disruptive compounds, leaving open the question of whether stereorandomization might be useful for peptides binding to a specific target. Here, we addressed this question by investigating the proline-rich antimicrobial peptide (PrAMP) oncocin (VDKPPYLPRPRPPRRIYNR), a 19-residue PrAMP modified from a natural peptide identified in the milkweed bug *Oncopeltus fasciatus* and active against various Gram-negative bacteria.^7−13^ Similar to other PrAMPs, oncocin enters cells in a process facilitated by the peptide transporter SbmA and acts by a nonlytic mechanism involving intracellular targets.^14,15^ Identified interactions include the promiscuous substrate binding site of the chaperone DnaK (also known as heat shock protein HSP70),^16−20^ and inhibition of translation by binding to the exit tunnel of the bacterial ribosome.^21−26^ Ribosome inhibition is recognized as the main site of action of PrAMPs^27^ and has been characterized structurally for oncocin^22−24^ and for the glycosylated PrAMP drosocin.^28,29^

**Figure 1:**
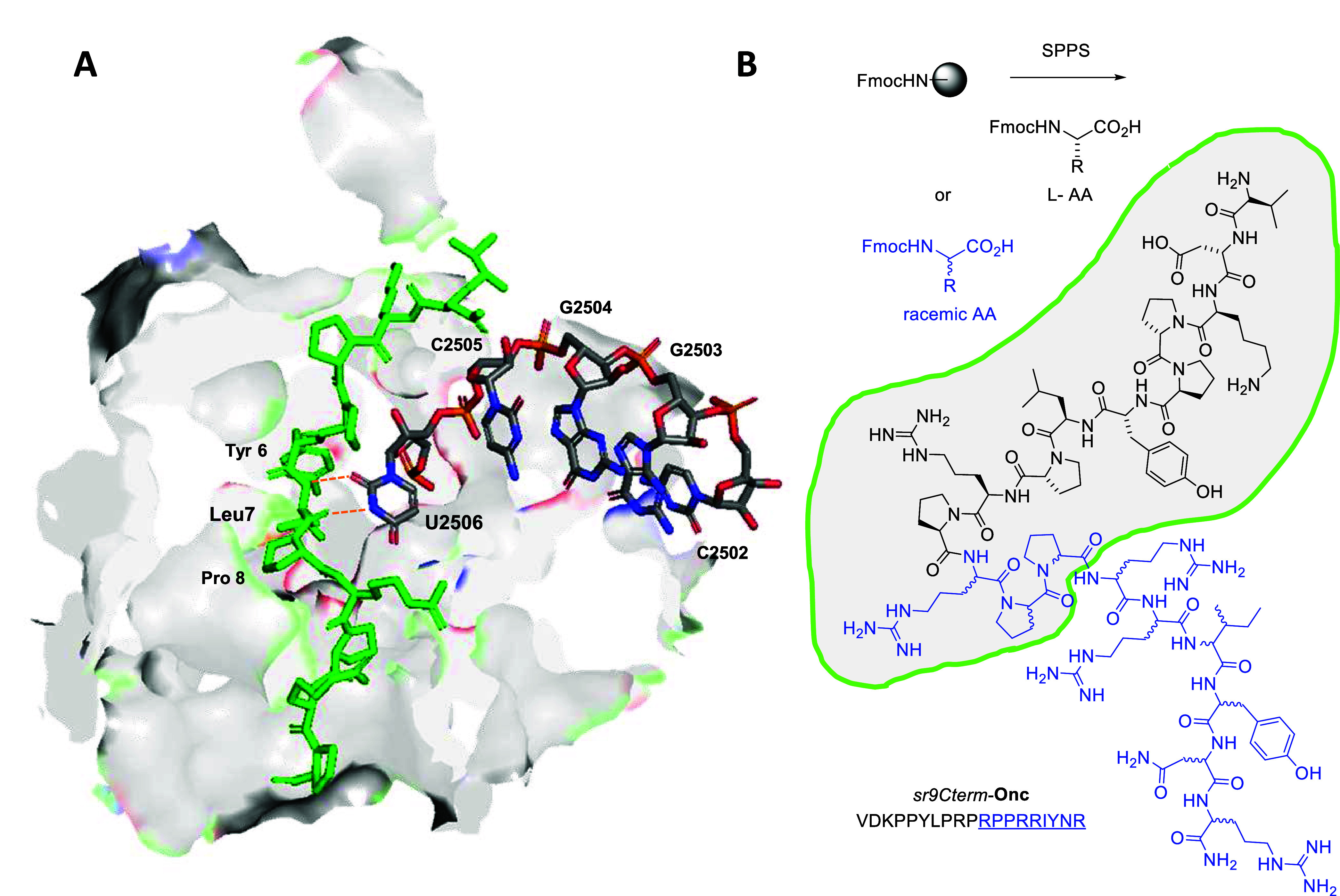
Partial stereorandomization of oncocin preserves ribosome binding. (A) Structure of ribosome-bound oncocin analog **Onc112** from PDB 4ZER. The 23S rRNA U2506 interacting with **Onc112** is highlighted. Only residues 1–13 are visible in this structure. (B) SPPS and the sequence of partially stereorandomized *sr9Cterm*-**Onc** using L- and racemic amino acid building blocks. Stereorandomized positions are highlighted in blue. Residues visible in the ribosome-bound structure are circled in green.

Here, we focus on the interaction of oncocin (L-**Onc**) with the ribosome, which is more specific than the interaction with DnaK and the major target of this peptide,^30,31^ as evidenced, for example, by the fact that DnaK null mutants are still susceptible to oncocin.^21^ Ribosome binding at the exit tunnel involves residues 1–14 from the *N*-terminus of oncocin (Figure 1). The *C*-terminal pentapeptide RIYNR is not essential and can be removed,^24^ which has also led to the report of two more stable analogs in which arginines 15 and 19 have been exchanged for ornithines (**Onc72**) or d-arginines (**Onc112**).^9,32,33^ To test the compatibility of ribosome binding with stereorandomization, we synthesized a series of partially stereorandomized oncocins. As discussed below, antimicrobial activity and ribosome targeting were indeed preserved in several analogs such as *sr9Cterm*-**Onc** containing nine stereorandomized *C*-terminal residues (Figure 1b). Furthermore, we found that the fully stereorandomized *sr*-**Onc**, the d-enantiomer D-**Onc**, and several nonribosome binding analogs had a similar antibacterial activity as L-**Onc** in dilute growth media used to stimulate peptide uptake, indicative of an alternative mechanism of action.

## Results

### Design and Synthesis of Stereorandomized Oncocin Analogs

Considering that the last five residues at the *C-*terminus were known to be nonessential to oncocin activity,^24^ we prepared a first analog by stereorandomizing only these five *C*-terminal residues (*sr5Cterm***-Onc**), which would be expected to retain activity. We then gradually extended stereorandomization up to 12 residues from the *C*-terminus (*sr6Cterm***-Onc** → *sr12Cterm***-Onc**), intruding on the part of the *N*-terminal 14-residue sequence known to directly interact with the ribosome, anticipating an activity decrease. We also directly introduced stereorandomization into the essential ribosome binding *N*-terminal part to test if this modification altered the activity, targeting either the proline dipeptide at positions 4 and 5 (*srP45-***Onc**), or the *N*-terminal VDK tripeptide (*sr3Nterm*-**Onc**), optionally combined with stereorandomization of the nonessential 5 *C*-terminal residues (*sr3N5Cterm*-**Onc**). Finally, we completely stereorandomized the essential 14-residue *N*-terminus (*sr14Nterm*-**Onc**) and the entire oncocin sequence (*sr*-**Onc**).

For comparison, we prepared diastereomeric oncocins by inverting the stereochemistry of selected residues to D-chirality, starting again with the nonessential *C*-terminal pentapeptide (D5Cterm-**Onc**), the *C-*terminal octapeptide (D8Cterm-**Onc**), and nonapeptide (D9Cterm-**Onc**). We then flipped single residues to D- in the essential 14 *N*-terminal sequences to see if small stereochemical changes in that region affected the activity (DP12-**Onc** → DP4-**Onc**), including the central Tyr6-Leu7 dipeptide, known to be essential for ribosome binding. Finally, we prepared the full d-enantiomer of oncocin (D-**Onc**), expected to be inactive, as well as L-oncocin (L-**Onc**) and its optimized analogs **Onc72** and **Onc112** as positive controls.^9,21,22,24,34^ All of the above peptides were synthesized by high-temperature SPPS on Rink-amide resin using Fmoc-protected L- and D-amino acids, either pure or as a 1:1 mixture at stereorandomized positions and obtained as homogeneous products after preparative HPLC (Table 1).

**Table 1:**
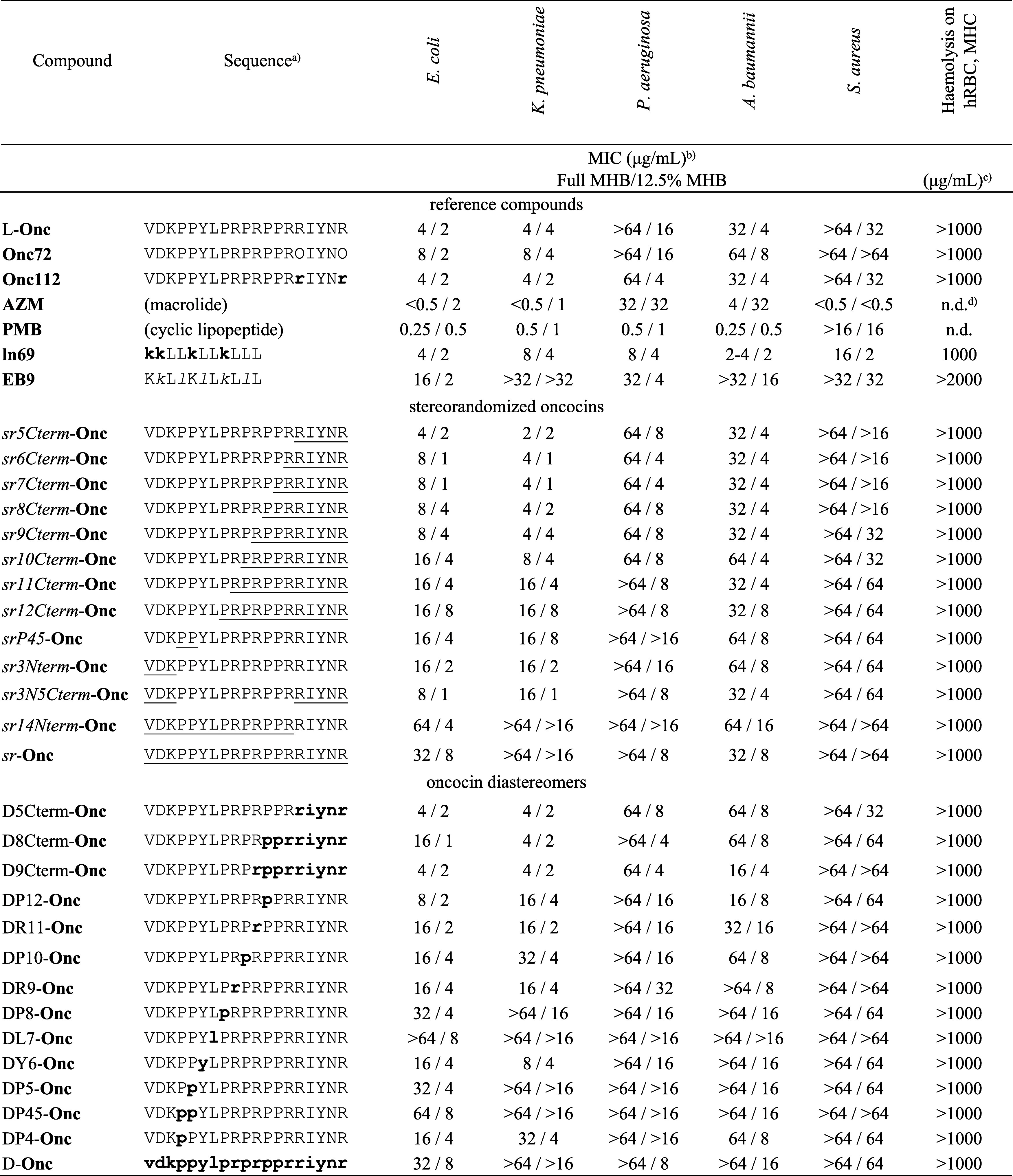
Synthesis and Activity of Stereorandomized and Diastereomeric Oncocins

a One-letter codes for amino acids with O = l-ornithine, upper-case = L-, upper-case underlined = stereorandomized, lower-case boldface = d-, lower-case italics = peptoid. All C-termini are carboxamides.

b Minimum inhibitory concentration (MIC, μg/mL) was determined on *P. aeruginosa* PAO1, *A. baumannii* ATCC19606, *E. coli* W3110, *K. pneumoniae* NCTC 418, and *S. aureus* COL (MRSA) in full and 12.5% Müller–Hinton medium after incubation for 16–20 h at 37 °C.

c Minimum hemolytic concentration (MHC) measured on 2.5% of human red blood cells (final concentration) in 10 mM phosphate, 150 mM NaCl, pH 7.4, 25 °C, 4 h.

d n.d. stands for “not determined”.

### Stereorandomization Affects Antibacterial Activities in Full But Not in Dilute Medium

We tested antibacterial effects against the Gram-negative bacteria *Escherichia coli*, *Klebsiella pneumoniae*, *Pseudomonas aeruginosa*, and *Acinetobacter baumannii* and the Gram-positive methicillin-resistant *Staphylococcus aureus* (MRSA). Minimal inhibitory concentrations (MICs) were determined in microdilution assays in the Muller–Hinton broth (MHB) medium as well as in diluted (12.5%) MHB. While bacterial growth was similar in both conditions (Figure S1), we used dilute MHB because it was reported to increase the activity of PrAMPs such as oncocin,^9,35,36^ an effect attributed to the activation of peptide uptake mechanisms such as the SbmA transporter leading to increased peptide uptake.^36^ As controls for antibacterial assays, we used azithromycin (**AZM**), a ribosome-targeting macrolide antibiotic,^37−39^ polymyxin B (**PMB**), which targets lipid A in the membrane,^40,41^ the linear mixed-chirality peptide **ln69** as a strong membrane-disruptive peptide,^42,43^ and its analog **EB9** containing alternating peptide and peptoid building blocks,^44^ a nonmembrane-disruptive compound acting by aggregating intracellular contents similar to other peptoids.^45^

In full MHB, the positive controls L-**Onc**, **Onc72**, and **Onc112** showed good activity against *E. coli* and *K. pneumoniae* and poorly or not at all active against *A. baumannii*, *P. aeruginosa*, and MRSA. Their activity was enhanced by 2–8 fold in dilute MHB, in agreement with published data.^33,36,46^ A similar activity increase in dilute medium also occurred with peptide **ln69** and peptoid **EB9** as previously reported.^43,44^ Note that bacterial growth was not affected by the dilute medium and the activity of **AZM** and **PMB** was weaker in dilute medium, consistent with the reported increase in peptide uptake in dilute medium increasing only peptide activities.^36^

In full MHB, our oncocin analogs also only showed significant effects against *E. coli* and *K. pneumoniae*, and their activity was strongly influenced by stereochemistry (Table 1; the first MIC value for full media). By comparison to L-**Onc**, the activity was preserved not only with *sr5Cterm*-**Onc**, as anticipated since the 5 *C*-terminal residues are nonessential, but also in subsequent sequences with *sr*-alternation up to *sr9Cterm-***Onc** with four stereorandomized positions in the essential 14-residue *N*-terminal region. Even full D-residues were tolerated in these 9 *C*-terminal positions, as indicated by the preserved activity of D9Cterm-**Onc**. These activities were controlled by the SbmA transporter, as evidenced by the reduced activity of L-**Onc**, **Onc112**, and *sr9Cterm*-**Onc** on an *E. coli* SbmA deletion mutant compared to WT, in line with previous reports on L-**Onc** and **Onc112** (Table S1, left columns).^12,47^ On the other hand, the fully stereorandomized sequence *sr*-**Onc** and the d-enantiomer D-**Onc** were almost entirely inactive in full MHB. Similarly, sequences with stereochemical perturbations in the essential *N*-terminal 14-residue stretch showed reduced activities. Activities were strongly reduced for *sr10Cterm-***Onc** and subsequent stereorandomized sequences, and for DR11-**Onc** and subsequent D-residue-containing sequences. The effect was particularly strong whenever the section around the leucine residue at position 7 was either d-enantiomeric or stereorandomized.

In dilute MHB by contrast, all stereorandomized and diastereomeric oncocin analogs synthesized showed good activity against *E. coli* and to a lesser extent against the other three Gram-negative strains tested, indicating that residue stereochemistry had little influence on activity under these conditions (Table 1; the second MIC value for dilute media). These activities were preserved in the *E. coli* SbmA deletion mutant compared to WT, indicating that they did not depend on the SbmA transporter (Table S1, right columns). Taken together, these data showed that partial stereorandomization of the *C-*terminal region of oncocin was compatible with antimicrobial activity in full MHB, while alterations in the *N*-terminal region led to inactive analogs under these conditions. On the other hand, the dilute MHB conditions allowed almost all oncocin analogs investigated to be quite active.

### Ribosome Binding Correlates with Activities in Full Medium

As shown by structural and biochemical studies, L-oncocin binds to the bacterial ribosome at the exit tunnel of the growing peptide chain, which inhibits translation.^23,24^ Ribosome binding involves the 14 residues at the *N*-terminus, 13 of which are visible in the structure of the ribosome–oncocin complex (Figure 1). We therefore expected that our partially stereorandomized or d-enantiomeric analogs with preserved L-chirality in residues 1–14, which were almost as active as L-**Onc** in full MHB, should bind the ribosome. On the other hand, oncocin analogs with stereorandomized or enantiomeric positions among residues 1–14, which were inactive in full MHB, might have lost the ability to inhibit the ribosome.

To test this hypothesis, we investigated ribosome binding with the partially stereorandomized active analogs *sr5Cterm*-**Onc**, *sr8Cterm*-**Onc**, and *sr9Cterm*-**Onc**, as well as the corresponding active partial D-sequences D5Cterm-**Onc**, D8Cterm-**Onc**, and D9Cterm-**Onc**, to be compared with L-**Onc** as a positive control. As inactive analogs, we considered *sr*-**Onc**, *sr14Nterm*-**Onc**, D-**Onc**, and DL7-**Onc**. To probe ribosome binding, we performed RNA-footprinting experiments with purified bacterial ribosomes,^24^ in which the binding of L-**Onc** protects uracil at position U2506 of the 23S rRNA from modification by *N*-cyclohexyl-*N*′-(β-[*N*-methylmorpholino]ethyl) carbodiimide *p*-toluenesulfonate (CMCT).^48^ This modification is detected after the isolation of rRNA by premature termination of primer extension at the preceding position C2507 during reverse transcription, implying that the gel electrophoresis band disappears at that position if oncocin is bound.

**Figure 2:**
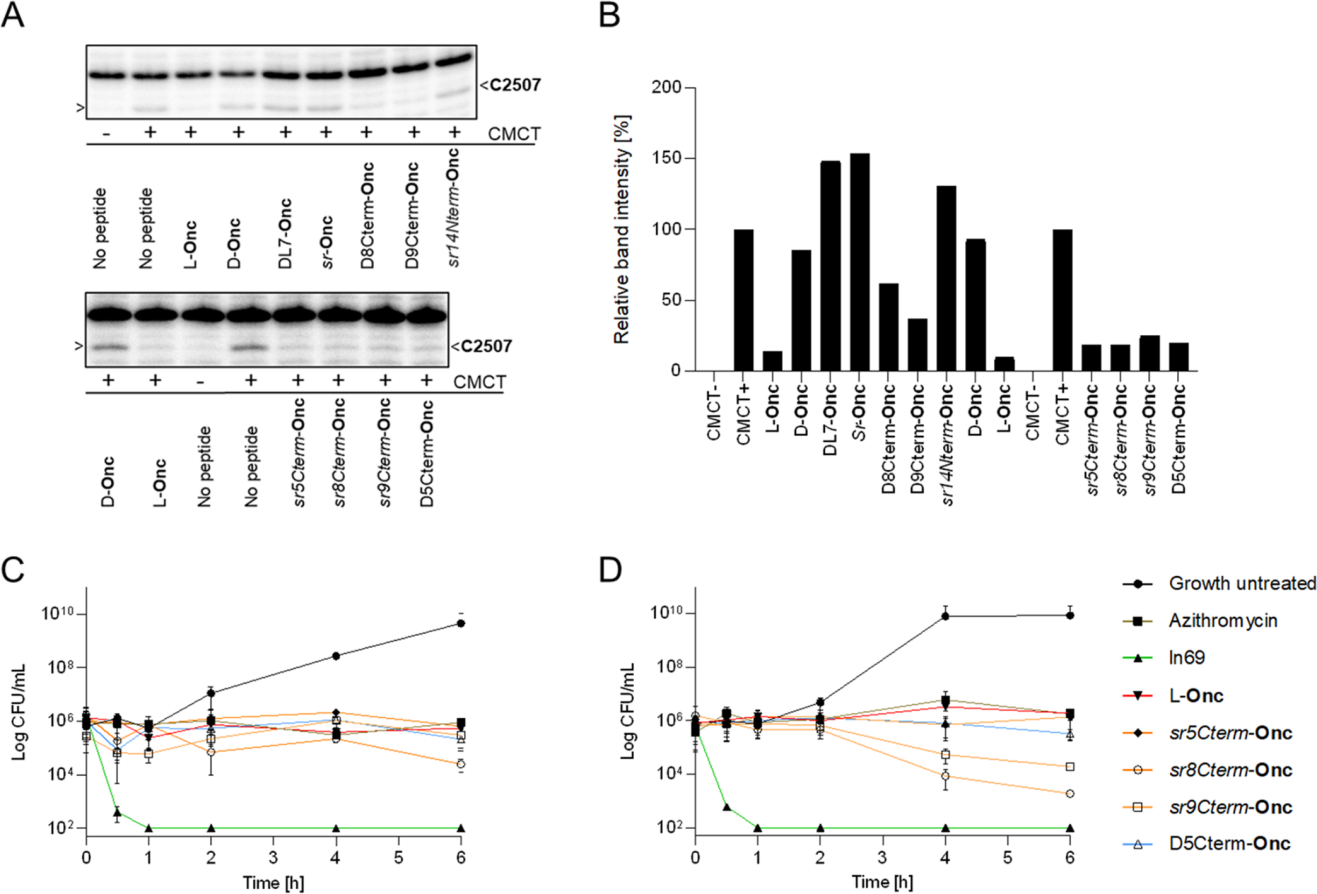
Mechanism of oncocin analogs active in full MHB. (A) *E. coli* ribosome footprinting experiment: the arrows indicate the position C2507 on the sequencing gel of the 23S rRNA. The more intense bands result from the U2506 CMCT modification in the absence of peptide binding. As expected, the CMCT modifies U2506 and produces a visible band at position C2507. In the absence of CMCT, there is no modification of U2506. No bands are observable with L-**Onc**, D8Cterm-**Onc**, D9Cterm-**Onc**, *sr5Cterm*-**Onc**, *sr8Cterm*-**Onc**, *sr9Cterm*-**Onc**, and D5Cterm-**Onc** compared to background, revealing the interaction of the peptide with U2506. On the other hand, bands are visible with D-**Onc**, DL7-**Onc**, and *sr14Nterm*-**Onc** resulting from the absence of peptide binding. The full sequencing gels are available in the Supporting Information: Figures S5 and S6. (B) Bar plot representing the relative band intensity at position C2507 of the sequencing gel (A). The band intensity was measured with the software ImageJ. The relative intensity values were obtained after deduction of the CMCT – intensity and normalization to the CMCT + intensity. (C, D) Killing profile of the active compounds on bacteria, at 8× their MIC value (C) against *E. coli* and (D) against *K. pneumoniae*. The assay was performed twice in triplicate in full MHB at pH 7.4. The data represent the mean ± SD, *n* = 6.

Optimization of assay conditions showed that 10.5 μM CMCT was sufficient to obtain a strong band at C2507, indicative of a modified U2506, and that 1 μM L-**Onc** almost entirely suppressed the band, reflecting specific binding near U2506 (Figures 2A,B and S2–S5). The experiment with 1 μM D-**Onc** had no effect on the band intensity, indicating that the enantiomer was not interacting with U2506. The same effect occurred with DL7-**Onc** with a single chirality switch at position 7, confirming that even small stereochemical alterations could abolish ribosome binding. Accordingly, the fully stereorandomized *sr*-**Onc**, as well as *sr14Nterm*-**Onc**, also did not protect U2506 from CMCT modification, in line with their lack of activity in full MHB. In terms of analogs with preserved activity in full MHB, U2506 was indeed protected from CMCT modification by the analogs with either d-enantiomeric or stereorandomized residues near the C-terminus (D5Cterm-**Onc**/*sr5Cterm*-**Onc**, D8Cterm-**Onc**/*sr8Cterm*-**Onc**, and D9Cterm-**Onc**/*sr9Cterm*-**Onc**), indicating that they bound the ribosome at the same location as L-**Onc**.

Further indication of ribosome targeting was provided by time-kill experiments on *E. coli* and *K. pneumoniae* cells, which showed that active analogs (*sr5Cterm*-**Onc**, *sr8Cterm*-**Onc**, *sr9Cterm*-**Onc**, D5Cterm-**Onc**) had comparable kinetics to L-**Onc**, which, as other ribosome-targeting antibiotics including **AZM**, acted bacteriostatically, in contrast to the membrane-disruptive peptide **ln69** acting rather fast (Figure 2C,D). Furthermore, transmission electron microscopy (TEM) images of *E. coli* and *K. pneumoniae* cells exposed to the compounds in full MHB showed similar morphological changes as those induced by the ribosome-targeting antibiotic **AZM**, including part of the inner membrane detached from the outer membrane leaving a large void, small intracellular vesicles, and a few membrane perturbations (Figure S6).

### Stereorandomized Oncocins Active in Dilute Media Do Not Target Membranes or the Chaperone DnaK

**Figure 3:**
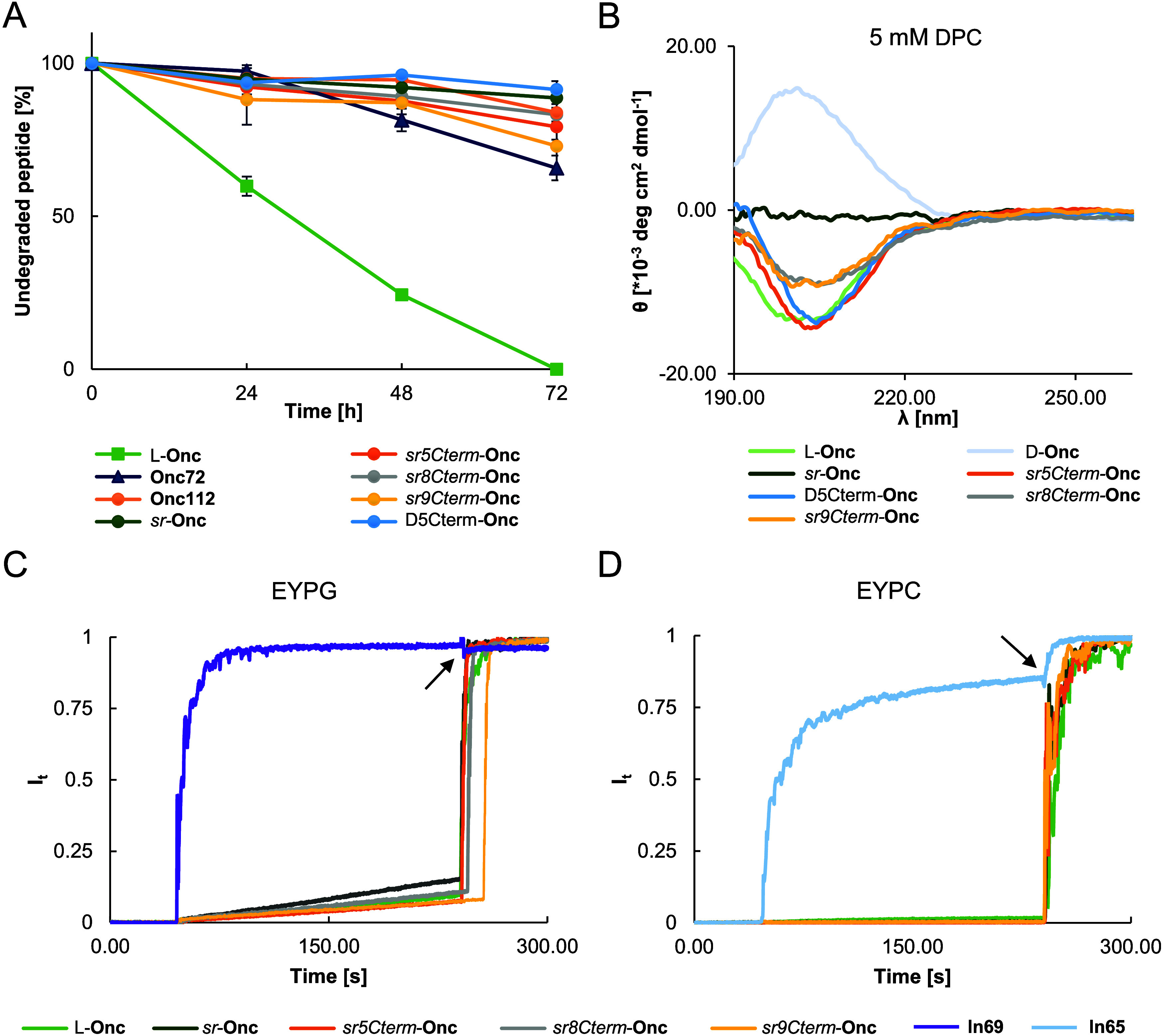
Properties of oncocins active in dilute MHB. (A) Serum stability of L-**Onc** and oncocin analogs in 25% human serum for 24, 48, and 72 h. (B) Circular dichroism (CD) spectra, measured in the presence of 5 mM dodecylphosphocholine (DPC) in 7 mM phosphate buffer at pH 7.4, with 0.1 mg/mL selected peptides. (C, D) Fluorescein leakage assay from vesicles consisting of egg yolk phosphatidyl glycerol (EYPG, C) using AMP **ln69** as a positive control and egg yolk phosphatidyl choline (EYPC, D) using the hemolytic AMP **ln65** as a positive control. The indicated compound at 10 μg/mL was added to a suspension of fluorescein-loaded EYPG or EYPC vesicles suspended in buffer (10 mM tris, 107 mM NaCl, pH adjusted to 7.4). The black arrows indicate the time of addition of Triton X-100 between 240 and 280 s. See Figure S8 for the data at 50 μg/mL.

While antibacterial activities observed with stereorandomized and diastereomeric oncocins in full MHB were well correlated with ribosome binding, the strong activities observed in dilute MHB with almost all analogs, including nonribosome binding sequences *sr*-**Onc** and D-**Onc**, suggested an alternative mechanism of action. Indeed, serum stability assays showed that these analogs were all much more stable than L-**Onc** against proteolytic degradation, as estimated by their stability in human blood serum showing stabilities comparable to the known stabilized analogs **Onc72** and **Onc112**, excluding that the reduced activities of some of the analogs in full MHB might be due to degradation (Figures 3A and S7).

Despite the slow and bacteriostatic kinetics of our analogs typical of intracellular targeting compounds and contrasting with the fast killing of membrane-disruptive AMPs such as **ln69** (see above and Figure 2D), the fact that their activities in dilute MHB did not depend on the SbmA transporter (see above and Table S1) suggested that they might act on the membrane, as shown for the related PrAMP Bac7 for its activity on *P. aeruginosa*.^47,49,50^ To test a possible membrane-disruptive activity, we performed vesicle leakage assay with a selection of *sr*-oncocins; however, these did not show any significant vesicle leakage activity, as measured by fluorescence in either egg yolk phosphatidyl glycerol (EYPG) vesicles mimicking anionic bacterial membranes in comparison to the membrane-disruptive AMP **ln69** as a positive control, or egg yolk phosphatidyl choline (EYPC) vesicles mimicking neutral eukaryotic membranes in comparison with the hemolytic AMP **ln65** (all L-version of **ln69**)^43^ as a positive control (Figures 3C,D and S8). The compounds were also all nonhemolytic (Table 1 last column).

Consistent with the absence of membrane-disruptive effects, circular dichroism (CD) spectra of L-**Onc** and its enantiomer D-**Onc** showed an unordered conformation under conditions typical for inducing folding (Figures 3B, S9, and S10). A similar CD signal for an unordered conformation was also visible in the partially stereorandomized *sr5Cterm*-**Onc**, *sr8Cterm*-**Onc**, and *sr9Cterm*-**Onc** and in diastereomer D5Cterm-**Onc**, while the fully stereorandomized *sr*-**Onc** gave a flat signal, as expected from its racemic nature. These data indicated that L-**Onc** and its analogs indeed did not adopt a helical amphiphilic and potentially membrane-disruptive conformation in contact with membranes, even though they contained several cationic (1 Lys, 5 Arg) and hydrophobic residues (1 Val, 1 Leu, 1 Tyr, 6 Pro) frequently occurring in membrane-disruptive peptides.

Although the chaperone DnaK has been excluded as a possible target by the sensitivity of DnaK null mutants to L-**Onc**,^21^ we further checked if binding to DnaK might explain the activity of stereorandomized oncocins in dilute MHB. This possibility was, however, excluded by the observation that *sr*-**Onc** and D-**Onc**, which were both as antibacterial as L-**Onc** in dilute MHB, showed no detectable binding to DnaK as measured by microscale thermophoresis under conditions where L-**Onc** showed an apparent binding of *K*_D_ ∼ 30 μM (Figures S11–S16).

In the absence of membrane-disruptive effects or DnaK targeting, the activities in dilute MHB most likely reflect a nonspecific aggregation of intracellular contents, as reported for several nonmembrane-disruptive peptoids,^45^ including **EB9**, which, similarly to oncocin, shows increased activity in dilute MHB (Table 1).^44^ The dilute MHB conditions probably lead to enhanced uptake of peptides, leading to a much higher intracellular concentration than occurs in full media, under which conditions this unspecific mechanism of action can occur. In full MHB by contrast, cellular uptake might be much less efficient, leading to a rather low intracellular concentration sufficient to induce ribosome binding for L-**Onc** and analogs with preserved L-chirality in the critical *N*-terminal region but insufficient to enable the nonspecific effect observed across all oncocin analogs tested.

### Activity against Multidrug-Resistant Bacteria Requires Ribosome Binding and Dilute Medium Conditions

To further probe the activities of partially and fully stereorandomized oncocins, we investigated activities against the virulent *P. aeruginosa* strain PA14 and its **PMB**-resistant derivatives PA14 4.13, PA14 4.18, and PA14 2P4,^51^ the *P. aeruginosa* clinical isolates ZEM-1A and ZEM9A, as well as the carbapenem-resistant *K. pneumoniae* OXA-48 and the gut bacterium *Enterobacter cloacae* (Table 2). While the membrane-active compounds **PMB** and **ln69** were quite active across the entire panel under both full MHB and dilute MHB, the references L-**Onc**, **Onc72**, and **Onc112** as well as **AZM** were almost entirely inactive against these bacteria in full MHB and required dilute MHB to show significant activities. Similarly, the partially stereorandomized or D-analogs *sr5Cterm*-**Onc**, *sr8Cterm*-**Onc**, *sr9Cterm*-**Onc**, and D5Cterm-**Onc**, which targeted the ribosome, also showed activities against these strains in dilute medium.

**Table 2:** Activity of *sr*-Oncocin against MDR Bacteria

compound	*P. aeruginosa* PA14	*P. aeruginosa* PA14 4.13 (phoQ)^b^	*P. aeruginosa* PA14 4.18 (pmrB)^b^	*P. aeruginosa* PA14 2P4 (pmrB)^b^	*P. aeruginosa* ZEM-1A	*P. aeruginosa* ZEM9A	*K. pneumoniae* OXA-48	*E. cloacae*
MIC (μg/mL)^a^								
full MHB/12.5% MHB								
L-**Onc**	>64/32	>64/16	>64/32	>64/16	>64/8–16	>64/32	32/2	32/1
**Onc72**	>32/32	>32/32	>32/32	>64/16–32	>64/16^c^	>64/32–64	64/2	32–64/1
**Onc112**	>32/16	>32/16	>32/16	>64/4–8	32/8^c^	>64/8	8/1	2/1
**AZM**	32/32	32/16–32	32/32	32–64/16	64/<0.5	>64/32–64	8–16/8	16/8
**PMB**	<0.5/1	2/2	2/1	4/1	16/8	>64/2	1–2/1–2	2/1–2
**ln69**	2/2	4/2	16/2	32/4	1/4	4–8/2	4/4	4/2
**EB9**	>32/4	>32/16	>32/16	>64/4	>64/32	>64/4	>64/64	64/4
*sr5Cterm*-**Onc**	>64/8	64/8	64/8	>64/2	64/4–8	>64/8	32/4	32/1
*sr8Cterm*-**Onc**	>64/8	64/8	64/8	>64/2	64/4–8	>64/8	32/16	>64/0.5
*sr9Cterm*-**Onc**	>64/8	64/8	64/8	>64/4	64/4–8	>64/8	32/4	32/1
*sr14Nterm-***Onc**	>64/64	>64/32	>64/32	>64/16	>64/>64^c^	>64/64	>64/>64	>64/8
*sr*-**Onc**	>64/32	>64/16	>64/32	>64/16	>64/64^c^	>64/32	>64/>64	>64/8
D5Cterm-**Onc**	>64/8	64/8	64/8	>64/2	32/4–8	>64/2	32/4	32/1
DL7-**Onc**	>32/>32	>32/>32	>32/>32	>64/32	>64/>64^c^	>64/>64	>64/>64	>64/32
D-**Onc**	>64/32	>64/16	>64/16	>64/8	>64/>64^c^	>64/32	>64/64	>64/16

a Minimum inhibitory concentration (MIC, μg/mL) was determined on the indicated MDR strains in full and 12.5% Müller–Hinton medium, both at pH 7.4, after incubation for 16–20 h at 37 °C.

b Strains carrying spontaneous mutations at indicated genes leading to polymyxin B resistance. Values represent two different duplicate MIC determinations.

c Determined based on a single measurement (in duplicates).

By contrast, the nonribosome targeting analogs *sr*-**Onc**, *sr14Nterm*-**Onc**, DL7-**Onc**, and D-**Onc** were almost entirely inactive against all strains in this panel under both conditions. This effect suggests that these difficult bacteria limit peptide uptake more strongly than the reference strains tested, such that even reaching the low intracellular concentrations sufficient for ribosome inhibition requires a stimulated uptake enabled by the dilute MHB.

## Discussion

### Stereorandomized and Homochiral Oncocins Show Comparable Purities

While many small molecule drugs, polymers, and even certain natural products are racemates or a mixture of stereoisomers if multiple chiral centers are undefined, peptides are generally considered as only homochiral molecules with well-defined L- or D-chirality at every amino acid position. As we recently reported, however, stereorandomized (*sr-*) peptides obtained by SPPS using racemic amino acids can be purified as single-peak, single-mass product by preparative HPLC and are almost indistinguishable from homochiral peptides except for their generally better solubility and altered CD spectra. The fully or partially stereorandomized oncocins prepared here confirmed our previous observations, as these sequences provided homogeneous and well-behaved peptides, although in the case of *sr6Cterm*-**Onc**, we observed a peak splitting pattern by HPLC but with a single mass. As expected, the CD spectrum of *sr*-**Onc** was flat, and the CD spectra of partially stereorandomized analogs showed decreased intensities compared to L-**Onc** in relation to the number of stereorandomized positions (Figure 3b).

### Partial Stereorandomization Is Compatible with Target Binding

Stereorandomized sequences represent mixtures of many possible diastereomers, such that individual diastereomers in this mixture only account for a small and often almost insignificant percentage of the compounds. For example, in the case of L-**Onc** with 19 residues, each diastereomer in *sr*-**Onc** only accounts for 1/524 288 = 0.0002% of the sample, assuming that no diastereoselective peptide coupling occurs during synthesis. Therefore, observing a specific target binding effect in a stereorandomized sequence, which we tested for the first time in the present study, would imply that most diastereomers in the mixture are compatible with target binding.

Here, we found that ribosome binding was compatible with stereorandomization at the *C*-terminus of oncocin in the case of *sr5Cterm*-**Onc**, *sr8Cterm*-**Onc**, and *sr9Cterm*-**Onc**. In the latter case consisting of 512 diastereomers, each diastereomer accounted for 0.2% of the mixture, indicating that most, if not all, diastereomers were compatible with target binding. Surprisingly, stereorandomization could be extended by four positions into the partial sequence previously known to be necessary for target binding without reducing activity, including three residues that are directly visible in the ribosome-bound structure of oncocin (Figure 1b). By contrast, fully stereorandomized *sr*-**Onc** did not bind the ribosome.

Direct evidence that a non-natural D-chirality was indeed compatible with target binding was additionally provided by the fact that D5Cterm-**Onc** and D9Cterm-**Onc** had the same antibacterial and ribosome binding activities as L-**Onc**. Strikingly by contrast, stereorandomization or simply inversion of residues in the target binding region led to a loss of activity and ribosome binding, as observed with D-**Onc**, *sr14Nterm*-**Onc**, and DL7-**Onc** in which inversion of the single leucine residue at position 7 led to an inactive analog, in line with its critical role highlighted in structural studies.^23^ The stereochemical alteration at this residue might not strongly reduce side-chain hydrophobic contact but could alter the H-bonds between the peptide backbone and U2506 of the ribosome, which is well visible in the reported structure (Figure 1A).

### Stereorandomized Sequences Resist Proteolytic Degradation

The presence of d-enantiomeric residues in stereorandomized sequences may lead to resistance to proteolytic degradation since proteases are generally specific for l-enantiomeric residues. Here, we tested stability in human serum and found that our *sr*-oncocins were essentially stable to degradation over 24 h to the same extent as the known analogs **Onc72** and **Onc112**, while L-**Onc** was rapidly degraded (Figure 3a). These observations extend our previous report on stereorandomized antimicrobial peptides, which were also resistant to serum degradation,^5^ and suggest that such resistance to degradation should be possible with most stereorandomized peptides although they only contain 50% D-residues or less.

### Stereorandomization Provides Mechanistic Insights

Previous studies with L-**Onc** established that its antimicrobial activity strongly increases in dilute culture media, an effect attributed to the induction of peptide uptake mechanisms.^9,35,36^ Our present study with stereorandomized oncocins confirmed the activity increase in dilute media but showed that these activities were preserved in an *E. coli* mutant lacking the SbmA transporter. Strikingly, most diastereomers of L-**Onc** including the fully stereorandomized *sr*-**Onc** showed the same level of activity against various bacteria in a dilute medium, independently of whether they bound to the ribosome or not. This was particularly striking for *sr*-**Onc**, the enantiomeric D-**Onc**, and DL7-**Onc**, which did not bind the ribosome but were as active as L-**Onc** in a dilute medium.

The observed activity patterns indicated that ribosome binding was correlated with activity in full medium, implying that nonribosome binding analogs must kill bacteria by a different mechanism in dilute medium, which is probably unrelated to DnaK inhibition since this chaperone is nonessential,^21^ and no DnaK binding was detected with *sr*-**Onc** and D-**Onc**. Although our data showed that activities in dilute media did not depend on the peptide transporter SbmA, possibly indicating a membrane targeting mechanism, the slow bacteriostatic kinetics were clearly different from the fast killing typically observed with membrane-disruptive AMPs. We therefore propose that our stereorandomized oncocins might act by aggregation of intracellular contents after entering bacteria, in a mechanism similar to that reported to nonmembrane-disruptive peptoids such as **EB9**.^44,45^ This proposal also takes into account the structural similarities between PrAMPs and peptoids, which both have fewer amide NH groups than peptides and facilitated cellular uptake,^52,53^ implying that they might be able to enter bacteria independently of a specific transporter such as SbmA. This also implies that ribosome binding oncocins including the natural L-**Onc** act by a dual mechanism in dilute media. In full media, by contrast, only a very small amount of the peptide might enter the bacteria, which would be insufficient for intracellular aggregation but sufficient to inhibit the ribosome, even for the active, partially stereorandomized analogs. In the case of more difficult bacteria, such as PA14, the uptake seems to be even more limited, restricting activity to ribosome-targeting oncocins in dilute MHB.

## Conclusions

Here, we showed the first example that partial stereorandomization of a bioactive peptide can be compatible with binding to its target while protecting the sequence against degradation in serum. Specifically, we found that the 19-residue PrAMP oncocin, which inhibits the ribosome by binding to the exit tunnel via its 14 *N*-terminal residues, retains ribosome binding and antibacterial activity against Gram-negative bacteria such as *E. coli* and *K. pneumoniae* when up to 9 *C*-terminal residues are stereorandomized (*sr9Cterm*-**Onc**), which includes 4 of the 14 *N*-terminal residues reported to be essential for its activity. By contrast, full sequence stereorandomization to *sr*-**Onc** abolished ribosome binding, similar to the case for the enantiomer D-**Onc** and further diastereomers containing D-residues in the ribosome binding stretch, such as DL7-**Onc**. Stereorandomized analogs were resistant to serum degradation.

Investigating stereorandomized analogs of oncocin revealed new aspects of its mechanism of action. Indeed, *sr*-**Onc** and all stereorandomized and diastereomeric oncocin analogs investigated here surprisingly retained antibacterial activities against *E. coli* and *K. pneumoniae* in dilute growth media, which are conditions known to enhance the activity of L-**Onc** by stimulating peptide uptake, and even showed strong activities against *P. aeruginosa* and *A. baumannii*. Since many of these analogs did not bind to the ribosome, we attribute their broad antibacterial effect in dilute media to the aggregation of intracellular contents, which seems to require high intracellular concentrations that can only be reached when the uptake is stimulated.

Considering that target binding by most bioactive peptides does not involve all residues in the sequence, partial stereorandomization of nonessential positions as reported here might prove generally useful for property optimization as well as for mechanistic studies.

## Experimental Section

### Peptide Synthesis

Reagents, analytical methods, and synthetic procedures have been detailed in earlier publications.^43,44^ For SPPS of stereorandomized sequences, a 1:1 mixture of Fmoc-protected L- and D-amino acids were used at each stereorandomized position as described earlier.^5^ All compounds were >95% pure by HPLC.

### Further Assays

Bacterial growth assay (Figure S1), transmission electron microscopy (Figure S6), serum stability assay (Figure S7), circular dichroism spectral recording (Figures S8 and S9), antimicrobial and hemolysis activity assays (MIC and MHC), and vesicle leakage assay were carried out as described in earlier publications.^43,44^

### Ribosome Footprinting

#### Isolation of Ribosomes

A 5 mL culture of *E. coli* MG1655 (WT-cells) was grown in LB overnight (220 rpm, 37 °C). One liter LB medium was inoculated, and bacteria were grown to OD = 0.6 (220 rpm. 37 °C); cells were centrifuged in centrifuge bottles, and the pellet was resuspended in ice-cold water and transferred in 50 mL falcon tubes. Falcon tubes were centrifuged, the supernatant was removed, the pellet was resuspended in 15 mL polysome buffer (TRIS/HCl pH 7.5 20 mM, NH_4_Cl 100 mM, MgCl_2_ 10 mM, EDTA 0.5 mM, β-mercaptoethanol 60 mM), frozen dropwise in N_2_ (l), and stored at −80 °C. The cells were ground in CryoMil with the standard program (1 min precooling, 2 min grinding 5/s, 2 min cooling, 2 min grinding 5/s). The powder was transferred in 50 mL falcon tubes containing N_2_ (l) and stored at −80 °C. The lysates were thawed with a water bath (30 °C), put on ice, transferred to precooled Eppendorf tubes, and centrifuged (5000 g, 2 min, 4 °C). The clear lysate was transferred to new precooled tubes, centrifuged (14 000 g, 10 min, 4 °C), and the supernatant was transferred to new precooled tubes. 300 mL of clear lysate was loaded on a 10–50% sucrose gradient in CMCT buffer (K-borate buffer pH 8 80 mM, MgCl_2_ 25 mM, NH_4_Cl 100 mM) and ultracentrifuged (3 h, 234 050 g, 4 °C). The fractions containing monosomes and polysomes were collected and ultracentrifuged overnight (16 h, 260 800 g, 4 °C). The supernatant was discarded, and the pellet was washed with ice-cold CMCT buffer. The ribosomes were resuspended in 100 μL CMCT buffer with a mini magnetic stirring bar and stored on ice. The concentration of ribosomes was measured in 1/200 dilution in water.

#### CMCT Labeling

Ribosomes (5 pmol) were mixed with 8 μL of CMCT buffer, the compound of interest 1 μM, and water to a final volume of 20 μL in a precooled tube and incubated at 25 °C for 10 min. 20 μL of CMCT (10.5 μM) in water was added and incubated at 25 °C for 30 min. The reaction was stopped with 160 μL of EDTA (30 μM), and the samples were stored on ice.

#### Simple Hot Acid Phenol Extraction

160 μL portion of RNA resuspension buffer and 40 μL of 10% SDS were added, and the samples were vigorously resuspended. 400 μL of prewarmed acid phenol was added, and the samples were incubated on a thermomixer (5 min, 1200 rpm, 65 °C). The samples were put on ice for 5 min and centrifuged (5 min, 14 000 g, RT). The watery phase was transferred to a new precooled tube, 400 μL of phenol–chloroform–isoamyl alcohol (4 °C) was added, and the samples were mixed (5 min, 1200 rpm, RT). The samples were centrifuged again (5 min, 14 000*g*, RT), and the watery phase was transferred to another precooled tube. 34 μL of 3 M NaOAc pH 5.5, 0.8 μL of Glycoblue and 400 μL of isopropanol were added, and the samples were incubated for 3 h at −80 °C. The samples were then centrifuged (40 min, 21 000*g*, 4 °C), and the pellet was washed with ice-cold 70% EtOH. The residual liquid was removed, and the samples were dried (5 min, 37 °C). The samples were resuspended in 20 μL of water, the concentration was measured, and the samples were −80 °C.

#### Primer Labeling

Primers designed to probe the positions 2506 of the 23S rRNA (5-CCCTTGGGACCTACTTC-3′) were labeled with radioactive phosphate (γ^32^ P-ATP). It is a phosphorylation of polynucleotides. To prepare primers for one reaction, 1 μL of primer (0.3 μM), 0.4 μL of 5× PNK-buffer, 0.25 μL of water, 0.2 μL of γ^32^ P-ATP, and 0.15 μL of T4 PNK were mixed and incubated at 37 °C. The enzyme was inactivated at 92 °C for 2 min and then stored at −20 °C in a shielded box.

#### Primer Annealing and Extension

The primers were annealed to the rRNA and elongated by reverse transcriptase (AMV RT). 500 ng of RNA was mixed with 2.5 μL of hybridization buffer, 2 μL of the labeled primer, and 3.5 μL of water. The samples were incubated at 92 °C for 5 min and immediately incubated at 42 °C for 30 min. Samples were then spined and stored at room temperature. A mix of 4 μL of 5× extension buffer, 2 μL of dNTP-mix, 3 μL of water, and 1 μL of AMV RT (2U/μL) was prepared. The sequencing lanes mix was prepared in the same fashion but with 0.7 μL of ddNTP and 1.3 μL of dNTP-Mix. The complete mixtures were incubated at 42 °C for 30 min, and the reaction was then stopped by adding 2.5 volumes of stop solution and 180 μL of 100% ethanol. The samples were centrifuged at full speed and 4 °C for 40 min; the supernatant was removed, and the pellet was washed with 80% ethanol and spun again. The supernatant was completely removed, and the pellet was resuspended in 8 μL of loading buffer.

#### Gel Electrophoresis

A 15% TBE/7 M urea 0.4 mm thick gel was prepared by preparing a mixture of 50 mL of acrylamide, 200 μL of 10% APS, and 30 μL of TEMED. The mixture was poured into a gel cassette and allowed to polymerize for 30 min. The gel was prerun at 1200 V/40 mA/300 W for 30 min. The wells of the gel were washed from urea prior to loading. The samples were cooked at 90 °C for 3 min and put on ice before being loaded. The samples were run at 1200 V/30 mA/300 W for 2.5 h. The gel was removed from the cassette and placed against a photo plate for exposure overnight at −20 °C (Figures S2–S5).

### DnaK Experiments

#### Expression and Purification

A plasmid containing the DnaK insert was designed in SnapGene and ordered commercially as a synthetic gene (Figure S11). Chemically competent BL21(DE3) cells were transformed with the plasmid vector (GenScript), and positive transformants were selected on LB + Kanamycin agar. For protein expression, 6 mL of overnight cultures inoculated with a single colony were used to initiate shake-flask expression at 300 mL scale in LB + kanamycin (50 μg/uL) + 1 mM IPTG. The flasks were cultured overnight at 220 rpm, 37 °C, and the bacteria were harvested by centrifugation the next day. Pelleted cells were stored at −20 °C. DnaK was purified by nickel affinity FPLC with a His-Trap HP (Cytiva) using an AKTA prime (GE Pharmacia) according to standard procedures as described by the manufacturer. Crude lysate was obtained by sonication of *E. coli* pellets resuspended in binding buffer (mobile phase buffer A, 20 mM sodium phosphate, pH 7.4, 500 mM NaCl), followed by centrifugation at 20 000*g*, and 0.45 μm syringe-filtration A sample of the unbound eluted protein was collected before elution with the mobile phase buffer B (20 mM sodium phosphate, pH 7.4, 500 mM NaCl, 500 mM imidazole). The collected fractions were analyzed by SDS-PAGE. 10 μL of the combined DnaK fractions adjusted to 2 mg/mL were also added to the gel to assess the protein’s purity in the absence of lane overloading (Figure S12).

#### Microscale Thermophoresis

DnaK labeling was performed using the Nanotemper Monolith His-Tag Labeling Kit RED-tris-NTA Second Generation. For binding check, the target sample, 50 μL of 20 nM His-Tag-labeled DnaK in MST buffer (50 mM TRIS, 150 mM NaCl, 10 mM MgCl_2_, Tween 20 0.05%), and the complex sample 50 μL of 20 nM His-Tag labeled DnaK, 50 μM Oncocin analogs in MST buffer were prepared. The fluorescence variation between the samples was measured in 4 replicates with the Monolith NT.115 device in Monolith NT.115 Premium Capillaries and analyzed with the MOcontrol software (Figures S13–S15).

For binding affinity experiments, 16 samples of L-**Onc** were prepared by performing a 2-fold serial dilution, starting from a maximum concentration of 100 μM in MST buffer, with each sample having a total volume of 10 μL. To each sample, 10 μL of 40 nM His-Tag-labeled DnaK was added, resulting in a final volume of 20 μL per sample. The fluorescence variations between the samples were measured by using the Monolith NT.115 device with Monolith NT.115 Premium Capillaries. The data were analyzed with MOcontrol software to determine the binding affinity between L-onc and His-Tag-labeled DnaK (Figure S16).

## Supporting Information Available

SMILES and activity of all tested peptides (CSV)
Peptide yields; chemical structures; HPLC-MS chromatograms and HRMS spectra for all compounds; bacterial growth curves; time-kill kinetics; hemolysis assay; sequencing gel for ribosome footprinting experiment; TEM images of untreated and treated bacteria with the selected compounds; serum stability curves; vesicle leakage assay; CD spectra and analysis; map of the pET28a T7 expression vector with DnaK inset; figure of SDS-PAGE of his-tagged DnaK purification; binding curves of DnaK; and the selected compounds (PDF)

## Abbreviations Used

AMP: antimicrobial peptide
AMPD: antimicrobial peptide dendrimer
AMV RT: avian myeloblastosis virus reverse transcriptase
APS: ammonium persulfate
ATCC: American type culture collection
ATP: adenosine triphosphate
AZM: azithromycin
CD: circular dichroism
CMCT: 1-cyclohexyl-3-(2-morpholinoethyl) carbodiimide metho-*p*-toluene sulfonate
dNTP: desoxynucleotide triphosphate
ddNTP: dideoxynucleotide triphosphate
DPC: dodecylphosphocholine
EDTA: ethylenediaminetetraacetic acid
EtOH: ethanol
EYPC: egg yolk phosphatidyl choline
EYPG: egg yolk phosphatidyl glycerol
FPLC: fast protein liquid chromatography
HPLC: high-performance liquid chromatography
hRBC: human red blood cell
HRMS: high-resolution mass spectrometry
IPTG: isopropyl β-d-1-thiogalactopyranoside
LB: Luria–Bertani
LUV: large unilamellar vesicle
MDR: multidrug resistant
MIC: minimal inhibitory concentration
MHB: Mueller–Hinton broth
MHC: minimum hemolytic concentration
MRSA: methicillin-resistant *S. aureus*
MST: microscale thermophoresis
Onc: oncocin
PMB: polymyxin B
PNK: polynucleotide kinase
PrAMP: proline-rich antimicrobial peptide
rRNA: ribosomal ribonucleic acid
RT: room temperature
SDS: sodium dodecyl sulfate
SPPS: solid-phase peptide synthesis
*sr*: stereorandom
TEM: transmission electron microscope
TEMED: tetramethylethylenediamine
TBE: tris/borate/EDTA buffer
TRIS: 2-amino-2-hydroxymethylpropane-1,3-diol
WT: wild type
