# Supplementary material for: Stereorandomized Oncocins with Preserved Ribosome Binding and Antibacterial Activity
Source: J Med Chem. 2024 Oct 24;67(21):19448–59. doi: 10.1021/acs.jmedchem.4c01768 (PMC11571207; doi:10.1021/acs.jmedchem.4c01768)
Supplement: Supplementary file 2 — jm4c01768_si_002.pdf [file jm4c01768_si_002.pdf]

# Supporting information

## Stereorandomized oncocins with preserved ribosome binding and antibacterial activity

*Bee Ha Gan,[1) ‡] Etienne Bonvin,[1) ‡] Thierry Paschoud,[1) ‡] Hippolyte Personne,[1)] Jérémie Reusser,[1)] Xingguang Cai,[1)] Robert Rauscher,[1)] Thilo Köhler,[2)] Christian van Delden,[2)] Norbert Polacek[1)] and Jean-Louis Reymond[1)]**

*[1)]Department of Chemistry, Biochemistry and Pharmaceutical Sciences, University of Bern, Freiestrasse 3, 3012 Bern, Switzerland*

*[2)] Department of Microbiology and Molecular Medicine, University of Geneva, Service of Infectious Diseases, University Hospital of Geneva, 1211 Geneva, Switzerland*

*[‡]Equal contribution as first authors*

**jean-louis.reymond@unibe.ch*

## Table of Contents

Peptide characterization ..... 2

Microbiology ..... 62

Hemolysis assay ..... 67

Ribosome Footprinting ..... 68

Transmission electron microcopy ..... 70

Serum stability ..... 72

Vesicle Leakage Assay ..... 73

Circular Dichroism Spectroscopy ..... 75

DnaK experiments ..... 79

## Peptide characterization

**L-Onc** VDKPPYLPRPRPPRRIYNR

From Rink Amide AM resin (357 mg, 0.29 mmol/g), the peptide was obtained as a white foamy solid after preparative RP-HPLC purification (111 mg, 34%).

Analytical RP-HPLC: $t_R$ = 2.39 min (A/D = 100/0 to 0/100 in 10.0 min., λ = 214 nm).

HRMS (ESI+): $C_{109}H_{178}N_{37}O_{24}$ calc./found 2389.3840/2389.3857 Da $[M + H]^+$.

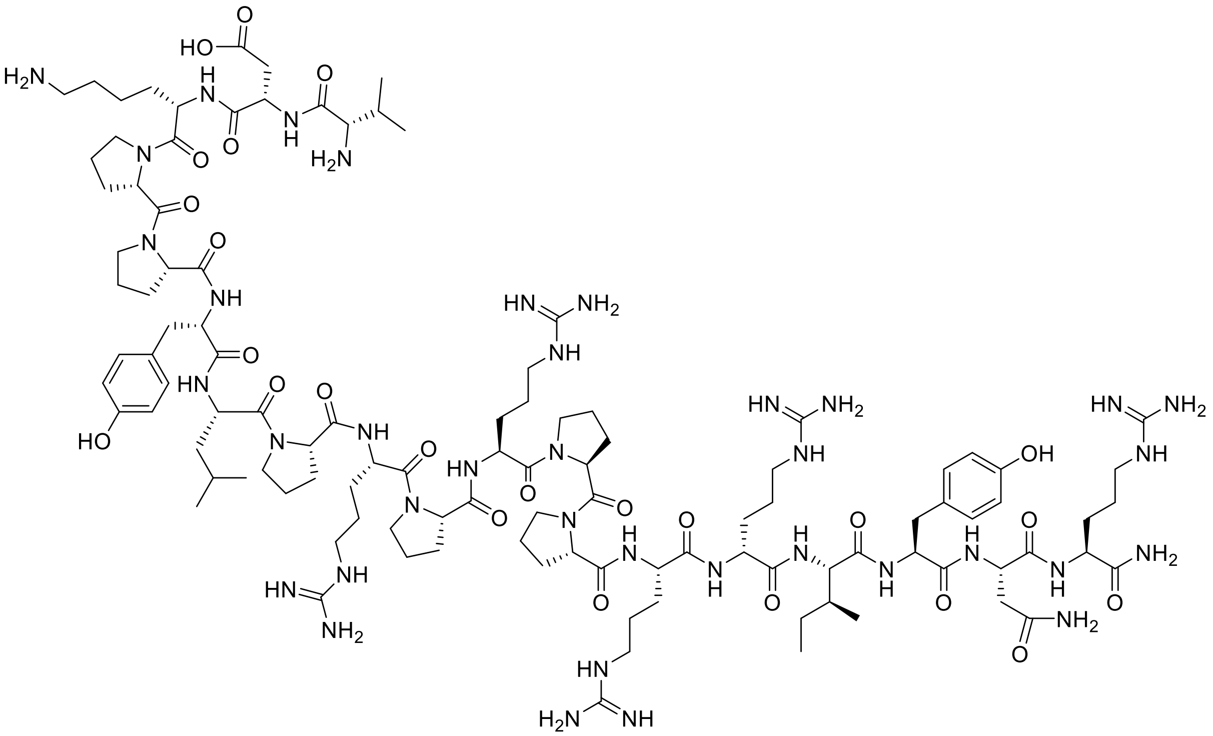

Chemical Formula: $C_{109}H_{177}N_{37}O_{24}$
Exact Mass: 2388.3767
Molecular Weight: 2389.8500

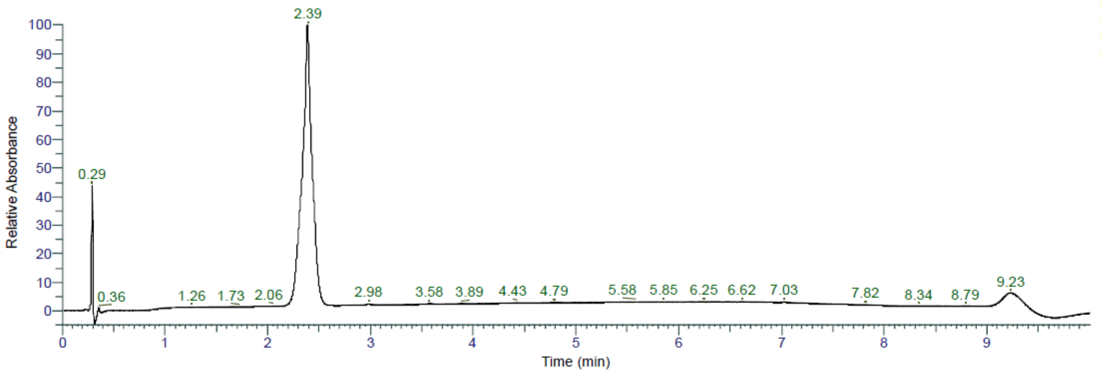

Relative Absorbance
Time (min)
2.39
0.29
0.36
1.26
1.73
2.06
2.98
3.58
3.89
4.43
4.79
5.58
5.85
6.25
6.62
7.03
7.82
8.34
8.79
9.23


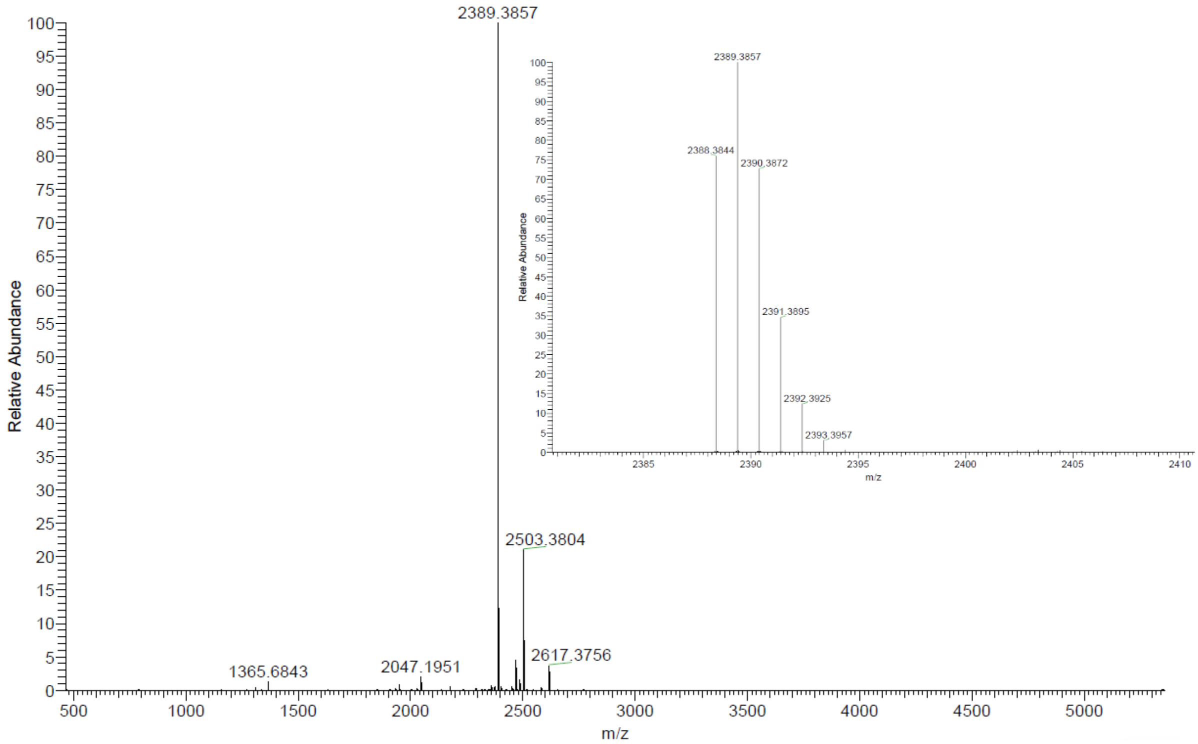

Relative Abundance
m/z
2389.3857
2503.3804
2617.3756
2047.1951
1365.6843
2388.3844
2390.3872
2391.3895
2392.3925
2393.3957

**Onc72** VDKPPYLPRPRPPROIYNO

From Rink Amide AM resin (305 mg, 0.29 mmol/g), the peptide was obtained as a white foamy solid after preparative RP-HPLC purification (109 mg, 40%).

Analytical RP-HPLC: $t_R$ = 2.32 min (A/D = 100/0 to 0/100 in 10.0 min., λ = 214 nm).

HRMS (ESI+): $C_{107}H_{173}N_{32}O_{25}$ calc./found 2305.3384/2305.3336 Da $[M + H]^+$.

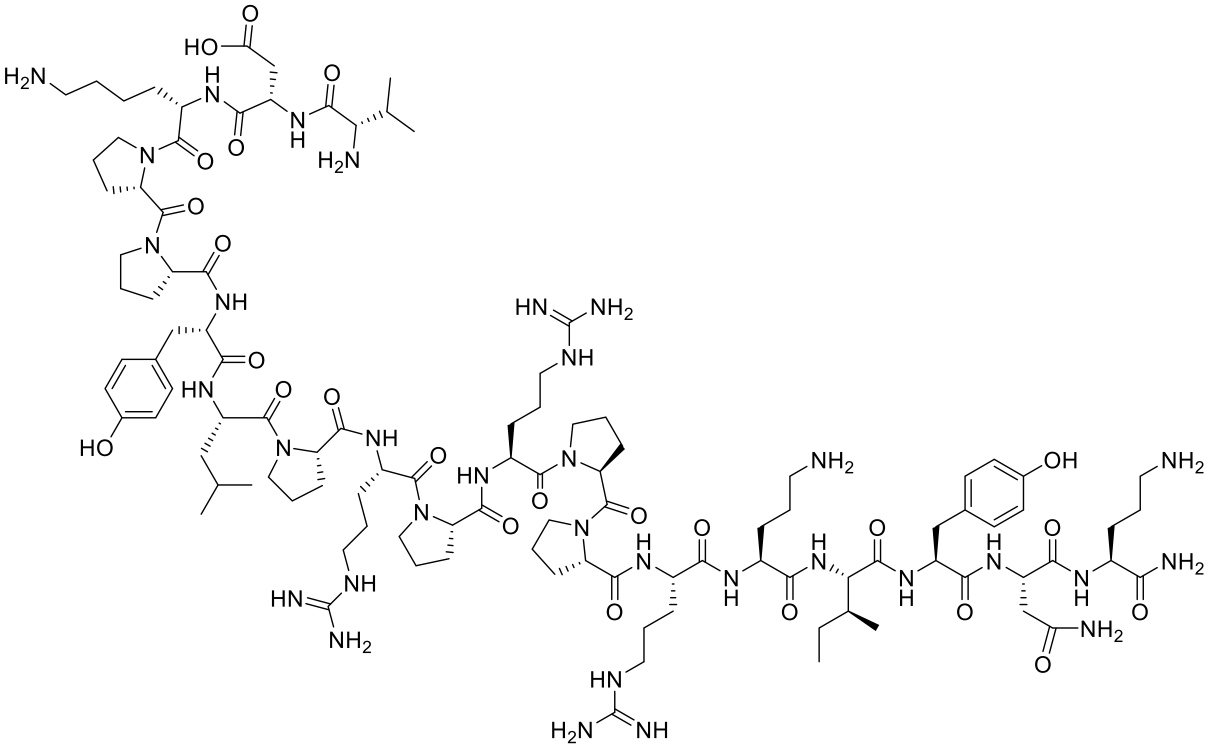

Chemical Formula: $C_{107}H_{173}N_{33}O_{24}$
Exact Mass: 2304.3331
Molecular Weight: 2305.7680

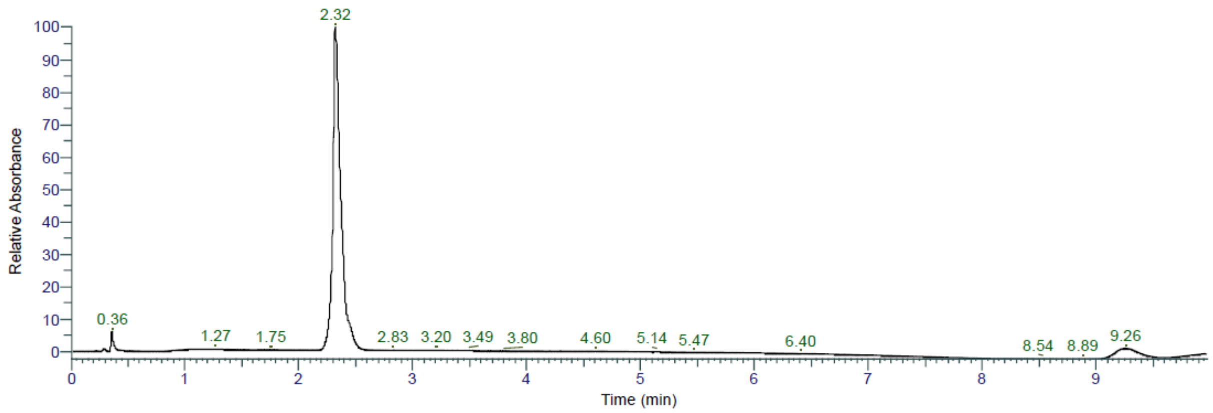
2.32
0.36
1.27
1.75
2.83
3.20
3.49
3.80
4.60
5.14
5.47
6.40
8.54
8.89
9.26
Relative Absorbance
Time (min)

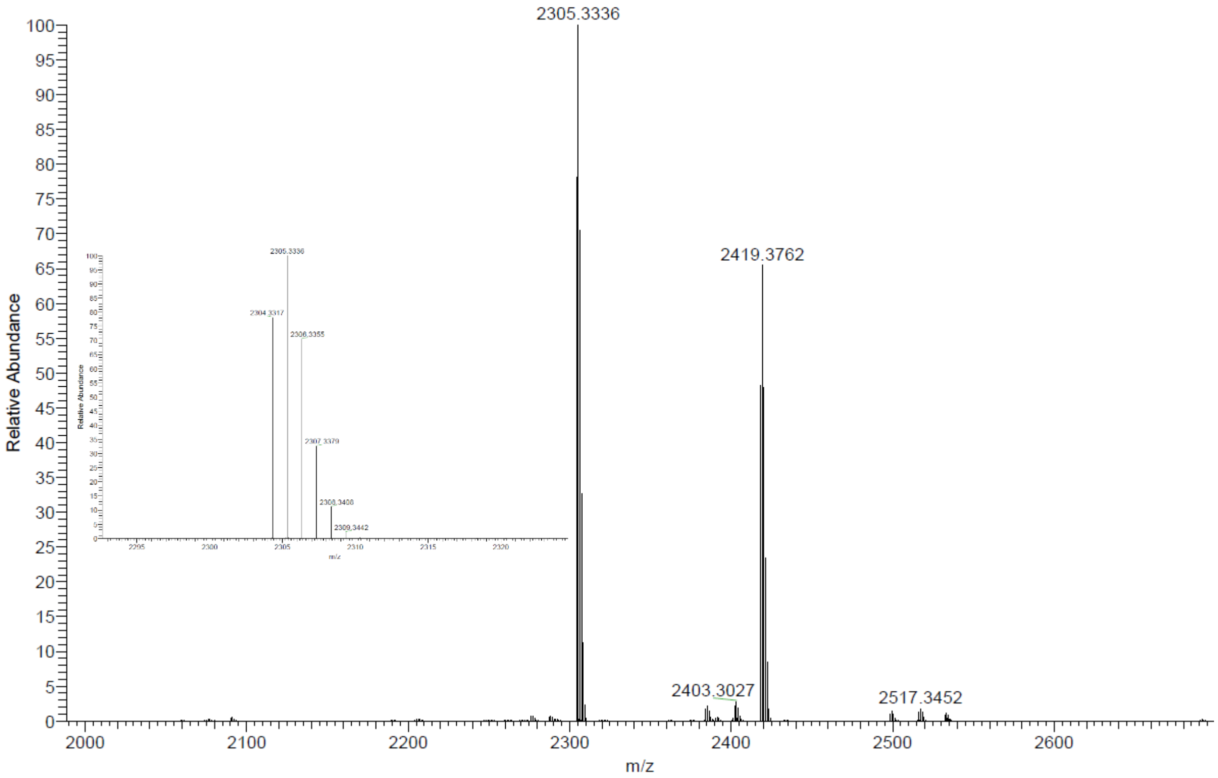
2305.3336
2419.3762
2403.3027
2517.3452
Relative Abundance
m/z
2304.3317
2306.3355
2307.3379
2308.3406
2309.3442

**Onc112** VDKPPYLPRPRPPRrIYNr

From Rink Amide AM resin (300 mg, 0.25 mmol/g), the peptide was obtained as a white foamy solid after preparative RP-HPLC purification (18 mg, 8%).

Analytical RP-HPLC: $t_R$ = 2.35 min (A/D = 100/0 to 0/100 in 10.0 min., λ = 214 nm).

HRMS (ESI+): $C_{109}H_{178}N_{37}O_{24}$ calc./found 2389.3840/2389.3811 Da $[M + H]^+$.

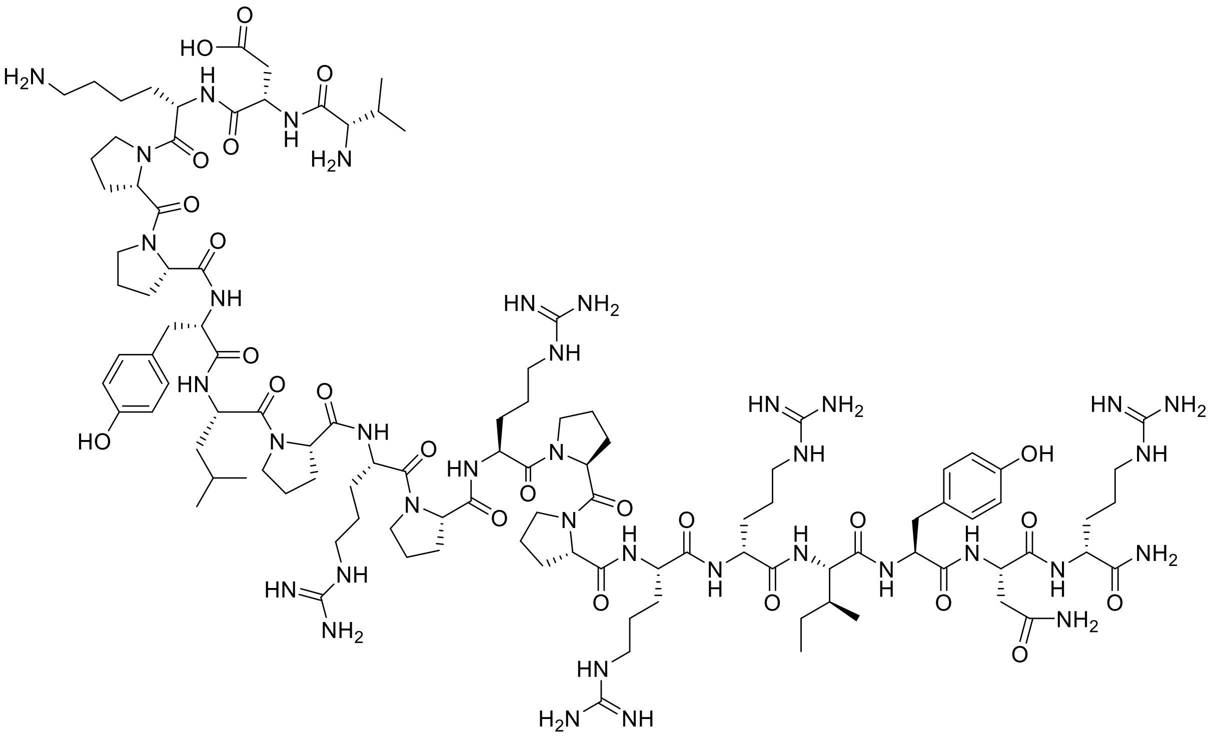

Chemical Formula: $C_{109}H_{177}N_{37}O_{24}$
Exact Mass: 2388.3767
Molecular Weight: 2389.8500

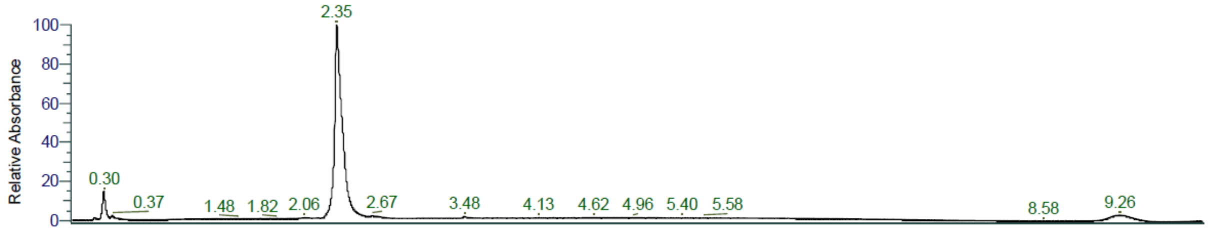
Relative Absorbance
100
80
60
40
20
0
2.35
0.30
0.37
1.48
1.82
2.06
2.67
3.48
4.13
4.62
4.96
5.40
5.58
8.58
9.26

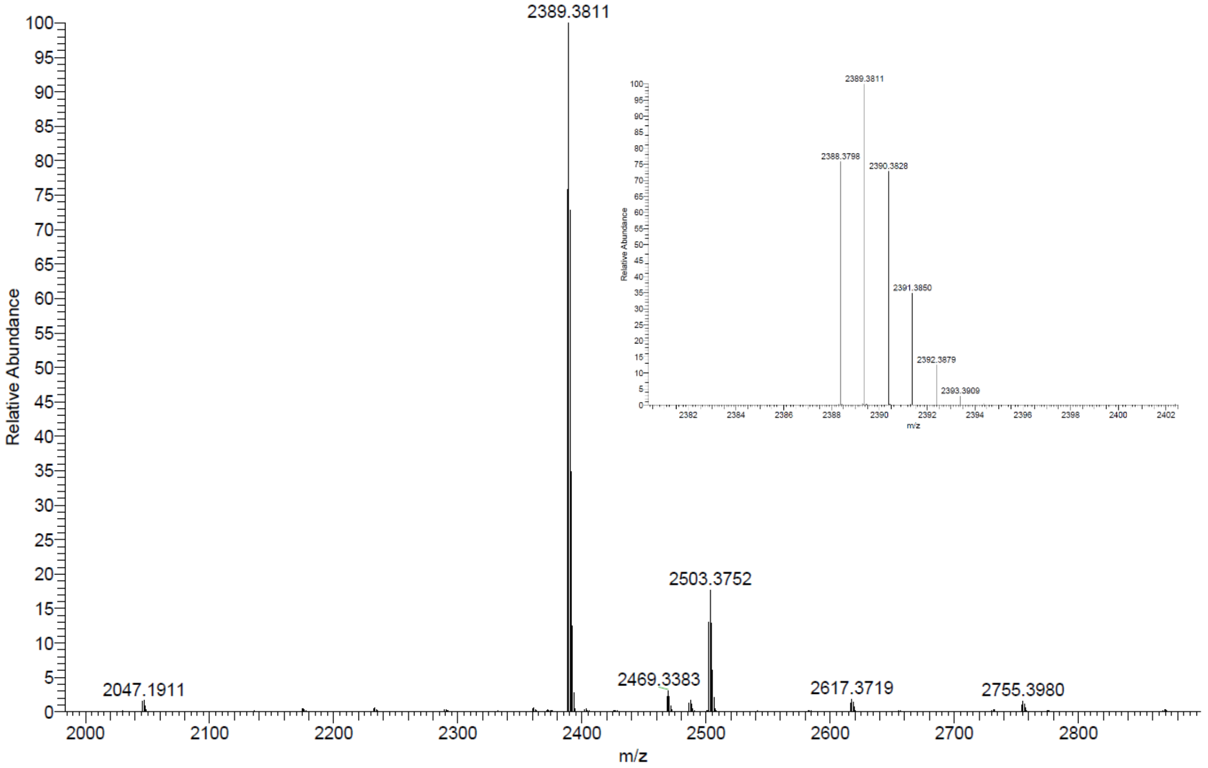
Relative Abundance
2389.3811
2503.3752
2047.1911
2469.3383
2617.3719
2755.3980
2000
2100
2200
2300
2400
2500
2600
2700
2800
m/z
2388.3798
2390.3828
2391.3850
2392.3879
2393.3909

***sr5Cterm*-Onc** VDKPPYLPRPRPPR*RIYNR* (L:D 1:1)

From Rink Amide AM resin (331 mg, 0.29 mmol/g), the peptide was obtained as a white foamy solid after preparative RP-HPLC purification (89 mg, 30%).

Analytical RP-HPLC: $t_R$ = 2.42 min (A/D = 100/0 to 0/100 in 10.0 min., λ = 214 nm).

HRMS (ESI+): $C_{109}H_{178}N_{37}O_{24}$ calc./found 2389.3840/2389.3872 Da $[M + H]^+$.

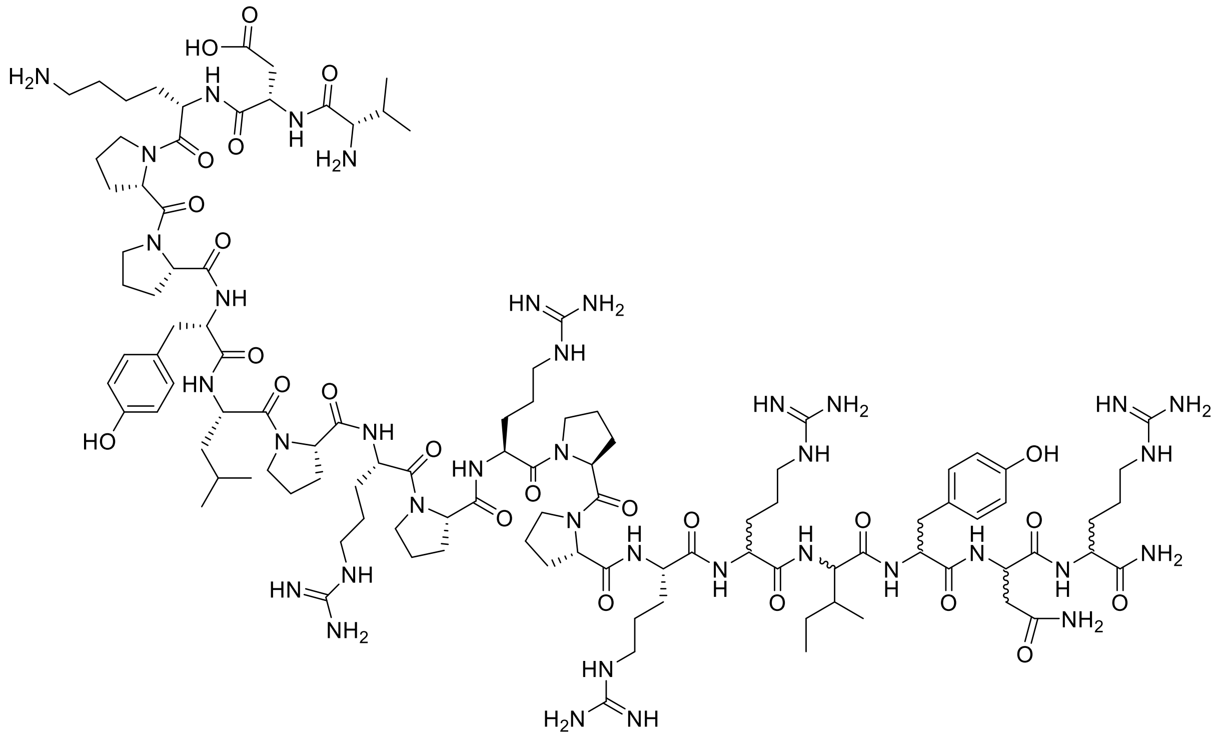

Chemical Formula: $C_{109}H_{177}N_{37}O_{24}$
Exact Mass: 2388.3767
Molecular Weight: 2389.8500

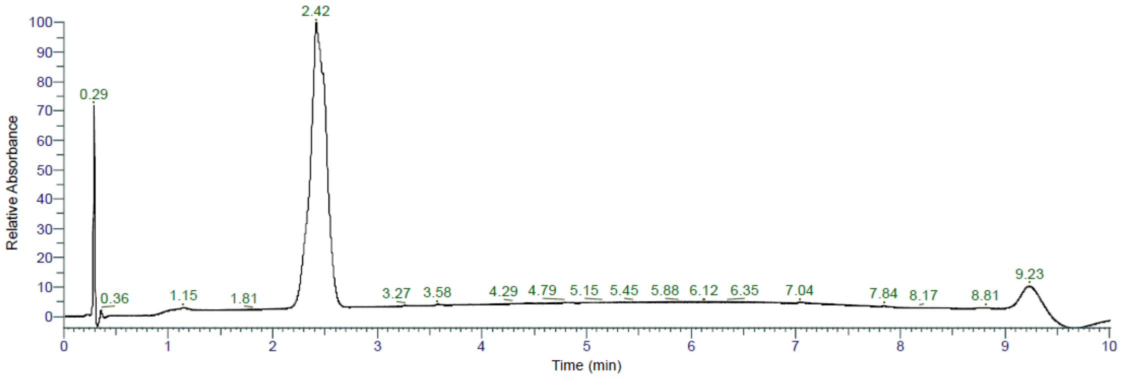
Relative Absorbance
Time (min)
0.29
0.36
1.15
1.81
2.42
3.27
3.58
4.29
4.79
5.15
5.45
5.88
6.12
6.35
7.04
7.84
8.17
8.81
9.23

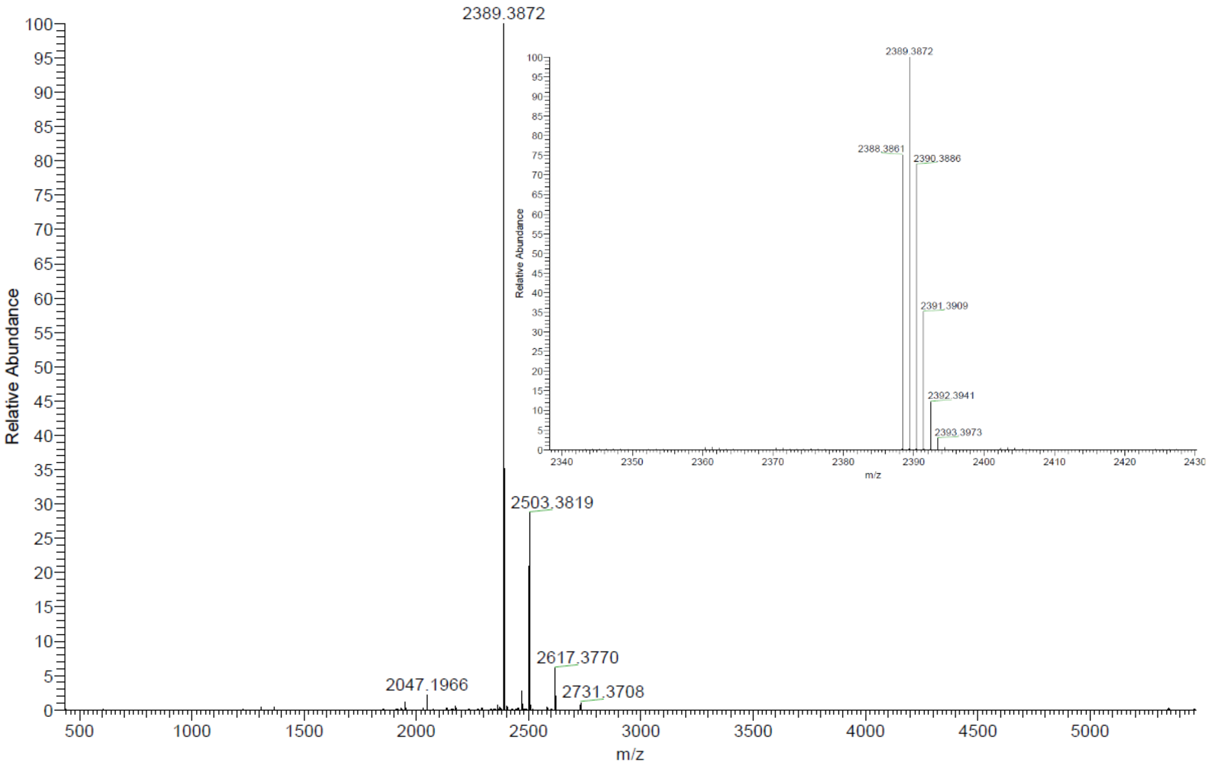
Relative Abundance
m/z
2389.3872
2503.3819
2617.3770
2731.3708
2047.1966
2388.3861
2390.3886
2391.3909
2392.3941
2393.3973

***sr6Cterm*-Onc** VDKPPYLPRPRPP*RRIYNR* (L:D 1:1)

From Rink Amide AM resin (368 mg, 0.29 mmol/g), the peptide was obtained as a white foamy solid after preparative RP-HPLC purification (102 mg, 30%).

Analytical RP-HPLC: $t_R$ = 2.35-2.38 min (A/D = 100/0 to 0/100 in 10.0 min., λ = 214 nm).

HRMS (ESI+): $C_{109}H_{178}N_{37}O_{24}$ calc./found 2389.3840/2389.3770 Da $[M + H]^+$.

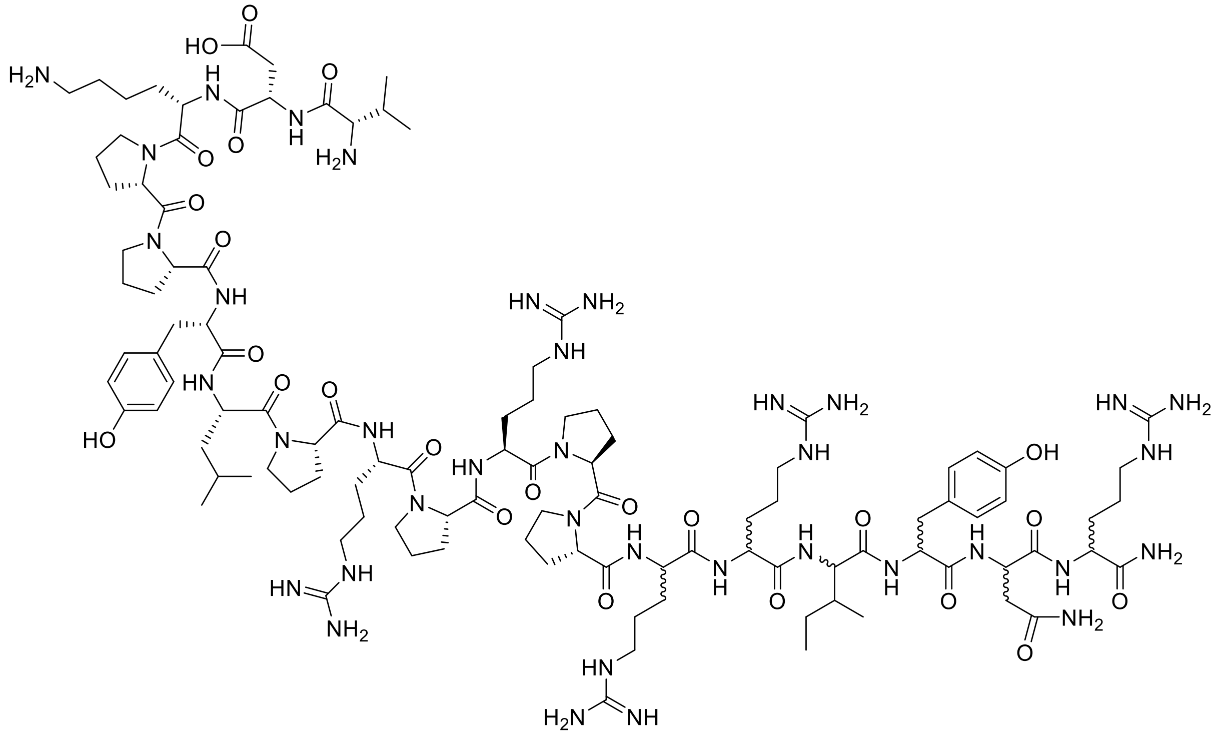


Chemical Formula: $C_{109}H_{177}N_{37}O_{24}$
Exact Mass: 2388.3767
Molecular Weight: 2389.8500

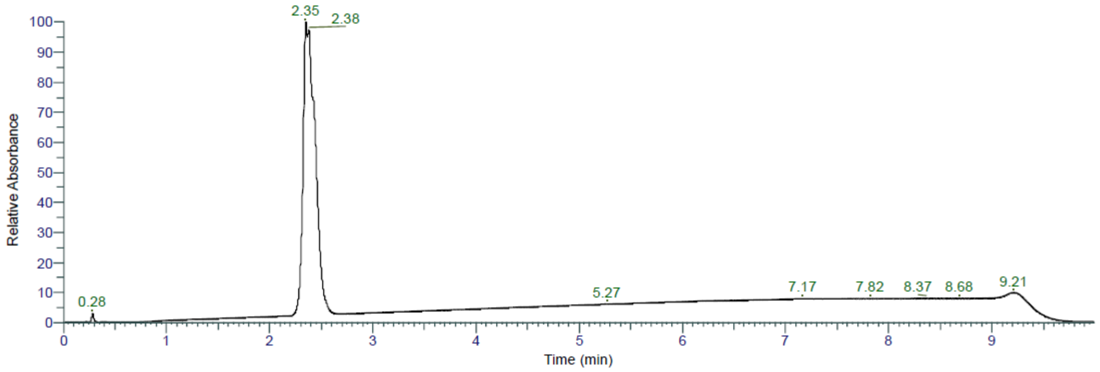
2.35
2.38
0.28
5.27
7.17
7.82
8.37
8.68
9.21
Relative Absorbance
Time (min)

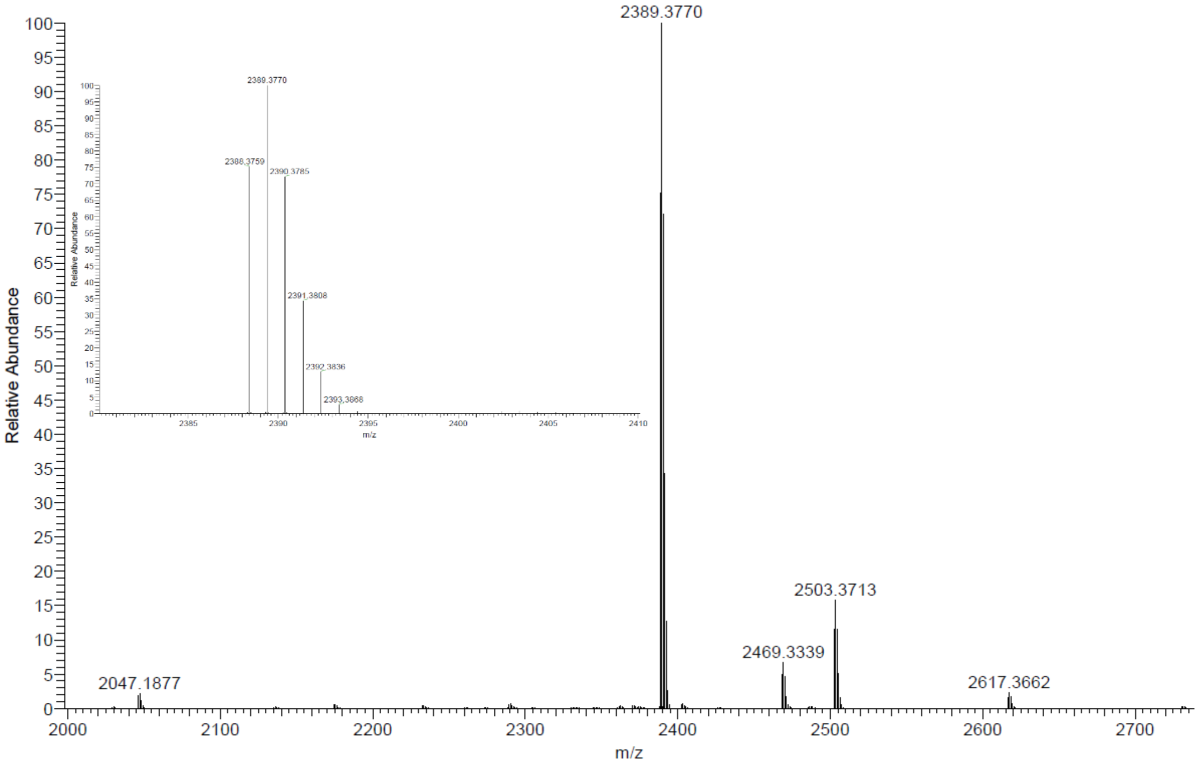
2389.3770
2503.3713
2469.3339
2047.1877
2617.3662
2388.3759
2390.3785
2391.3808
2392.3836
2393.3868
Relative Abundance
m/z

***sr7Cterm*-Onc** VDKPPYLPRPRP*PRRIYNR* (L:D 1:1)

From Rink Amide AM resin (333 mg, 0.29 mmol/g), the peptide was obtained as a white foamy solid after preparative RP-HPLC purification (124 mg, 40%).

Analytical RP-HPLC: $t_R$ = 2.36 min (A/D = 100/0 to 0/100 in 10.0 min., $\lambda$ = 214 nm).

HRMS (ESI+): $C_{109}H_{178}N_{37}O_{24}$ calc./found 2389.3840/2389.3778 Da $[M + H]^+$.

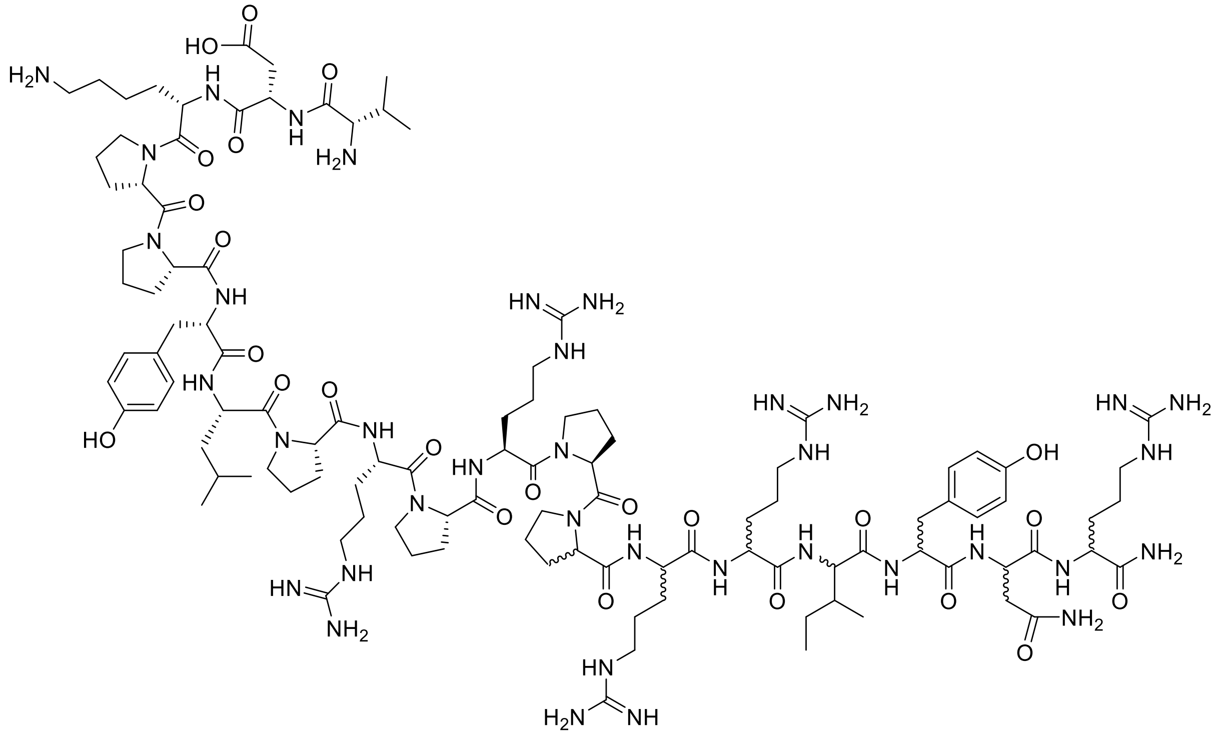

Chemical Formula: $C_{109}H_{177}N_{37}O_{24}$
Exact Mass: 2388.3767
Molecular Weight: 2389.8500

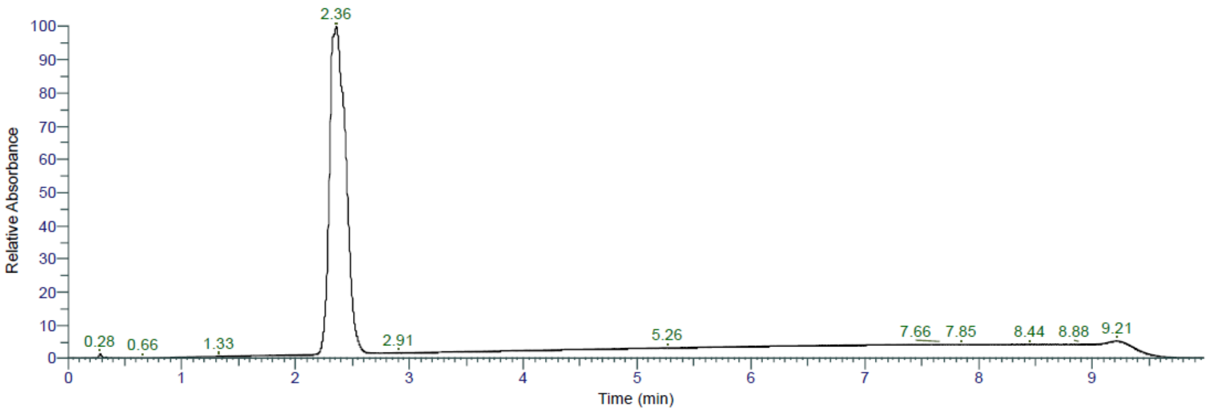
2.36
0.28
0.66
1.33
2.91
5.26
7.66
7.85
8.44
8.88
9.21
Relative Absorbance
Time (min)

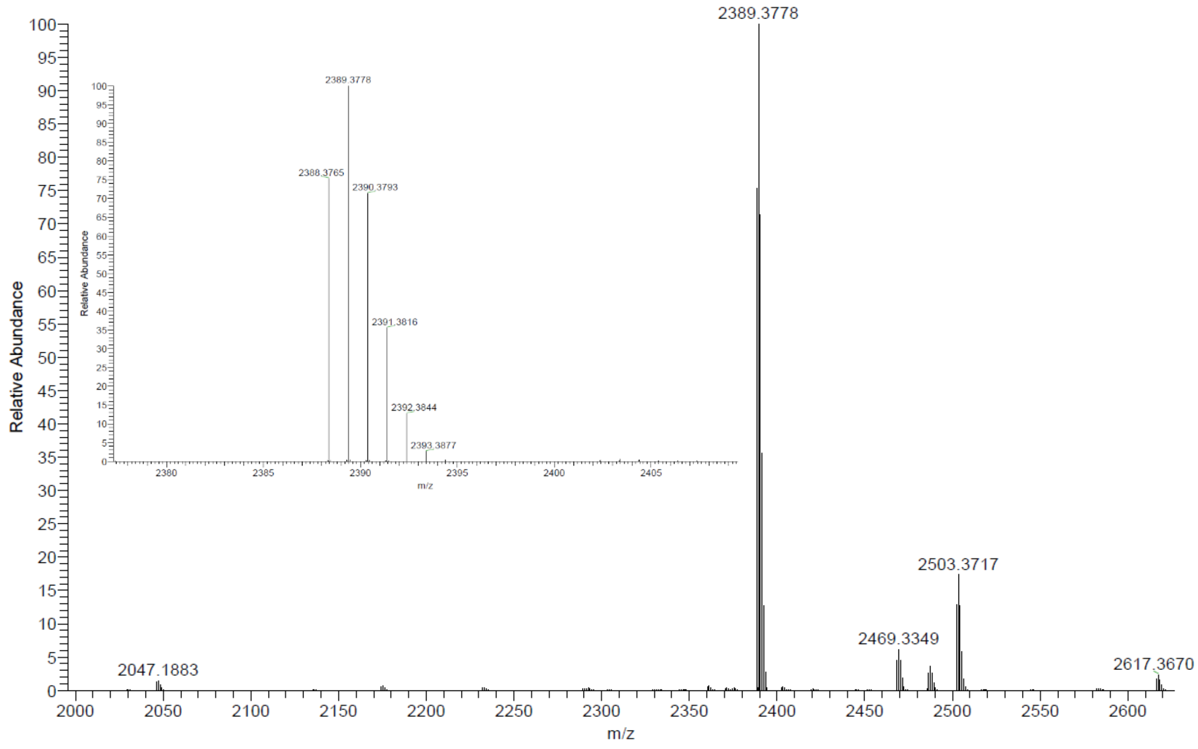
2389.3778
2503.3717
2469.3349
2617.3670
2047.1883
Relative Abundance
m/z
2388.3765
2390.3793
2391.3816
2392.3844
2393.3877

***sr8Cterm*-Onc** VDKPPYLPRPR*PPRRIYNR* (L:D 1:1)

From Rink Amide AM resin (309 mg, 0.29 mmol/g), the peptide was obtained as a white foamy solid after preparative RP-HPLC purification (19 mg, 6%).

Analytical RP-HPLC: $t_R$ = 2.36 min (A/D = 100/0 to 0/100 in 10.0 min., λ = 214 nm).

HRMS (ESI+): $C_{109}H_{178}N_{37}O_{24}$ calc./found 2389.3840/2389.3783 Da $[M + H]^+$.

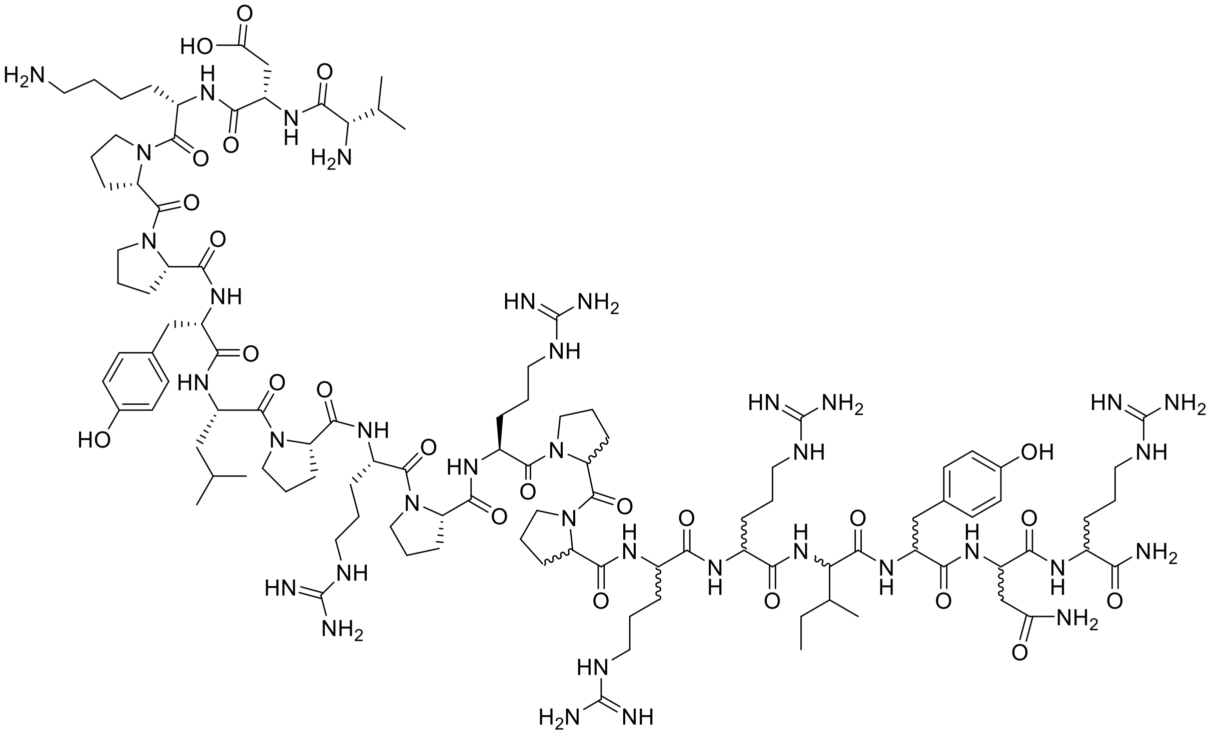

Chemical Formula: $C_{109}H_{177}N_{37}O_{24}$
Exact Mass: 2388.3767
Molecular Weight: 2389.8500

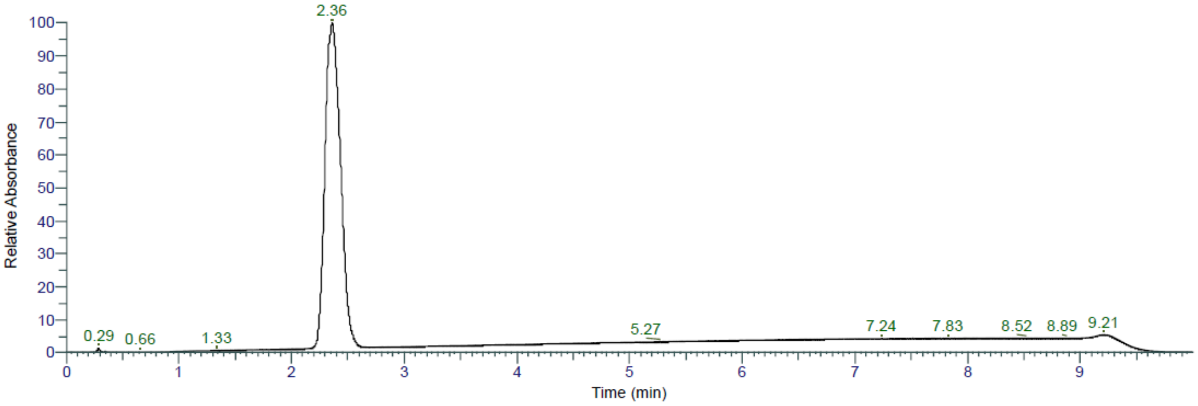

2.36
Relative Absorbance
0.29
0.66
1.33
5.27
7.24
7.83
8.52
8.89
9.21
Time (min)


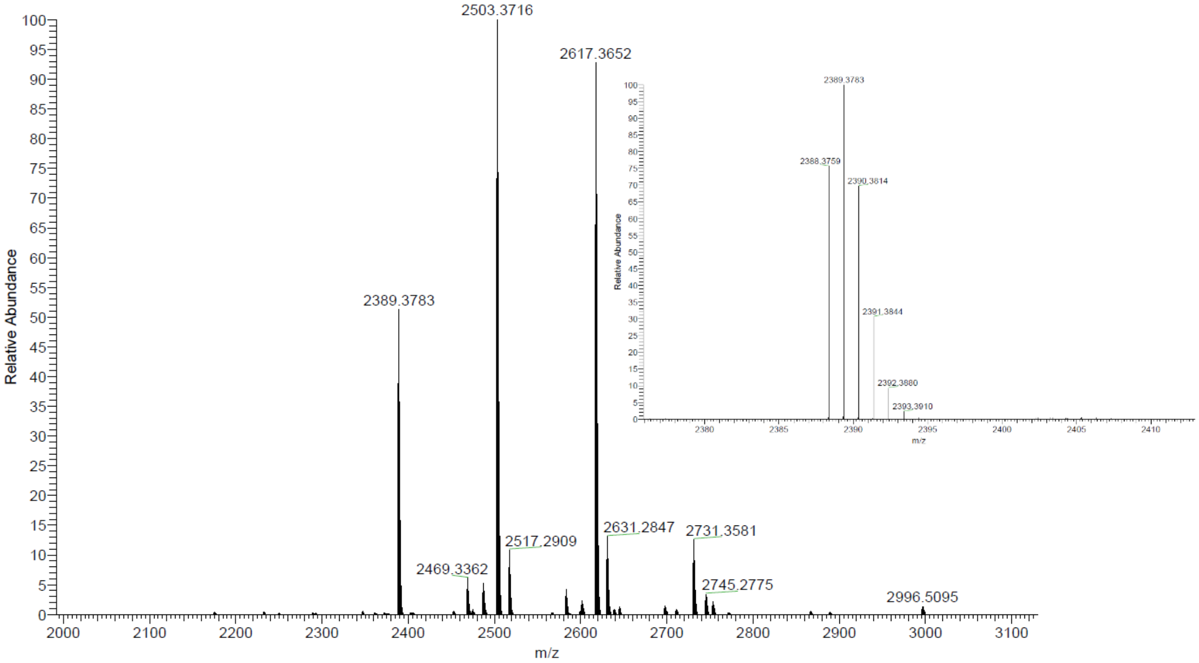

2503.3716
2617.3652
2389.3783
2469.3362
2517.2909
2631.2847
2731.3581
2745.2775
2996.5095
Relative Abundance
m/z

***sr9Cterm*-Onc** VDKPPYLPRP*RPPRRIYNR* (L:D 1:1)

From Rink Amide AM resin (318 mg, 0.29 mmol/g), the peptide was obtained as a white foamy solid after preparative RP-HPLC purification (103 mg, 35%).

Analytical RP-HPLC: $t_R$ = 2.40 min (A/D = 100/0 to 0/100 in 10.0 min., $\lambda$ = 214 nm).

HRMS (ESI+): $C_{109}H_{178}N_{37}O_{24}$ calc./found 2389.3840/2389.3755 Da $[M + H]^+$.

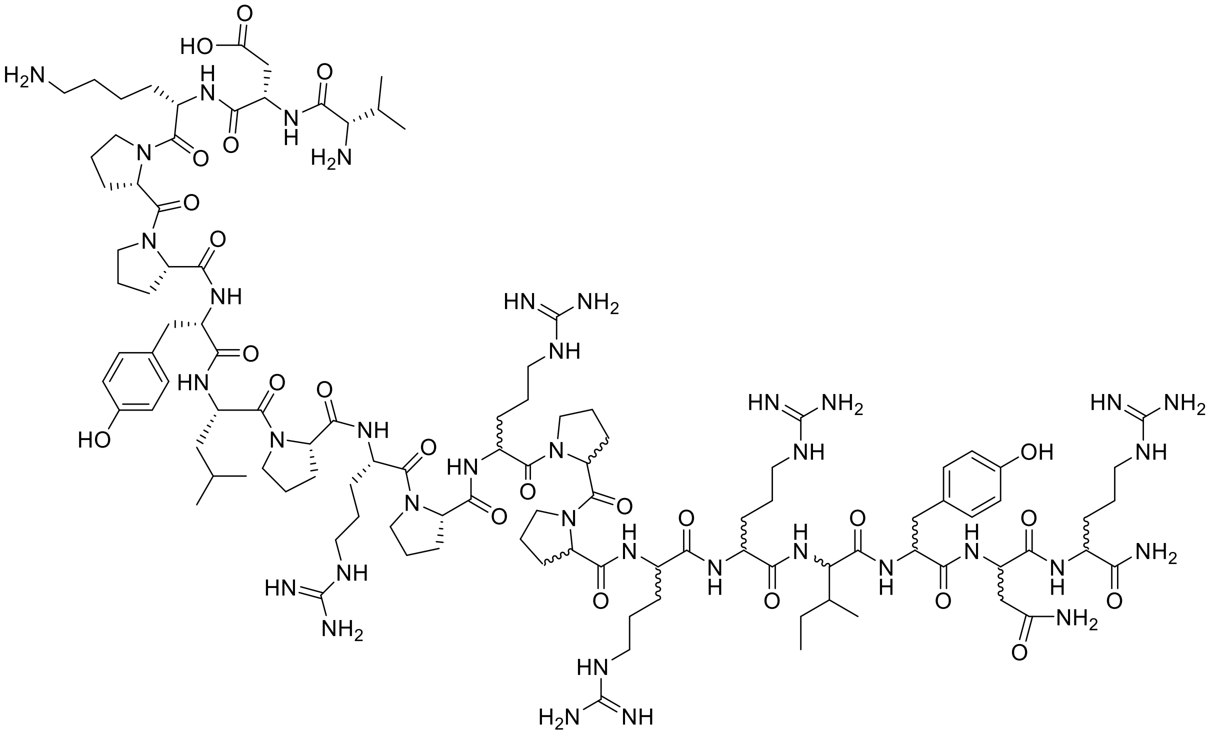

Chemical Formula: $C_{109}H_{177}N_{37}O_{24}$
Exact Mass: 2388.3767
Molecular Weight: 2389.8500

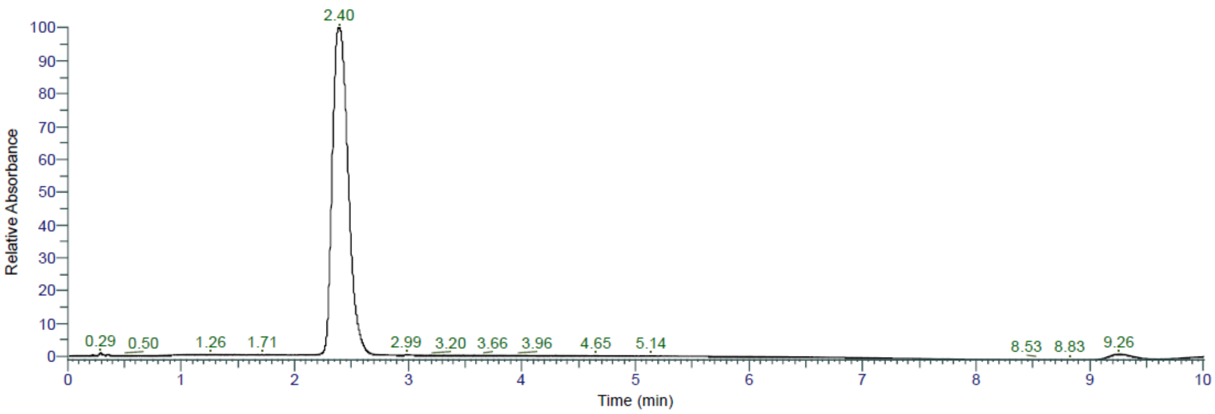
2.40
Relative Absorbance
Time (min)
0.29
0.50
1.26
1.71
2.99
3.20
3.66
3.96
4.65
5.14
8.53
8.83
9.26

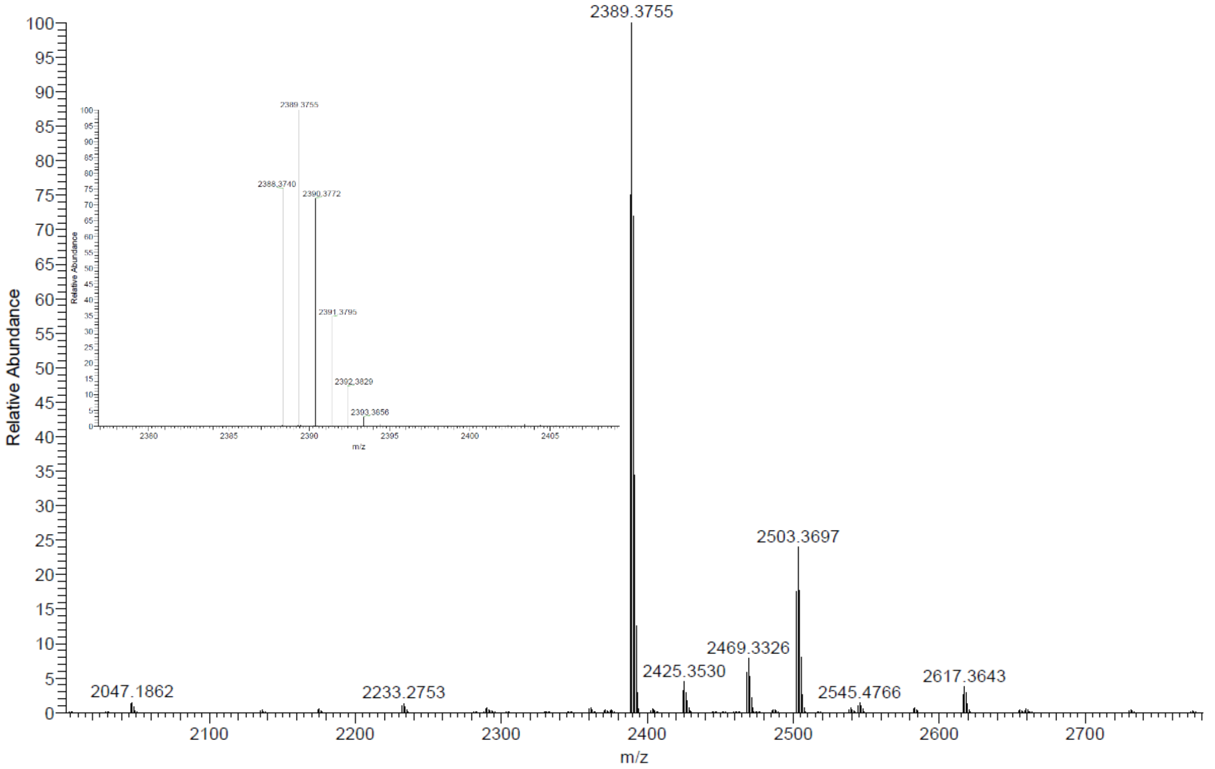
2389.3755
Relative Abundance
m/z
2047.1862
2233.2753
2425.3530
2469.3326
2503.3697
2545.4766
2617.3643

***sr10Cterm*-Onc** VDKPPYLPR*PRPPRRIYNR* (L:D 1:1)

From Rink Amide AM resin (336 mg, 0.29 mmol/g), the peptide was obtained as a white foamy solid after preparative RP-HPLC purification (4 mg, 1%).

Analytical RP-HPLC: $t_R$ = 2.42 min (A/D = 100/0 to 0/100 in 10.0 min., λ = 214 nm).

HRMS (ESI+): $C_{109}H_{178}N_{37}O_{24}$ calc./found 2389.3840/2389.3768 Da $[M + H]^+$.

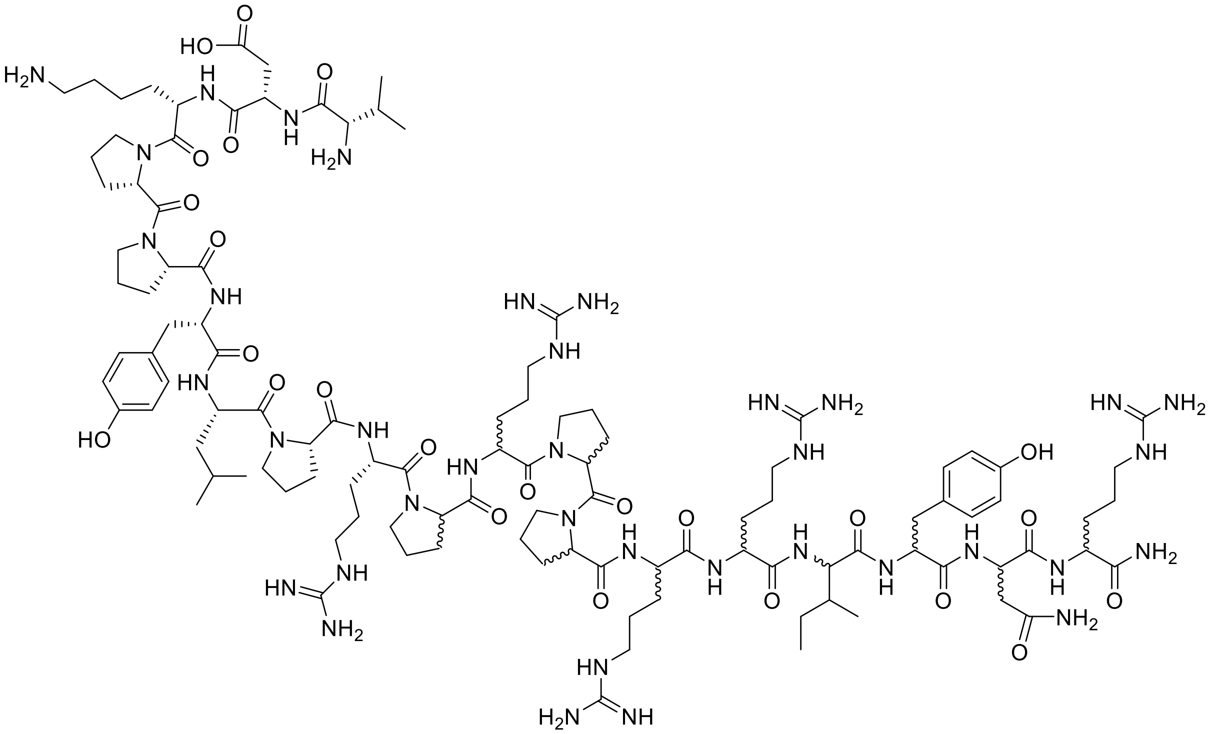

Chemical Formula: $C_{109}H_{177}N_{37}O_{24}$
Exact Mass: 2388.3767
Molecular Weight: 2389.8500

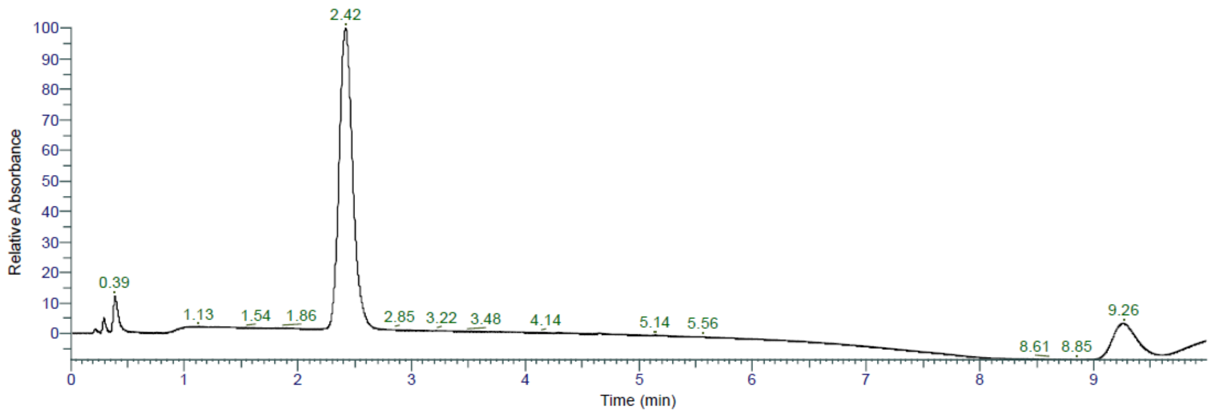
Relative Absorbance
Time (min)
2.42
0.39
1.13
1.54
1.86
2.85
3.22
3.48
4.14
5.14
5.56
8.61
8.85
9.26

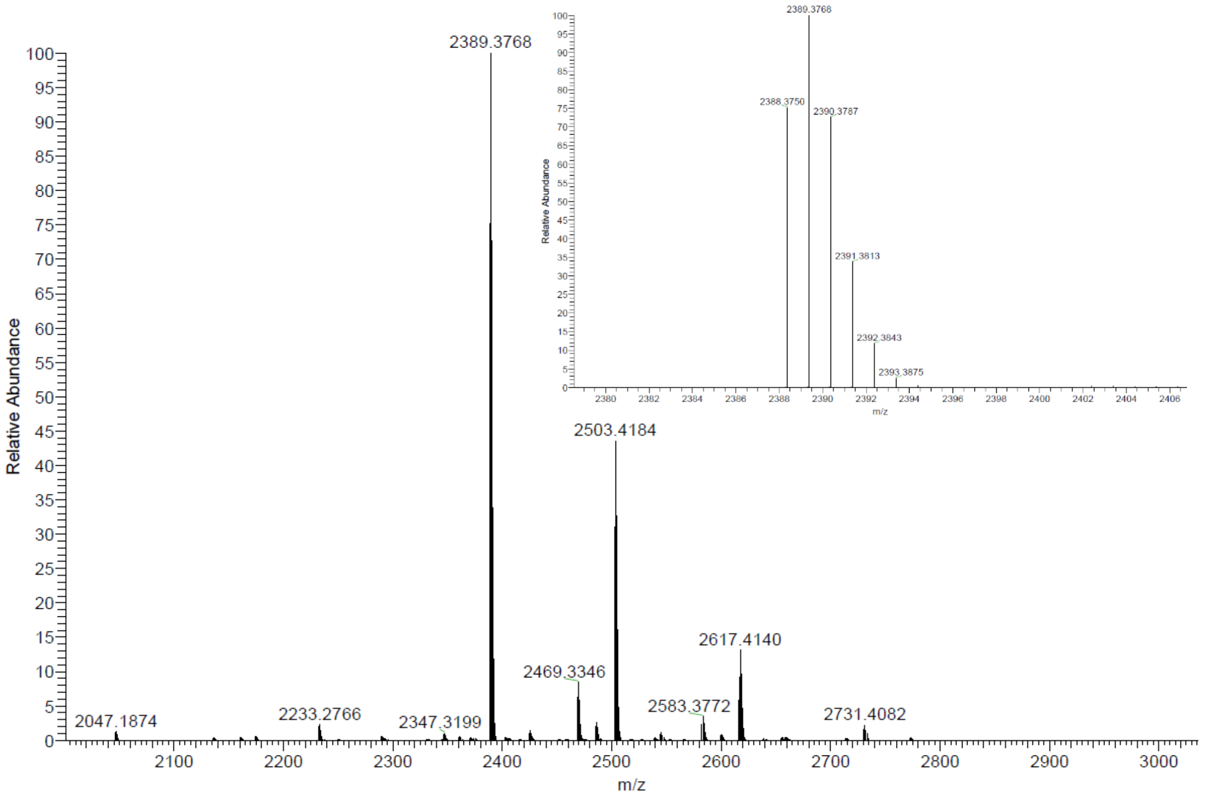
Relative Abundance
m/z
2389.3768
2503.4184
2617.4140
2469.3346
2583.3772
2731.4082
2233.2766
2347.3199
2047.1874
2388.3750
2390.3787
2391.3813
2392.3843
2393.3875

***sr11Cterm*-Onc** VDKPPYLP*RPRPPRRIYNR* (L:D 1:1)

From Rink Amide AM resin (308 mg, 0.29 mmol/g), the peptide was obtained as a white foamy solid after preparative RP-HPLC purification (118 mg, 42%).

Analytical RP-HPLC: $t_R$ = 2.38 min (A/D = 100/0 to 0/100 in 10.0 min., $\lambda$ = 214 nm).

HRMS (ESI+): $C_{109}H_{178}N_{37}O_{24}$ calc./found 2389.3840/2389.3763 Da $[M + H]^+$.

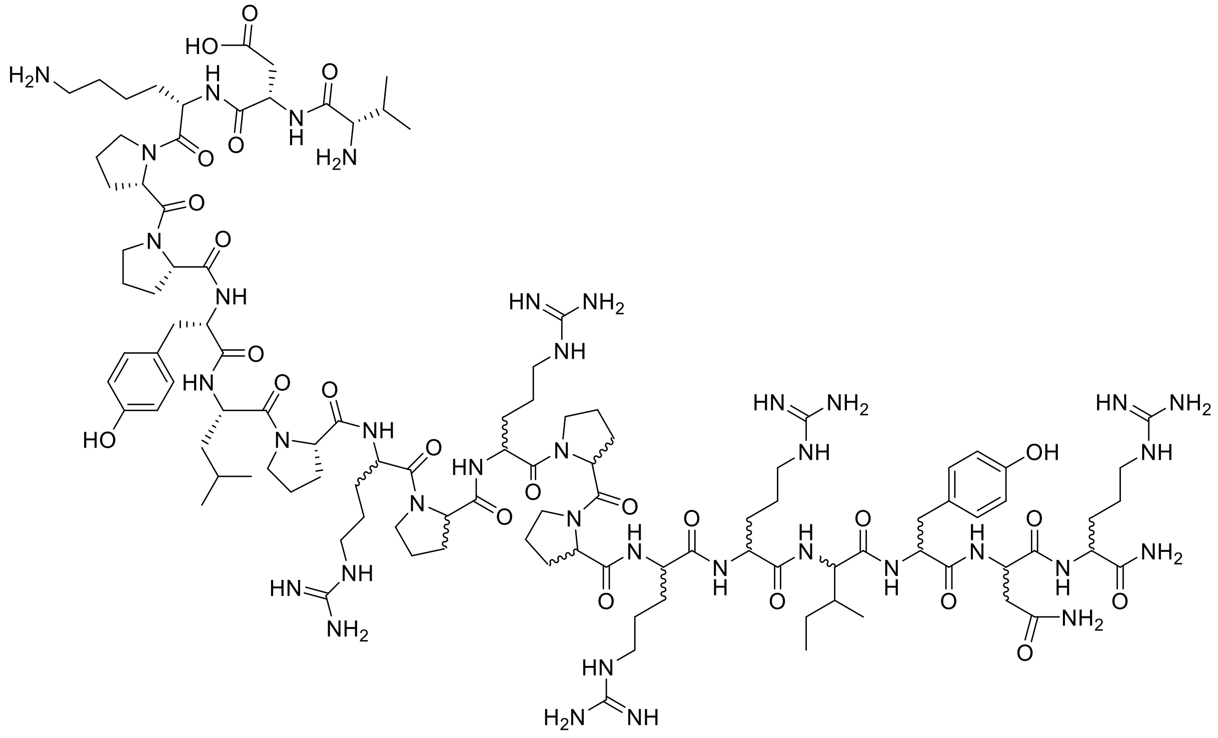

Chemical Formula: $C_{109}H_{177}N_{37}O_{24}$
Exact Mass: 2388.3767
Molecular Weight: 2389.8500

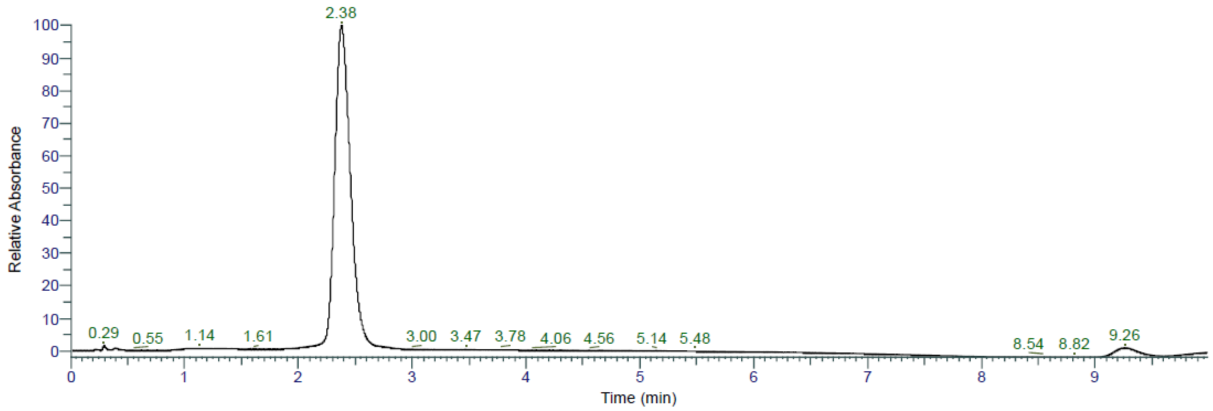
2.38
0.29
0.55
1.14
1.61
3.00
3.47
3.78
4.06
4.56
5.14
5.48
8.54
8.82
9.26
Relative Absorbance
Time (min)

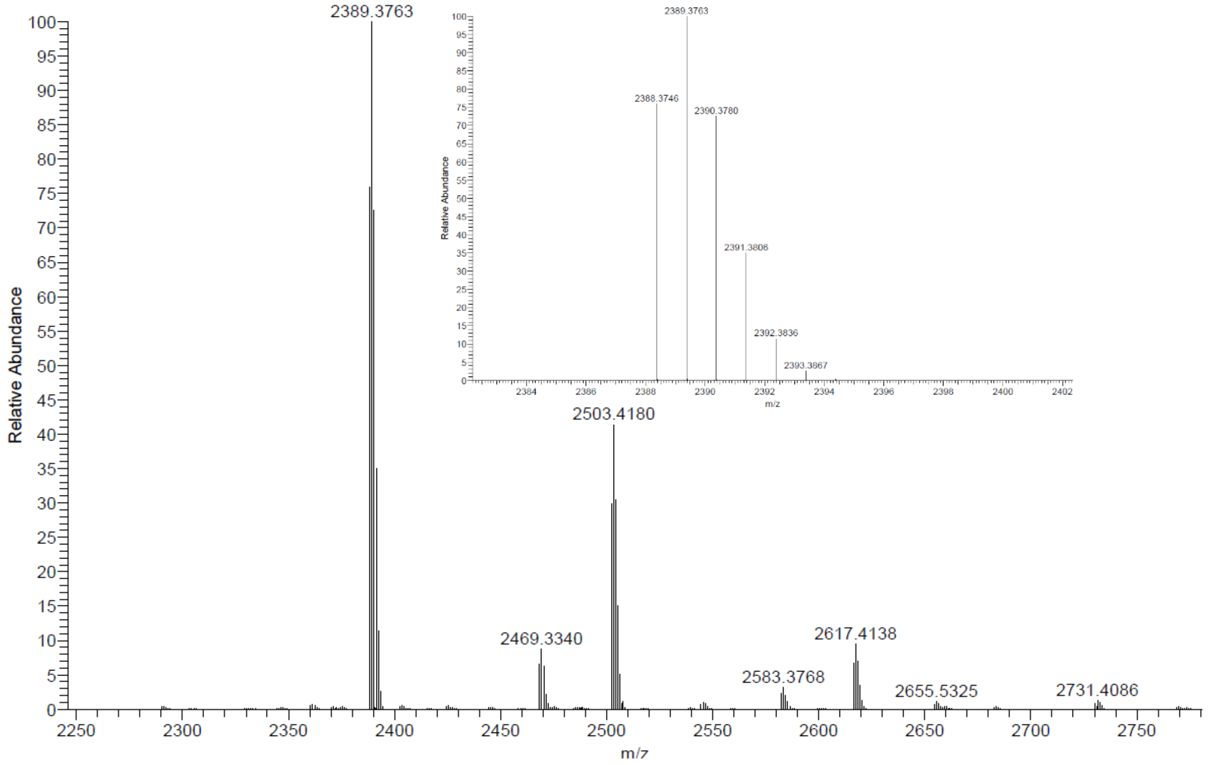
2389.3763
2503.4180
2469.3340
2617.4138
2583.3768
2655.5325
2731.4086
Relative Abundance
m/z
2388.3746
2390.3780
2391.3806
2392.3836
2393.3867

***sr12Cterm*-Onc** VDKPPYL*PRPRPPRRIYNR* (L:D 1:1)

From Rink Amide AM resin (313 mg, 0.29 mmol/g), the peptide was obtained as a white foamy solid after preparative RP-HPLC purification (85 mg, 30%).

Analytical RP-HPLC: $t_R$ = 2.44 min (A/D = 100/0 to 0/100 in 10.0 min., $\lambda$ = 214 nm).

HRMS (ESI+): $C_{109}H_{178}N_{37}O_{24}$ calc./found 2389.3840/2389.3765 Da $[M + H]^+$.

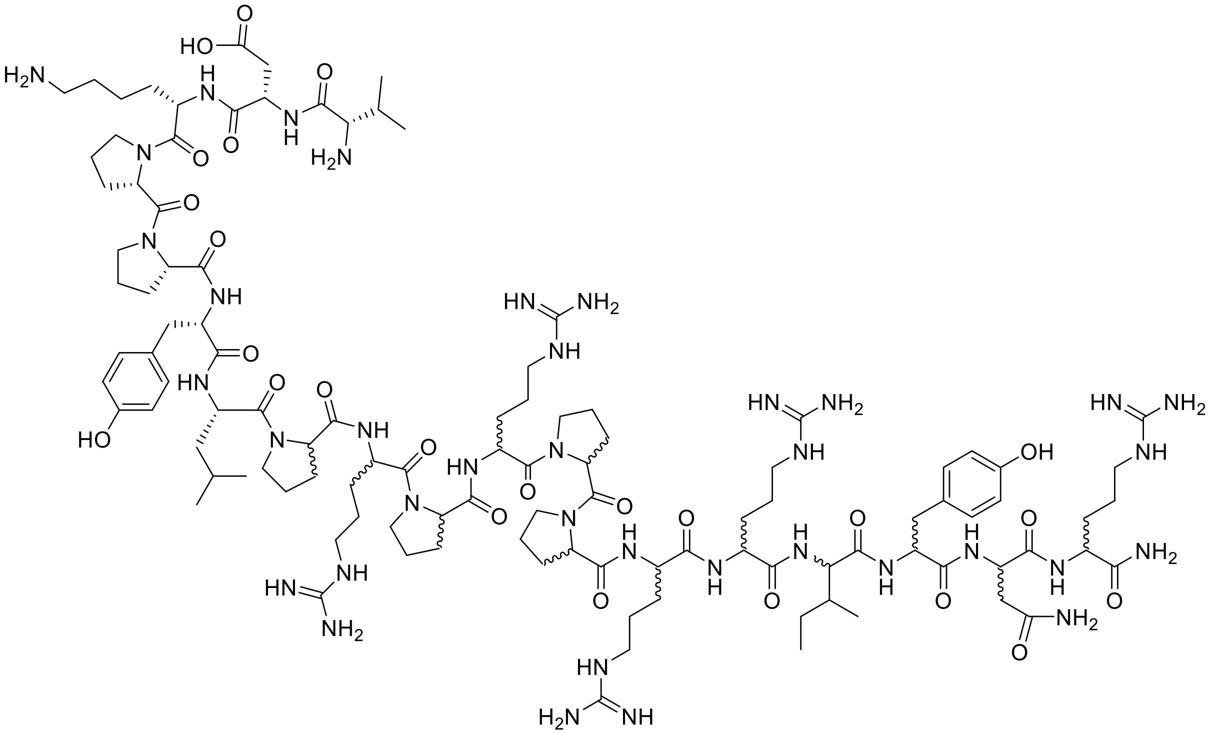

Chemical Formula: $C_{109}H_{177}N_{37}O_{24}$
Exact Mass: 2388.3767
Molecular Weight: 2389.8500

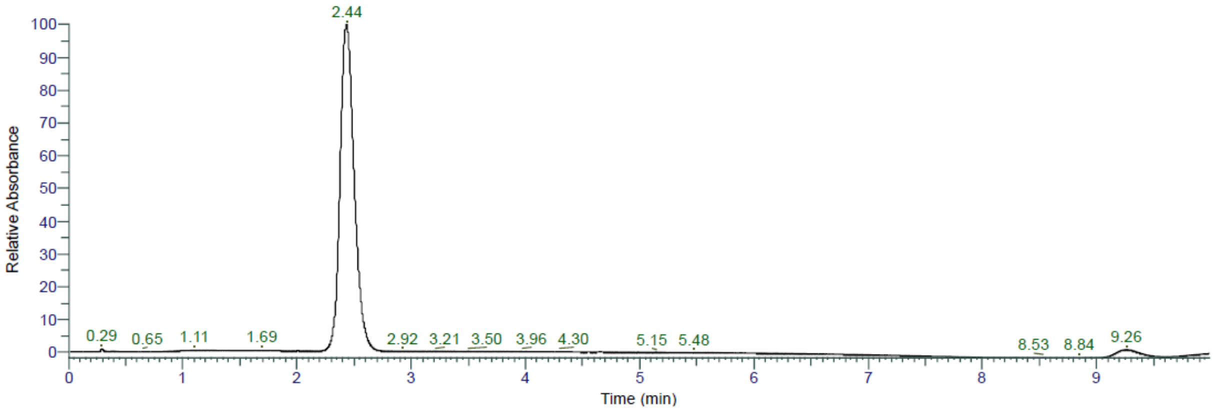
2.44
Relative Absorbance
0.29
0.65
1.11
1.69
2.92
3.21
3.50
3.96
4.30
5.15
5.48
8.53
8.84
9.26
Time (min)

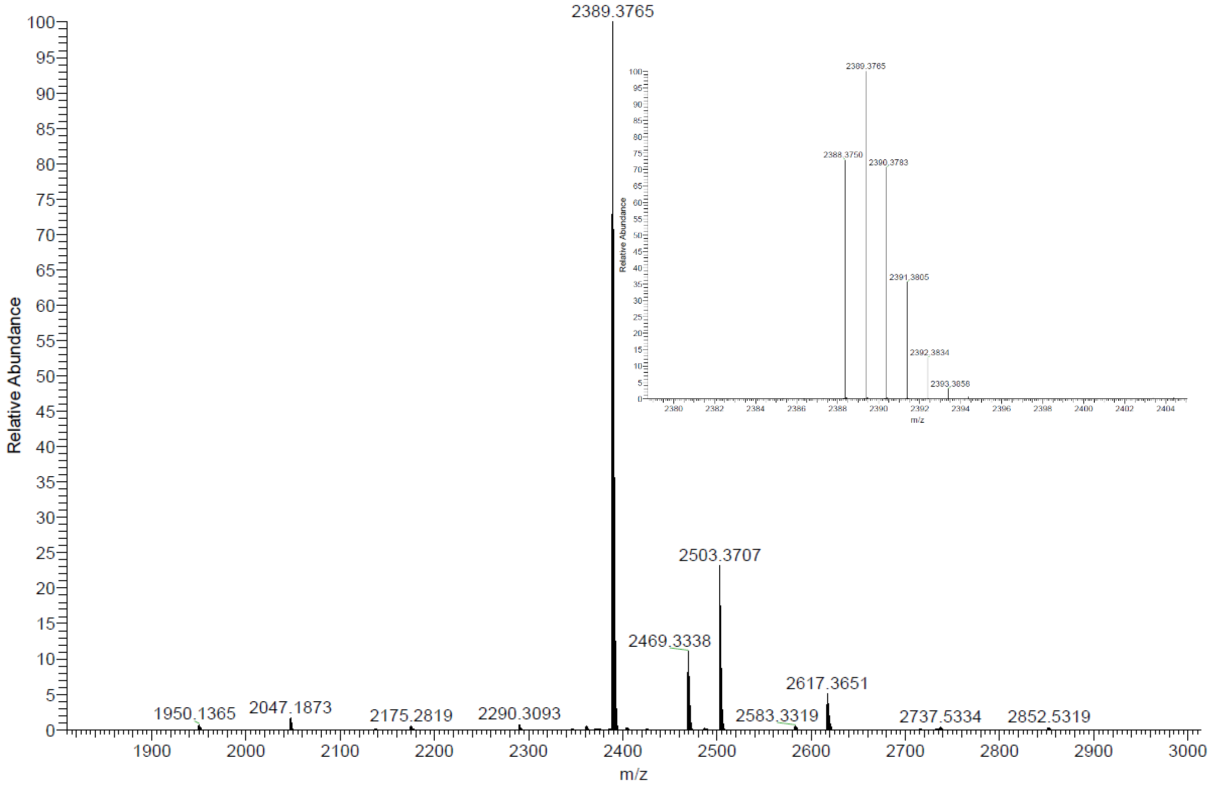
2389.3765
Relative Abundance
2503.3707
2469.3338
2617.3651
1950.1365
2047.1873
2175.2819
2290.3093
2583.3319
2737.5334
2852.5319
m/z
2389.3765
2388.3750
2390.3783
2391.3805
2392.3834
2393.3858
Relative Abundance
m/z

***srP45*-Onc** VDK*PP*YLPRPRPPRRIYNR (L:D 1:1)

From Rink Amide AM resin (310 mg, 0.29 mmol/g), the peptide was obtained as a white foamy solid after preparative RP-HPLC purification (4 mg, 2%).

Analytical RP-HPLC: $t_R$ = 2.27 min (A/D = 100/0 to 0/100 in 10.0 min., λ = 214 nm).

HRMS (ESI+): $C_{109}H_{178}N_{37}O_{24}$ calc./found 2389.3840/2389.3778 Da $[M + H]^+$.

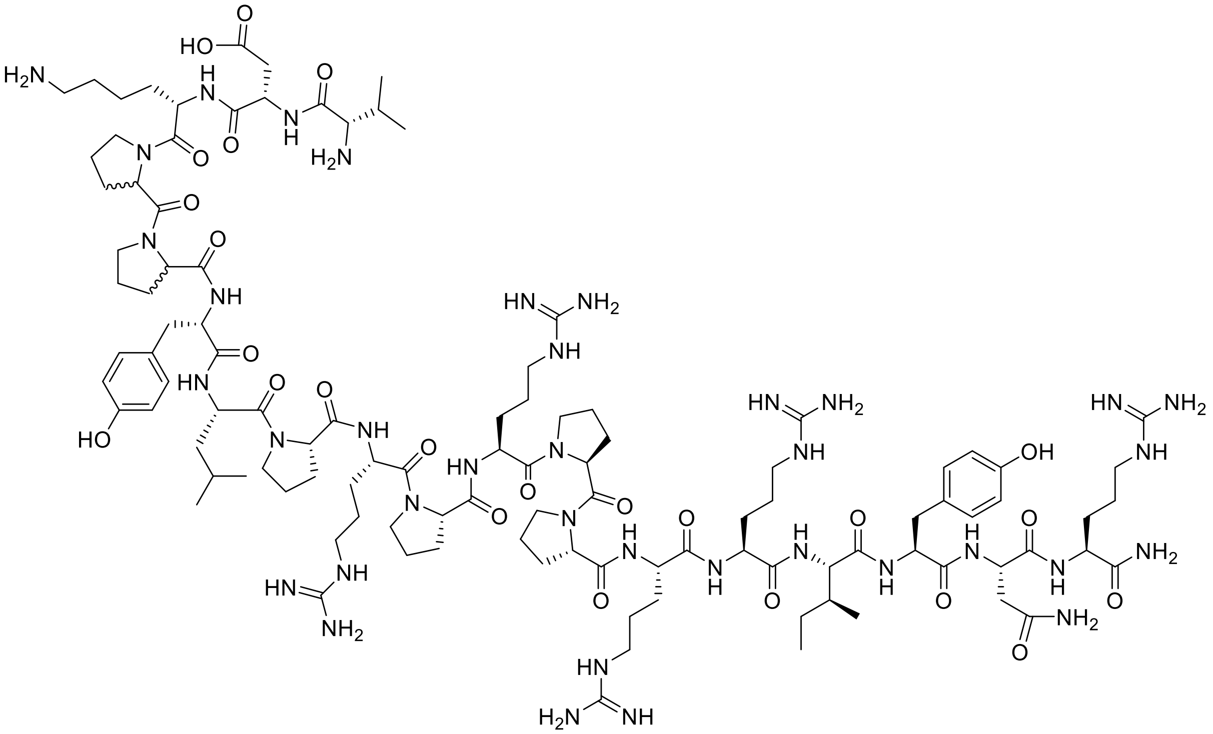

Chemical Formula: $C_{109}H_{177}N_{37}O_{24}$
Exact Mass: 2388.3767
Molecular Weight: 2389.8500

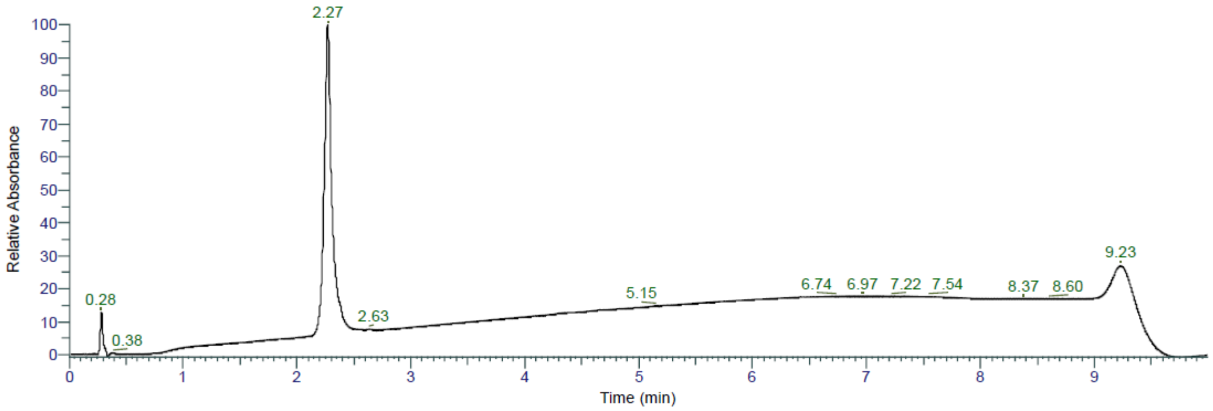

Relative Absorbance
Time (min)
2.27
0.28
0.38
2.63
5.15
6.74
6.97
7.22
7.54
8.37
8.60
9.23


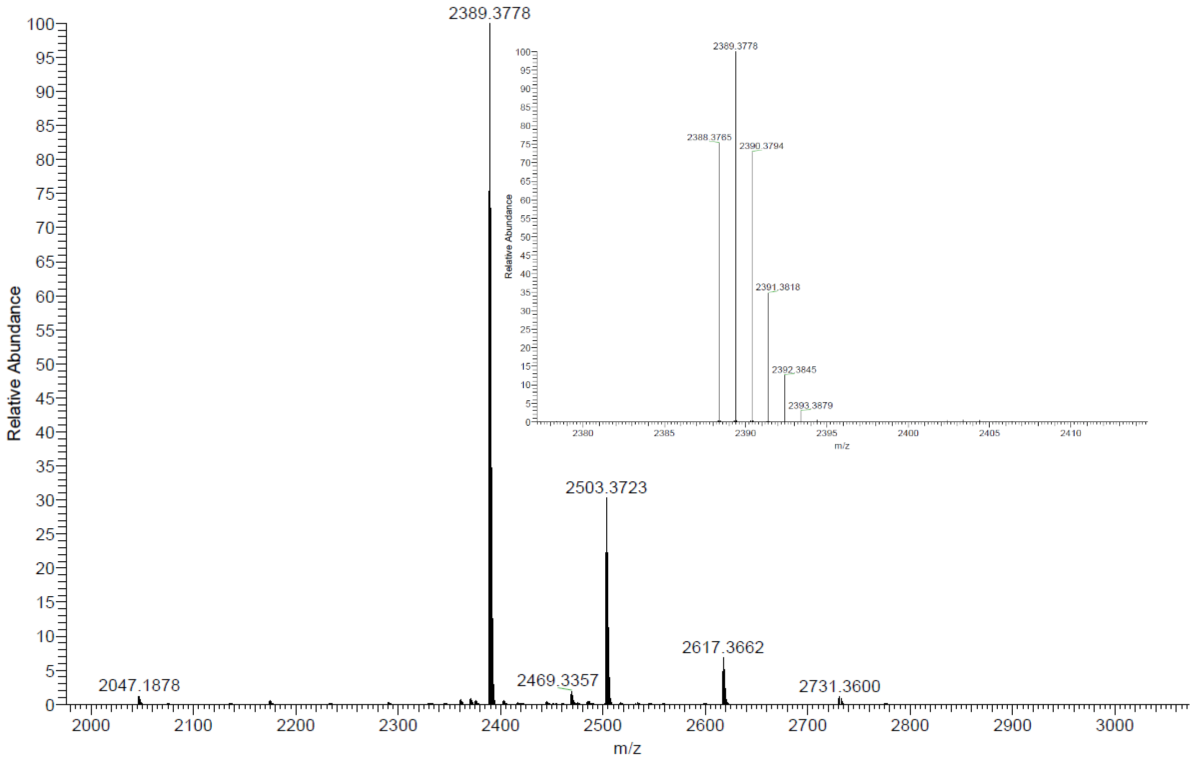

Relative Abundance
m/z
2389.3778
2503.3723
2617.3662
2469.3357
2047.1878
2731.3600
2388.3765
2390.3794
2391.3818
2392.3845
2393.3879

***sr3Nterm*-Onc** *VDK*PPYLPRPRPPRRIYNR (L:D 1:1)

From Rink Amide AM resin (342 mg, 0.29 mmol/g), the peptide was obtained as a white foamy solid after preparative RP-HPLC purification (94 mg, 30%).

Analytical RP-HPLC: $t_R$ = 2.41 min (A/D = 100/0 to 0/100 in 10.0 min., λ = 214 nm).

HRMS (ESI+): $C_{109}H_{178}N_{37}O_{24}$ calc./found 2389.3840/2389.3860 Da $[M + H]^+$.

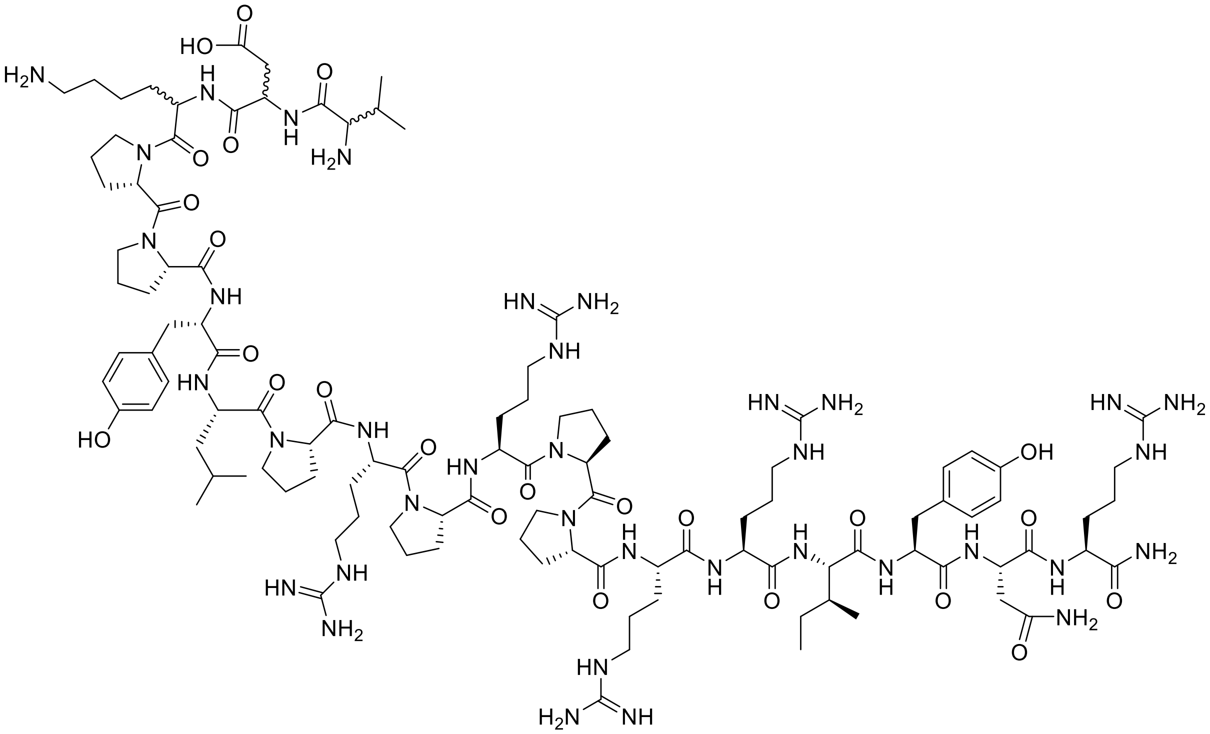

Chemical Formula: $C_{109}H_{177}N_{37}O_{24}$
Exact Mass: 2388.3767
Molecular Weight: 2389.8500

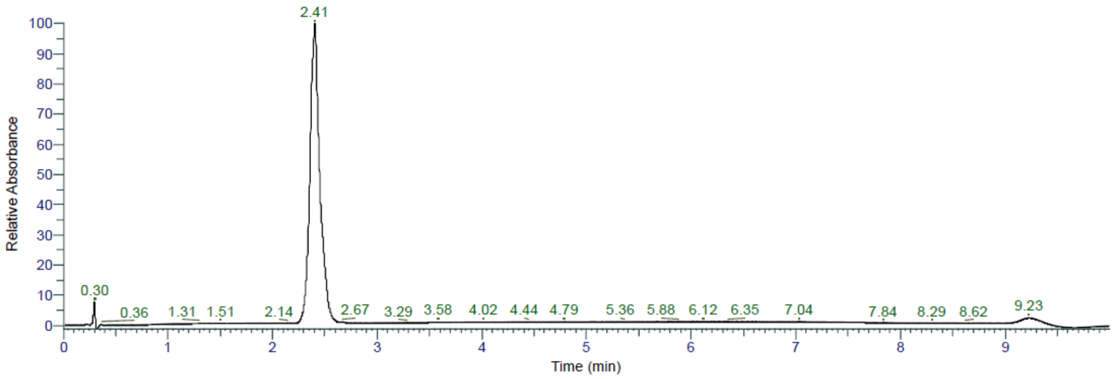
2.41
0.30
0.36
1.31
1.51
2.14
2.67
3.29
3.58
4.02
4.44
4.79
5.36
5.88
6.12
6.35
7.04
7.84
8.29
8.62
9.23
Relative Absorbance
Time (min)

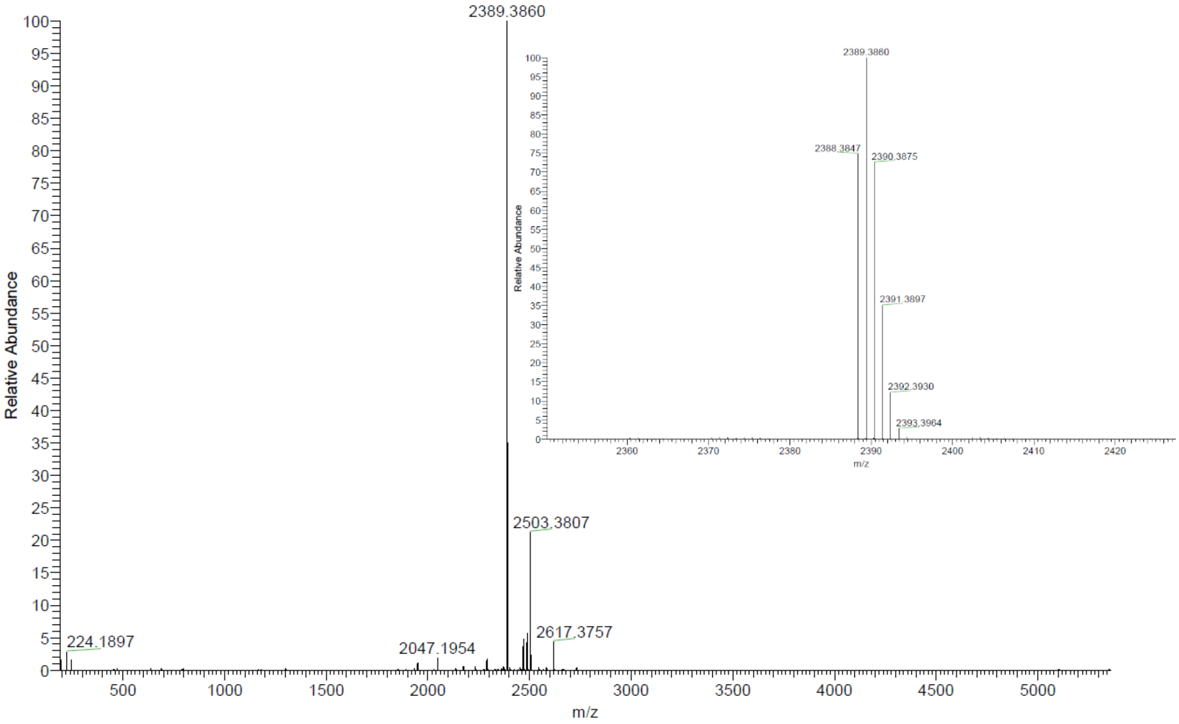
2389.3860
2503.3807
2617.3757
2047.1954
224.1897
Relative Abundance
m/z
2388.3847
2390.3875
2391.3897
2392.3930
2393.3964

***sr3N5Cterm*****-Onc** *VDK*PPYLPRPRPPR*RIYNR* (L:D 1:1)

From Rink Amide AM resin (348 mg, 0.29 mmol/g), the peptide was obtained as a white foamy solid after preparative RP-HPLC purification (113 mg, 35%).

Analytical RP-HPLC: $t_R$ = 2.44 min (A/D = 100/0 to 0/100 in 10.0 min., λ = 214 nm).

HRMS (ESI+): $C_{109}H_{178}N_{37}O_{24}$ calc./found 2389.3840/2389.3869 Da $[M + H]^+$.

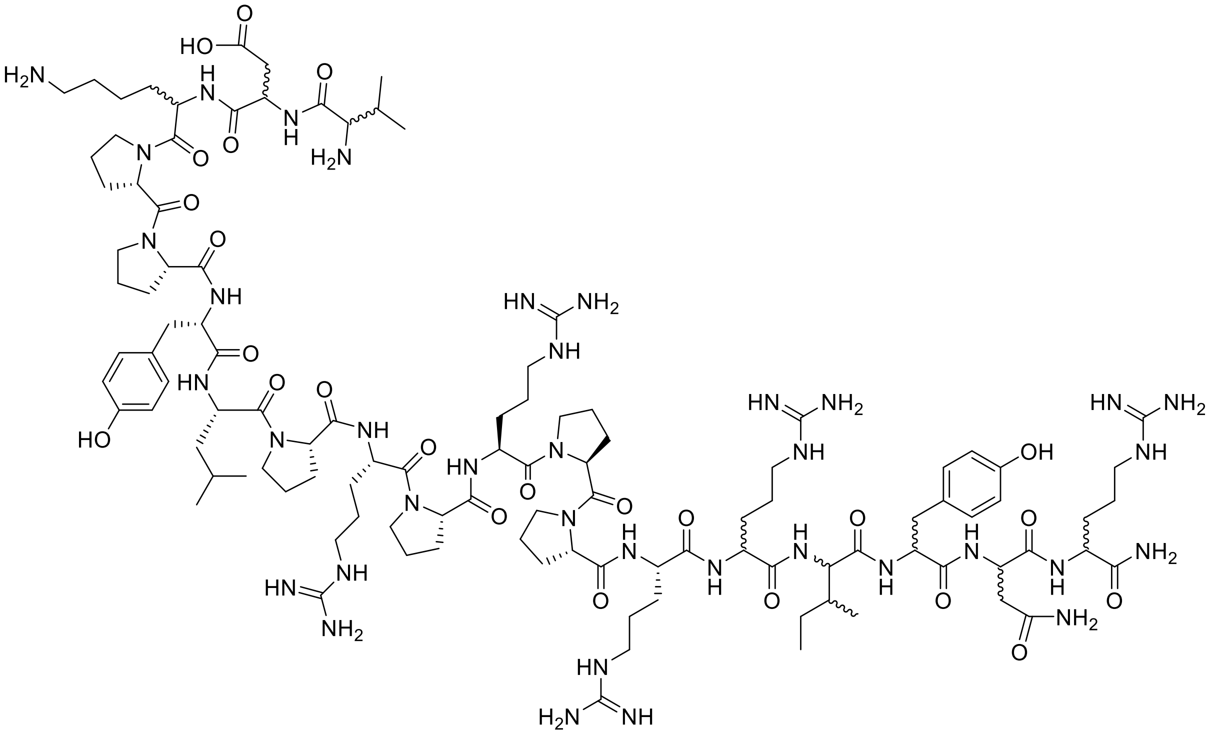

Chemical Formula: $C_{109}H_{177}N_{37}O_{24}$
Exact Mass: 2388.3767
Molecular Weight: 2389.8500

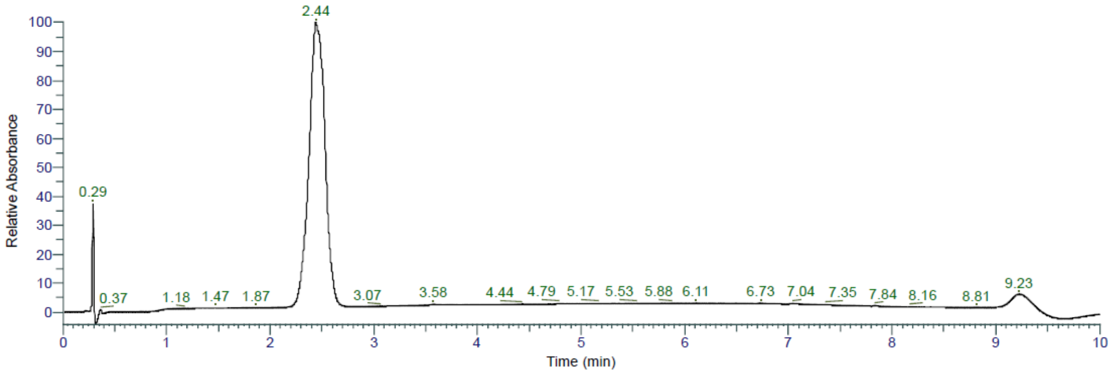

Relative Absorbance
Time (min)
2.44
0.29
0.37
1.18
1.47
1.87
3.07
3.58
4.44
4.79
5.17
5.53
5.88
6.11
6.73
7.04
7.35
7.84
8.16
8.81
9.23


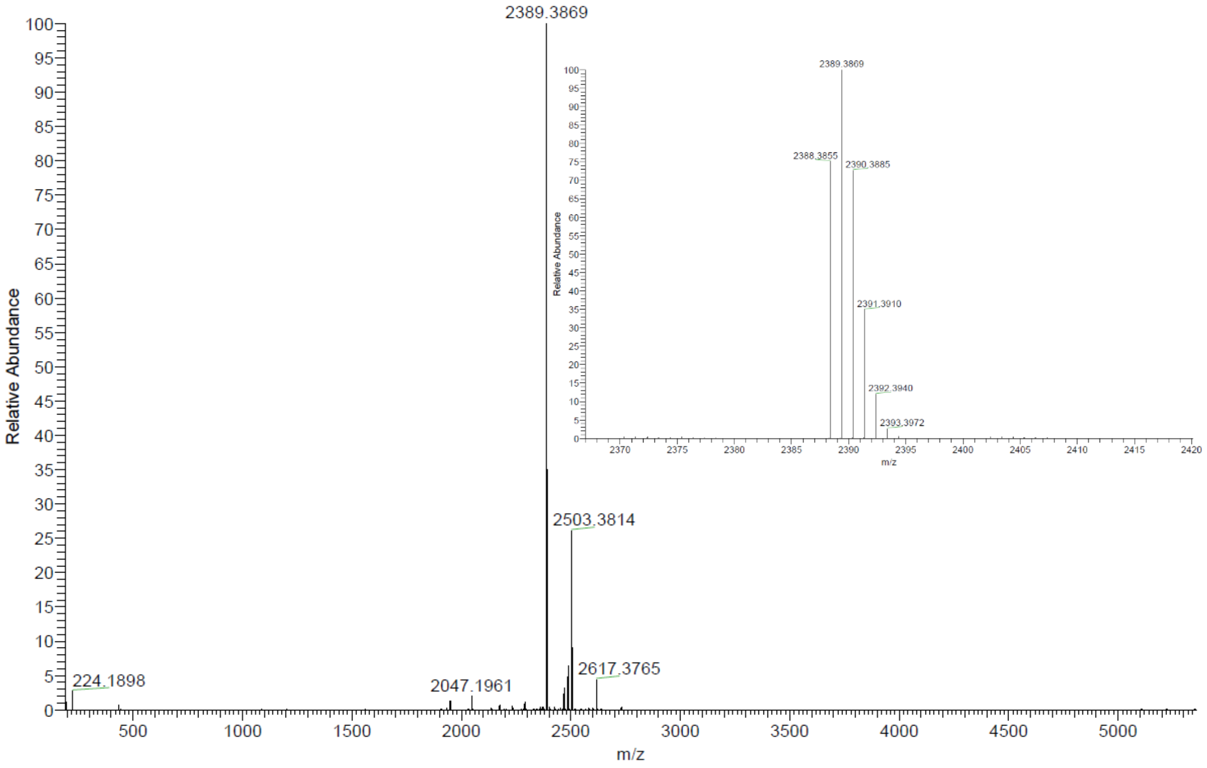

Relative Abundance
m/z
2389.3869
2503.3814
2617.3765
2047.1961
224.1898
2388.3855
2390.3885
2391.3910
2392.3940
2393.3972

***sr14Nterm*-Onc** *VDKPPYLPRPRPPR*RIYNR (L:D 1:1)

From Rink Amide AM resin (368 mg, 0.29 mmol/g), the peptide was obtained as a white foamy solid after preparative RP-HPLC purification (102 mg, 30%).

Analytical RP-HPLC: $t_R$ = 2.43 min (A/D = 100/0 to 0/100 in 10.0 min., $\lambda$ = 214 nm).

HRMS (ESI+): $C_{109}H_{178}N_{37}O_{24}$ calc./found 2389.3840/2389.3856 Da $[M + H]^+$.

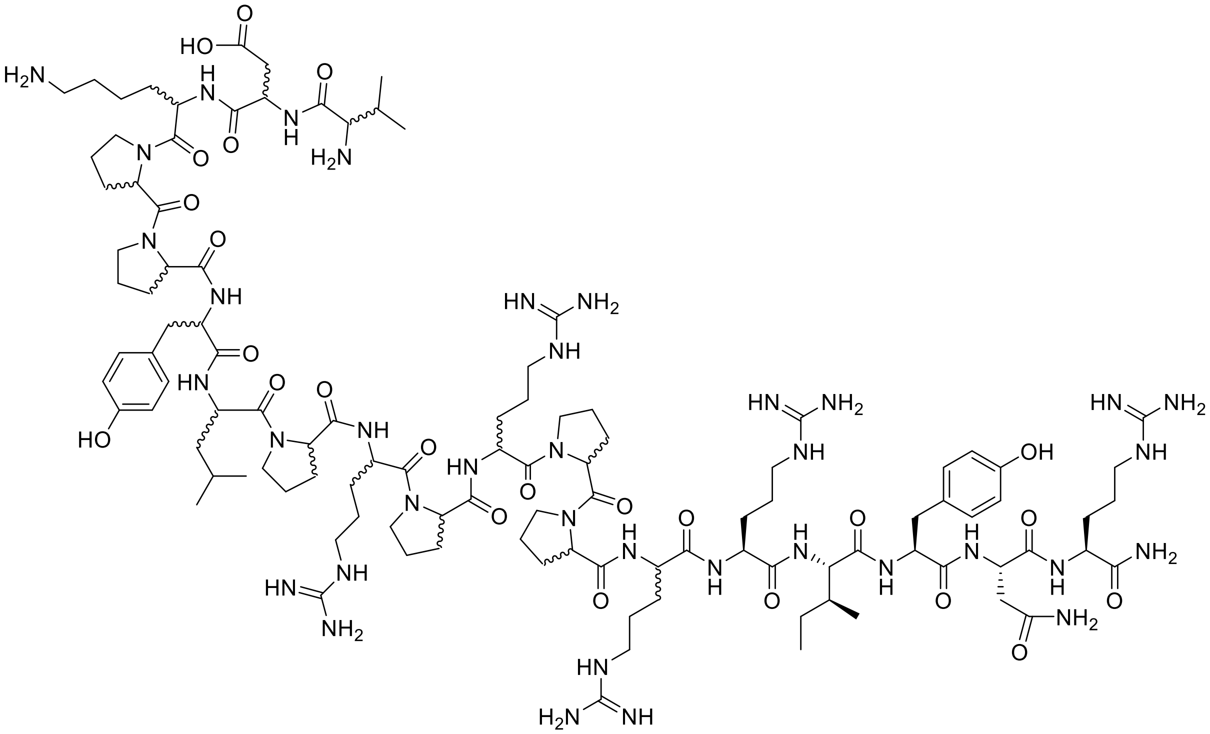

Chemical Formula: $C_{109}H_{177}N_{37}O_{24}$
Exact Mass: 2388.3767
Molecular Weight: 2389.8500

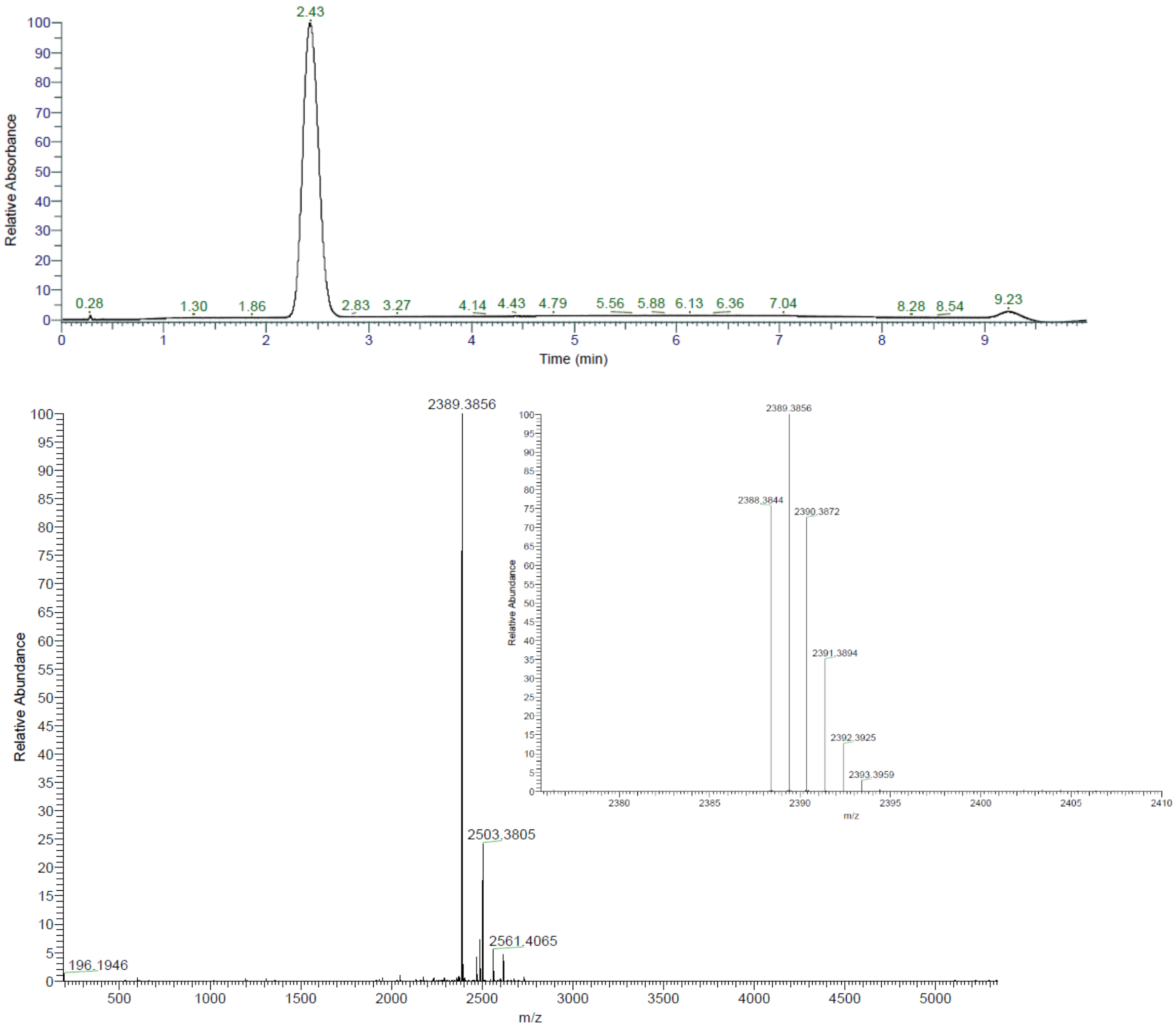
2.43
0.28
1.30
1.86
2.83
3.27
4.14
4.43
4.79
5.56
5.88
6.13
6.36
7.04
8.28
8.54
9.23
Relative Absorbance
Time (min)
2389.3856
2503.3805
2561.4065
196.1946
Relative Abundance
m/z
2388.3844
2390.3872
2391.3894
2392.3925
2393.3959

***sr*-Onc** *VDKPPYLPRPRPPRRIYNR* (L:D 1:1)

From Rink Amide AM resin (331 mg, 0.29 mmol/g), the peptide was obtained as a white foamy solid after preparative RP-HPLC purification (100 mg, 33%).

Analytical RP-HPLC: $t_R$ = 2.49 min (A/D = 100/0 to 0/100 in 10.0 min., λ = 214 nm).

HRMS (ESI+): $C_{109}H_{178}N_{37}O_{24}$ calc./found 2389.3840/2389.4110 Da $[M + H]^+$.

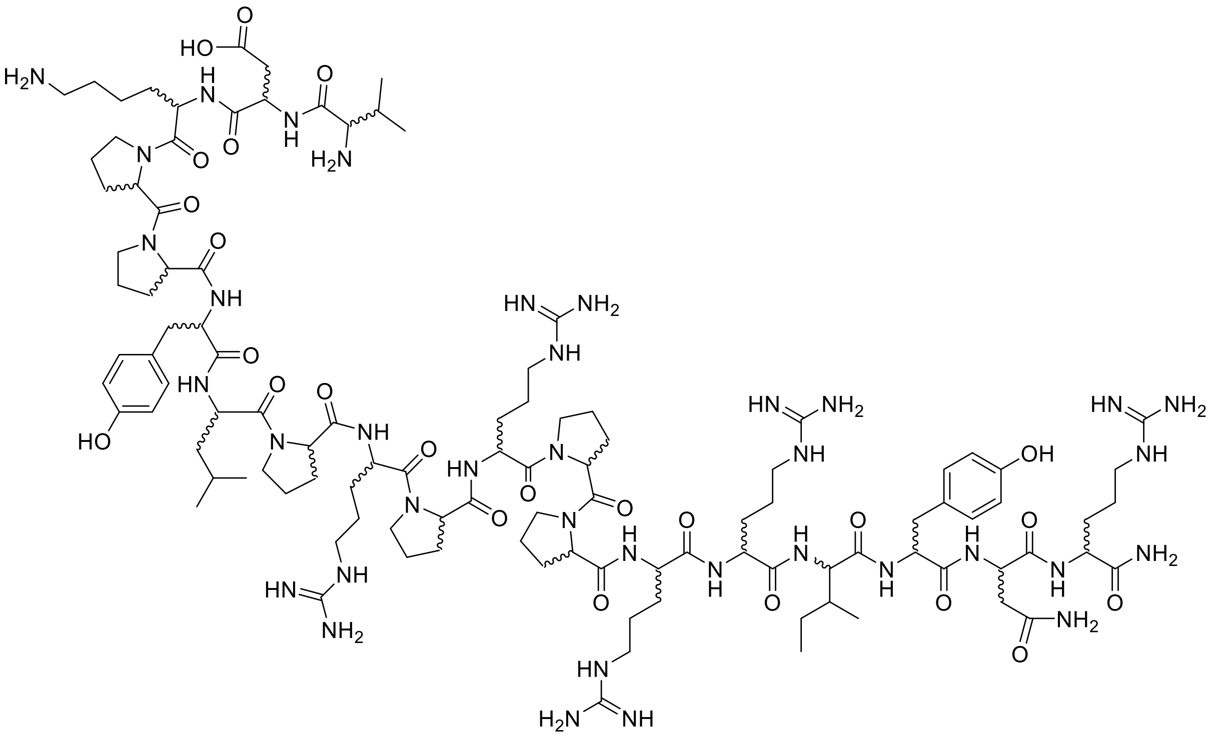

Chemical Formula: $C_{109}H_{177}N_{37}O_{24}$
Exact Mass: 2388.3767
Molecular Weight: 2389.8500

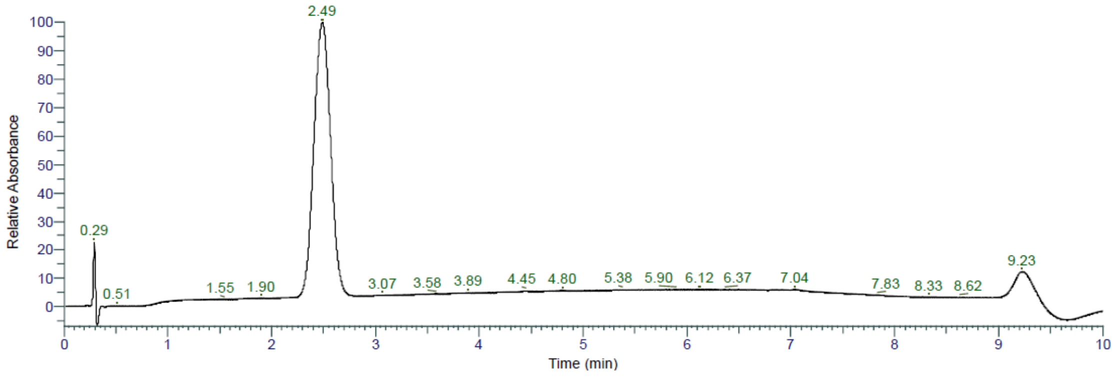
Relative Absorbance
Time (min)
2.49
0.29
0.51
1.55
1.90
3.07
3.58
3.89
4.45
4.80
5.38
5.90
6.12
6.37
7.04
7.83
8.33
8.62
9.23

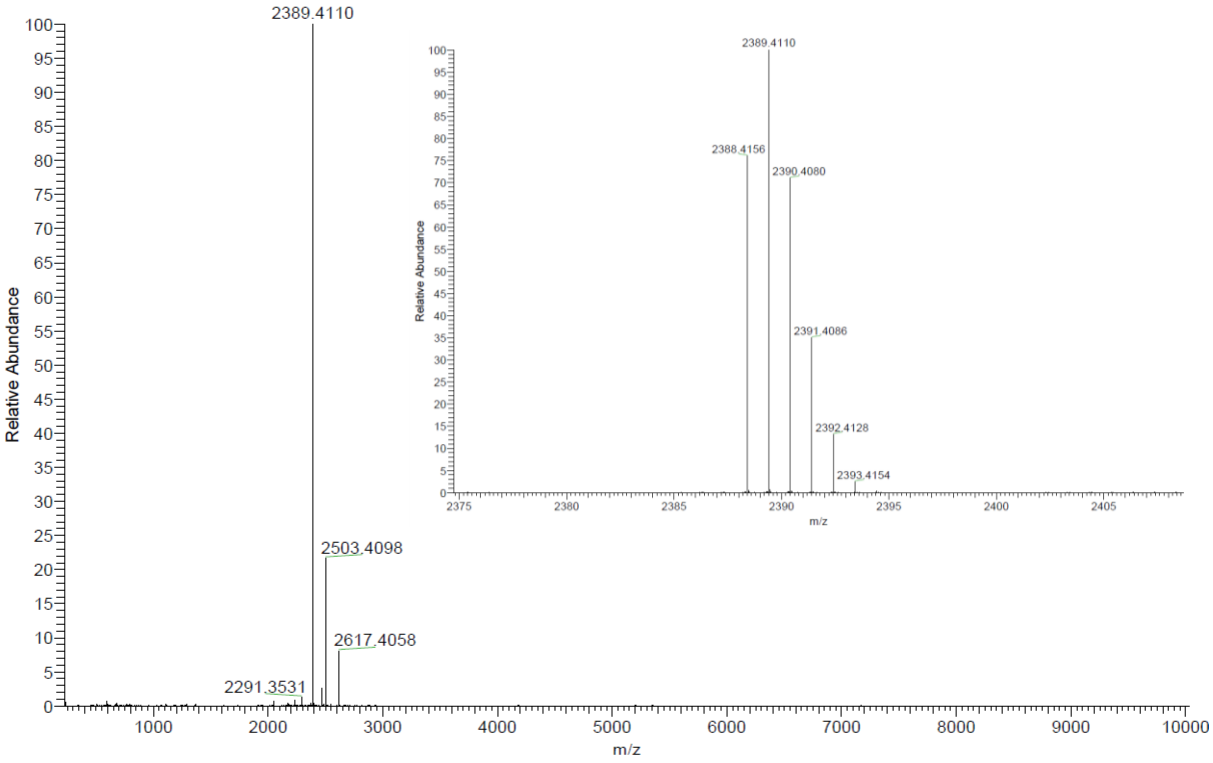
Relative Abundance
m/z
2389.4110
2503.4098
2617.4058
2291.3531
2388.4156
2390.4080
2391.4086
2392.4128
2393.4154

**D5Cterm-Onc** VDKPPYLPRPRPPRriynr

From Rink Amide AM resin (298 mg, 0.29 mmol/g), the peptide was obtained as a white foamy solid after preparative RP-HPLC purification (84 mg, 31%).

Analytical RP-HPLC: $t_R$ = 2.39 min (A/D = 100/0 to 0/100 in 10.0 min., λ = 214 nm).

HRMS (ESI+): $C_{109}H_{178}N_{37}O_{24}$ calc./found 2389.3840/2389.3763 Da $[M + H]^+$.

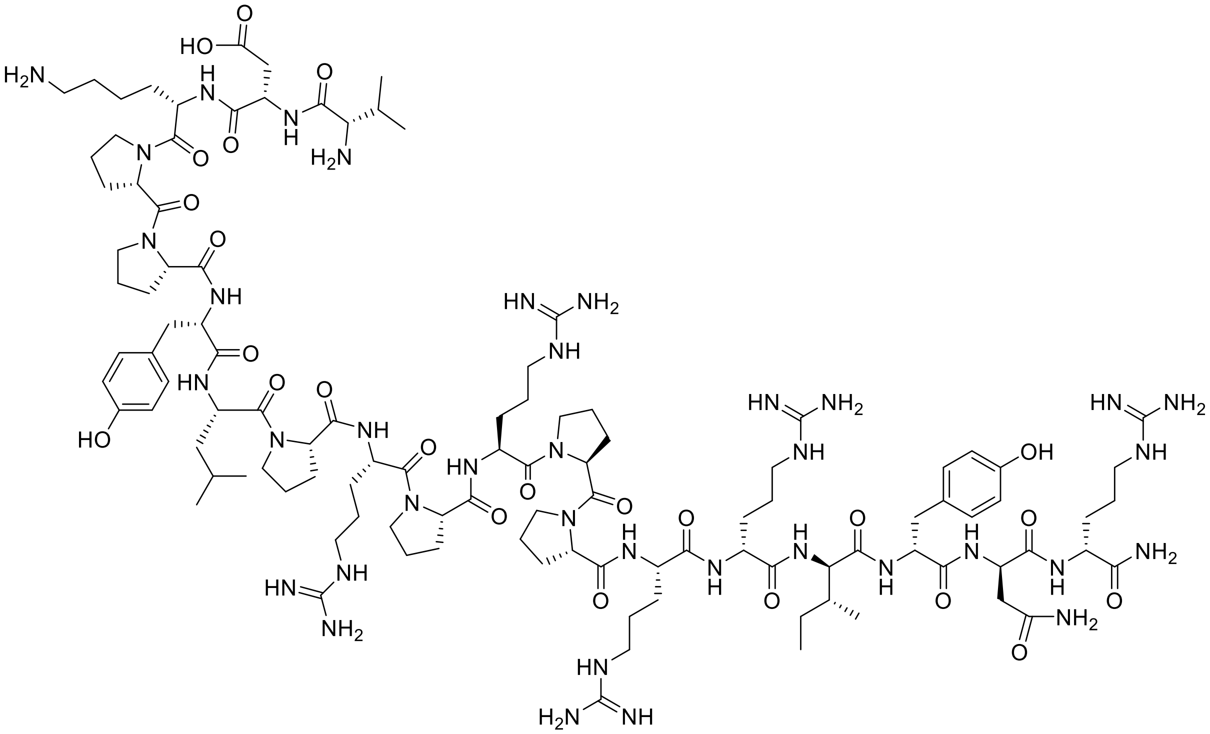

Chemical Formula: $C_{109}H_{177}N_{37}O_{24}$
Exact Mass: 2388.3767
Molecular Weight: 2389.8500

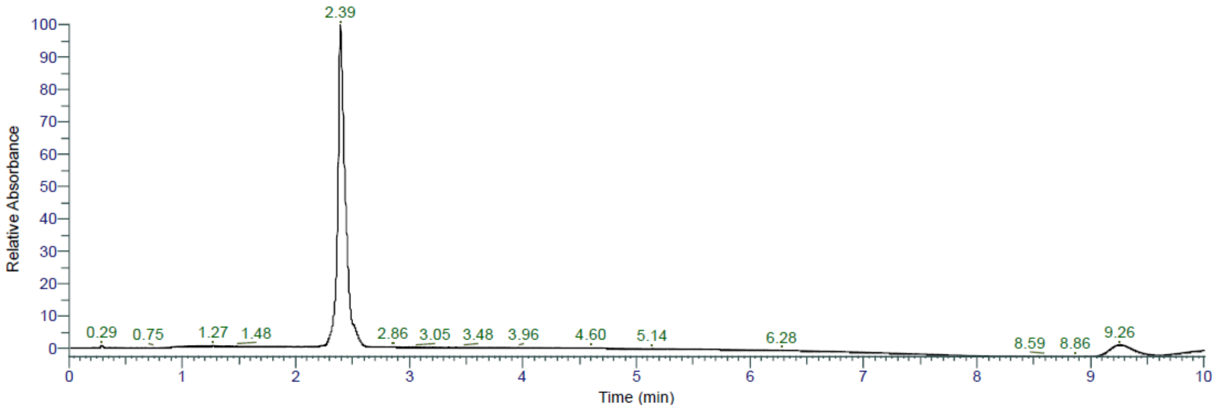
2.39
Relative Absorbance
Time (min)
0.29
0.75
1.27
1.48
2.86
3.05
3.48
3.96
4.60
5.14
6.28
8.59
8.86
9.26

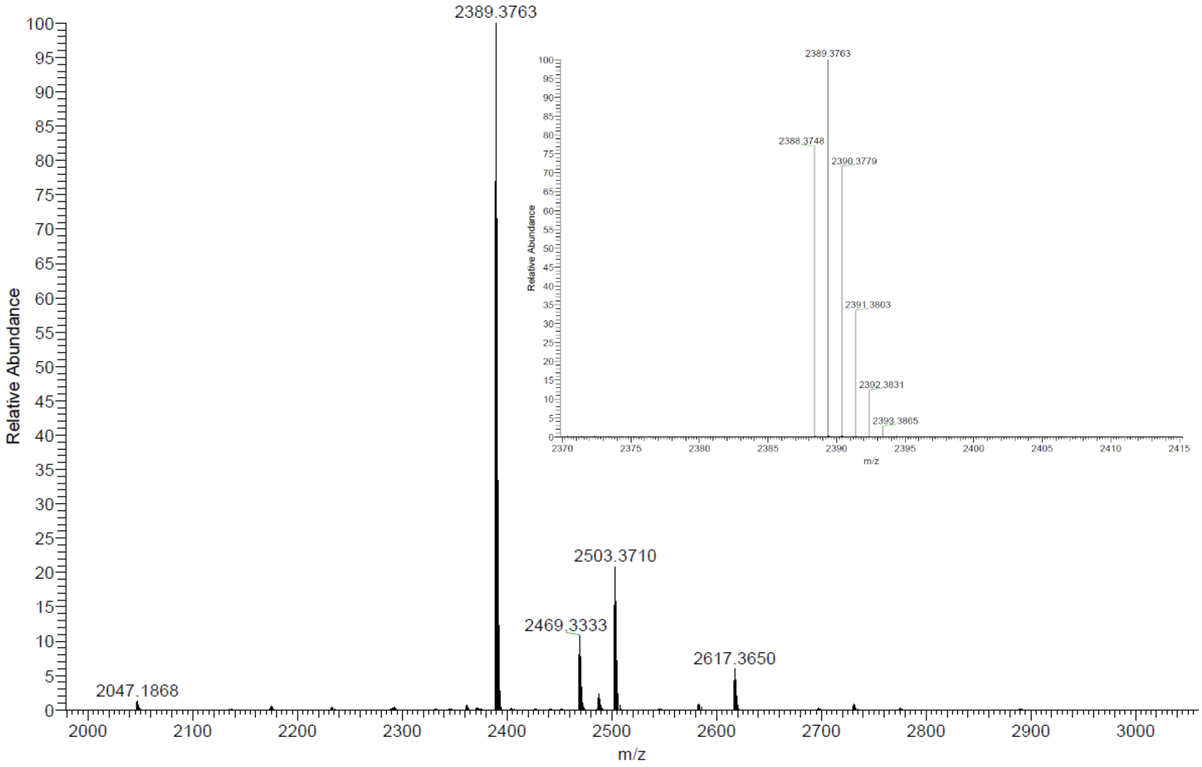
2389.3763
Relative Abundance
m/z
2047.1868
2469.3333
2503.3710
2617.3650
2388.3748
2390.3779
2391.3803
2392.3831
2393.3865

**D8Cterm-Onc** VDKPPYLPRPRpprriynr

From Rink Amide AM resin (300 mg, 0.25 mmol/g), the peptide was obtained as a white foamy solid after preparative RP-HPLC purification (7.4 mg, 3%).

Analytical RP-HPLC: $t_R$ = 2.29 min (A/D = 100/0 to 0/100 in 10.0 min., $\lambda$ = 214 nm).

HRMS (ESI+): $C_{109}H_{178}N_{37}O_{24}$ calc./found 2389.3840/2389.3817 Da $[M + H]^+$.

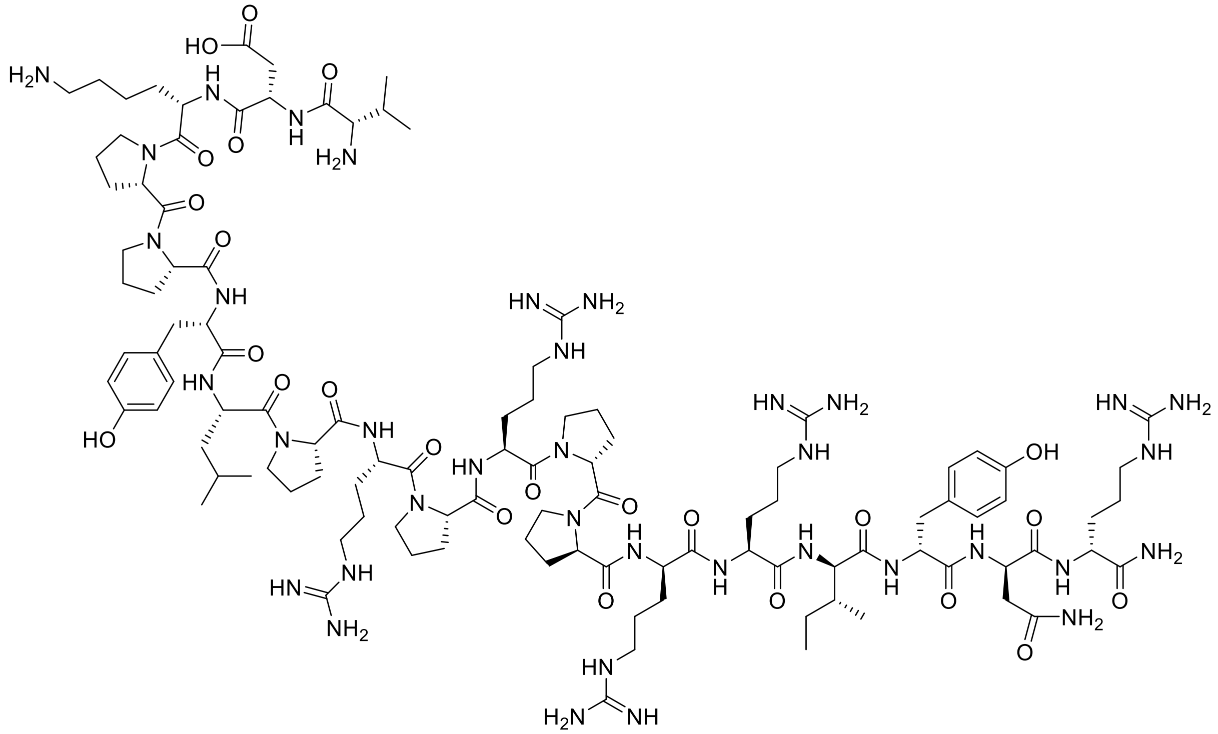

Chemical Formula: $C_{109}H_{177}N_{37}O_{24}$
Exact Mass: 2388.3767
Molecular Weight: 2389.8500

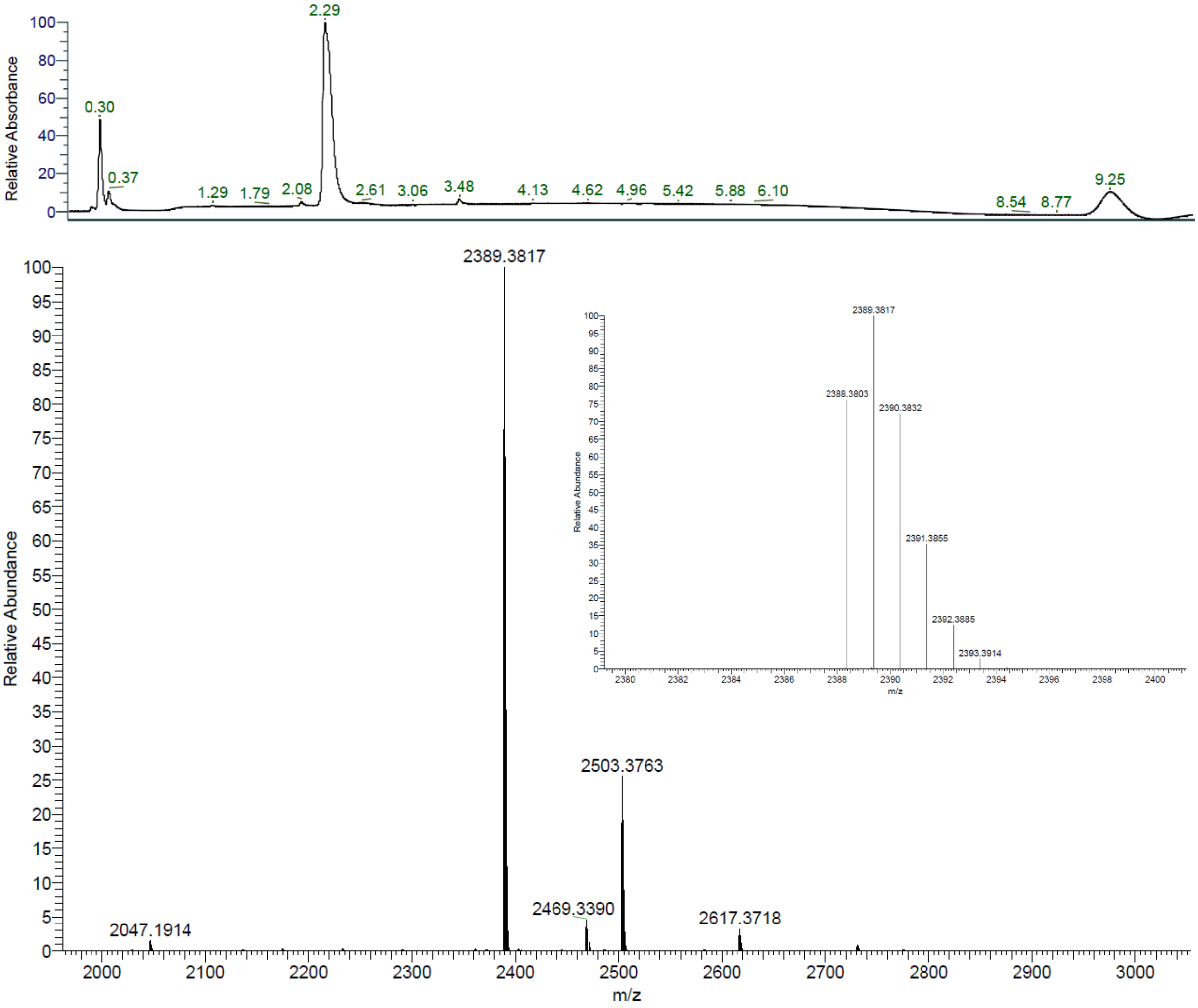
Relative Absorbance
100
80
60
40
20
0
0.30
0.37
1.29
1.79
2.08
2.29
2.61
3.06
3.48
4.13
4.62
4.96
5.42
5.88
6.10
8.54
8.77
9.25
Relative Abundance
2389.3817
2503.3763
2469.3390
2617.3718
2047.1914
m/z
2388.3803
2390.3832
2391.3855
2392.3885
2393.3914

**D9Cterm-Onc** VDKPPYLPRPrpprriynr

From Rink Amide AM resin (300 mg, 0.25 mmol/g), the peptide was obtained as a white foamy solid after preparative RP-HPLC purification (7.3 mg, 3%).

Analytical RP-HPLC: $t_R$ = 2.31 min (A/D = 100/0 to 0/100 in 10.0 min., $\lambda$ = 214 nm).

HRMS (ESI+): $C_{109}H_{178}N_{37}O_{24}$ calc./found 2389.3840/2389.3815 Da $[M + H]^+$.

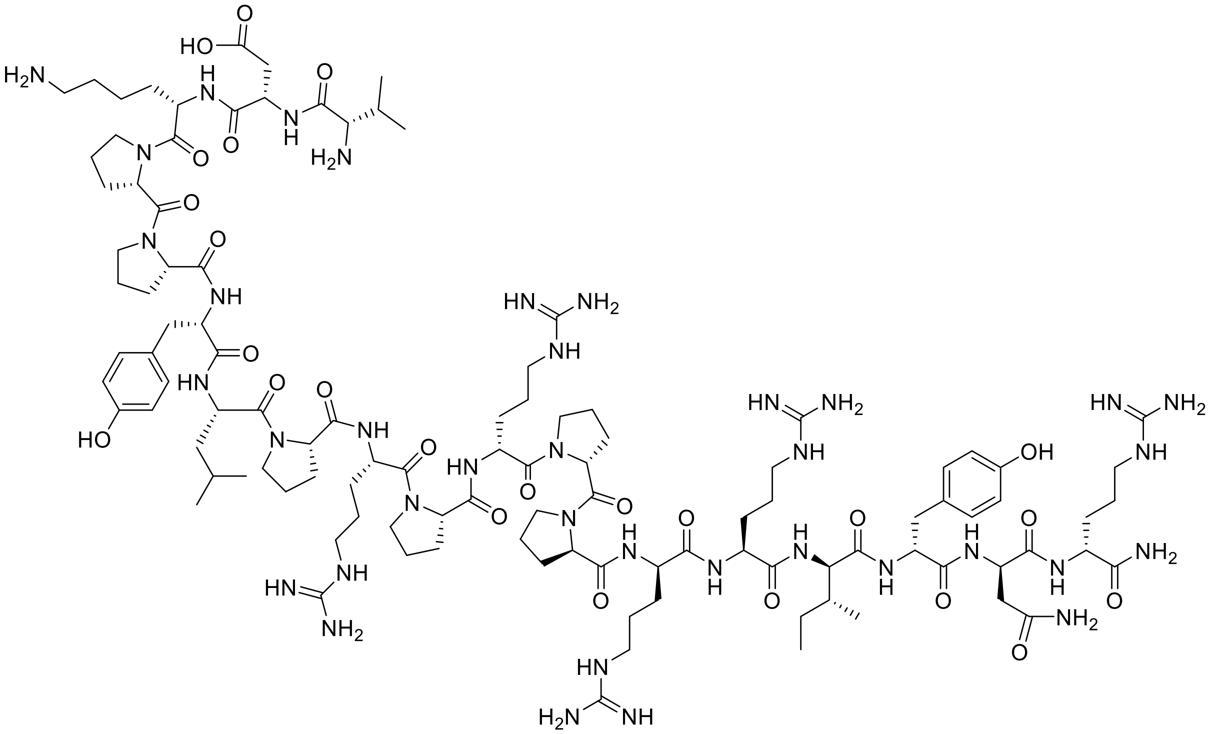

Chemical Formula: $C_{109}H_{177}N_{37}O_{24}$
Exact Mass: 2388.3767
Molecular Weight: 2389.8500

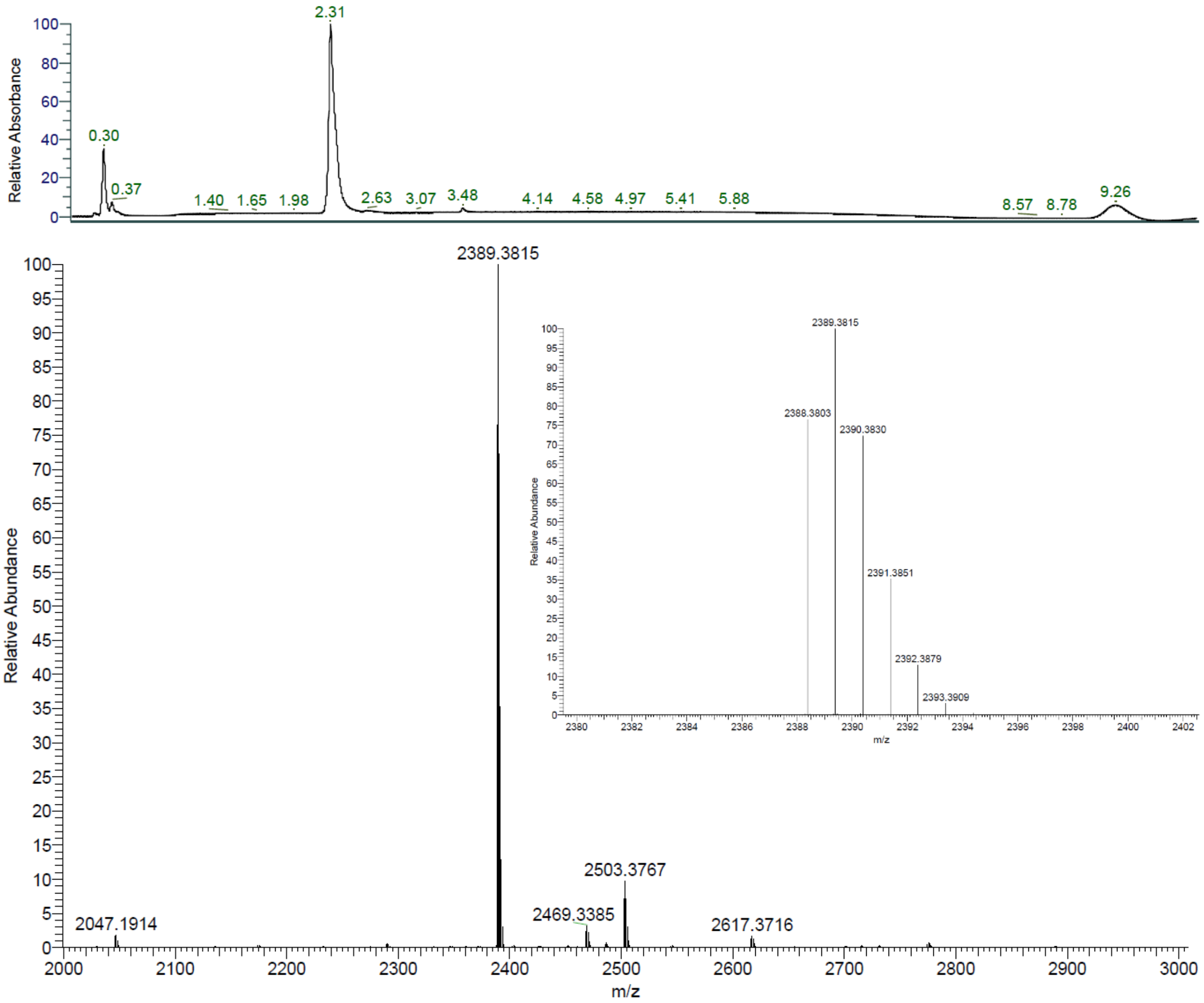

Relative Absorbance
0.30
0.37
1.40
1.65
1.98
2.31
2.63
3.07
3.48
4.14
4.58
4.97
5.41
5.88
8.57
8.78
9.26
Relative Abundance
m/z
2389.3815
2047.1914
2469.3385
2503.3767
2617.3716
2388.3803
2390.3830
2391.3851
2392.3879
2393.3909

**DP12-Onc** VDKPPYLPRPRpPRRIYNR

From Rink Amide AM resin (300 mg, 0.25 mmol/g), the peptide was obtained as a white foamy solid after preparative RP-HPLC purification (13 mg, 6%).

Analytical RP-HPLC: $t_R$ = 2.32 min (A/D = 100/0 to 0/100 in 10.0 min., λ = 214 nm).

HRMS (ESI+): $C_{109}H_{178}N_{37}O_{24}$ calc./found 2389.3840/2389.3820 Da $[M + H]^+$.

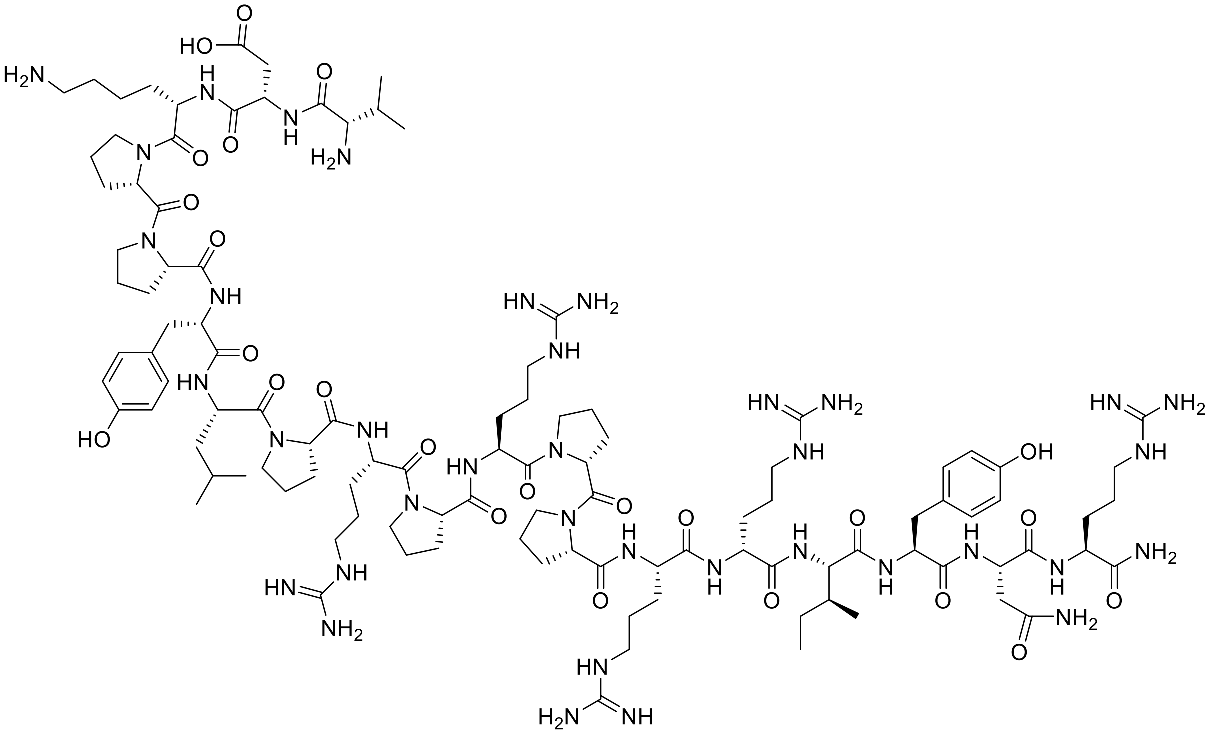

Chemical Formula: $C_{109}H_{177}N_{37}O_{24}$
Exact Mass: 2388.3767
Molecular Weight: 2389.8500

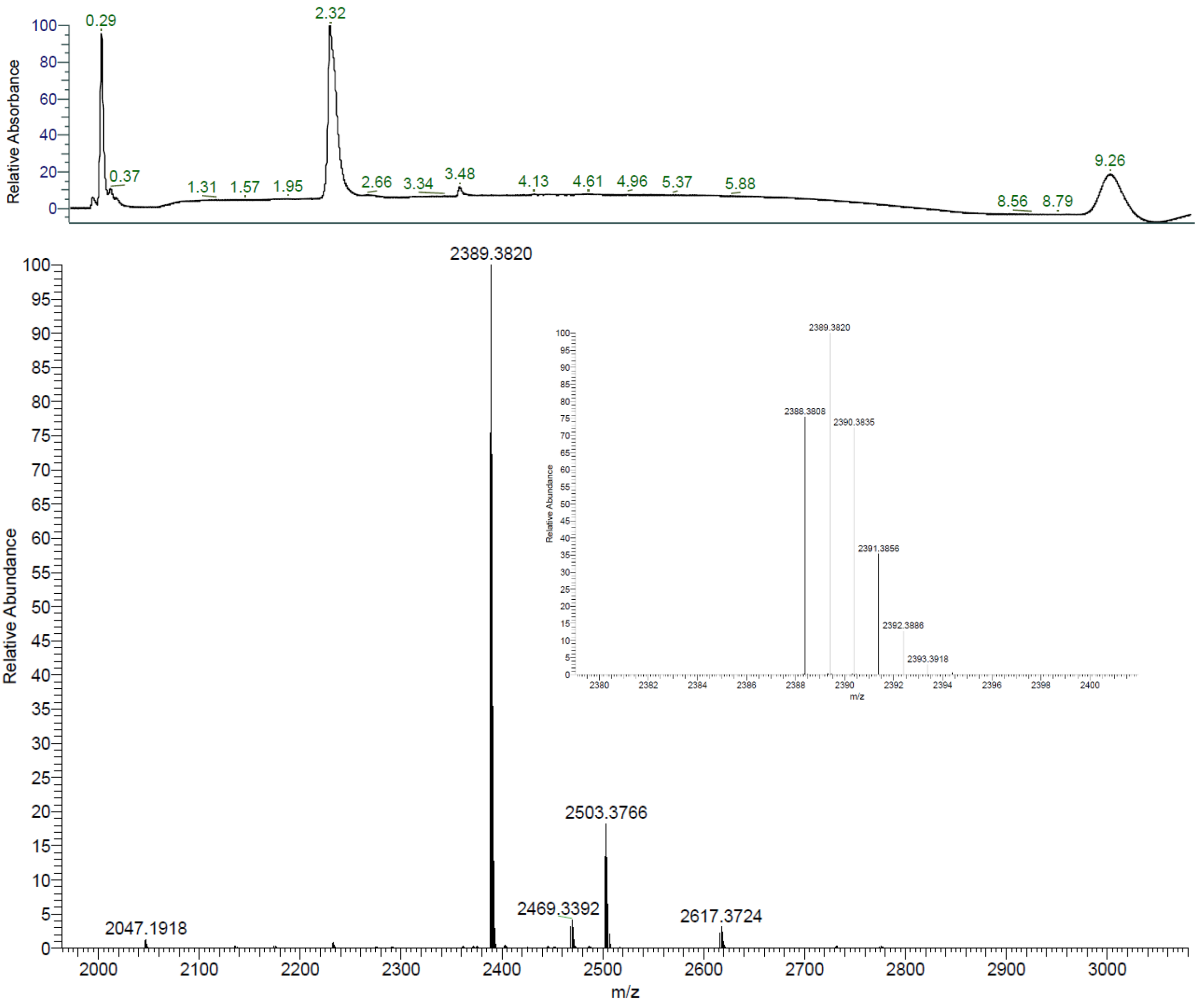
Relative Absorbance
0.29
0.37
1.31
1.57
1.95
2.32
2.66
3.34
3.48
4.13
4.61
4.96
5.37
5.88
8.56
8.79
9.26
Relative Abundance
2389.3820
2503.3766
2469.3392
2617.3724
2047.1918
m/z
2388.3808
2390.3835
2391.3856
2392.3886
2393.3918

**DR11-Onc** VDKPPYLPRPrPPRRIYNR

From Rink Amide AM resin (300 mg, 0.25 mmol/g), the peptide was obtained as a white foamy solid after preparative RP-HPLC purification (50.6 mg, 22%).

Analytical RP-HPLC: $t_R$ = 2.29 min (A/D = 100/0 to 0/100 in 10.0 min., $\lambda$ = 214 nm).

HRMS (ESI+): $C_{109}H_{178}N_{37}O_{24}$ calc./found 2389.3840/2389.3808 Da $[M + H]^+$.

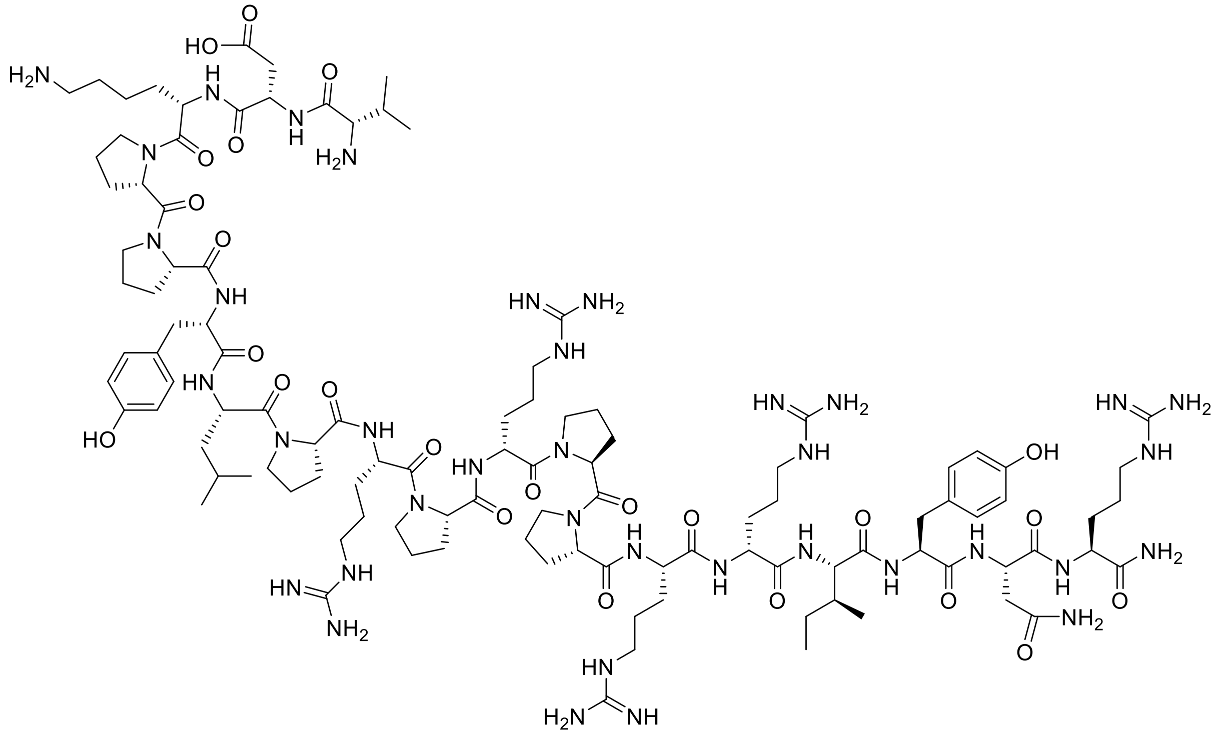

Chemical Formula: $C_{109}H_{177}N_{37}O_{24}$
Exact Mass: 2388.3767
Molecular Weight: 2389.8500

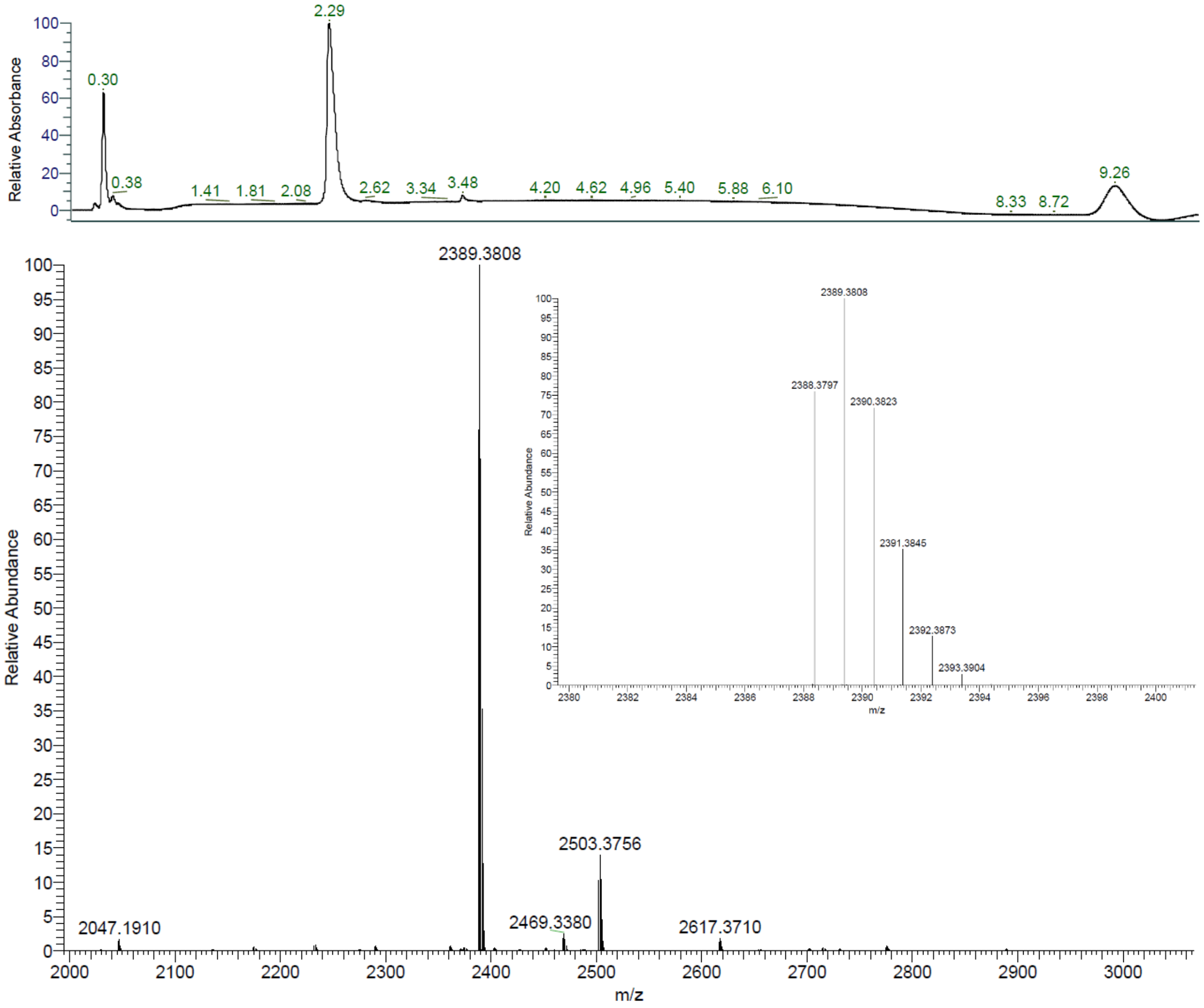

Relative Absorbance
0.30
0.38
1.41
1.81
2.08
2.29
2.62
3.34
3.48
4.20
4.62
4.96
5.40
5.88
6.10
8.33
8.72
9.26
Relative Abundance
2389.3808
2503.3756
2469.3380
2047.1910
2617.3710
2388.3797
2390.3823
2391.3845
2392.3873
2393.3904
m/z

**DP10-Onc** VDKPPYLPRpRPPRRIYNR

From Rink Amide AM resin (300 mg, 0.25 mmol/g), the peptide was obtained as a white foamy solid after preparative RP-HPLC purification (40.7 mg, 18%).

Analytical RP-HPLC: $t_R$ = 2.28 min (A/D = 100/0 to 0/100 in 10.0 min., λ = 214 nm).

HRMS (ESI+): $C_{109}H_{178}N_{37}O_{24}$ calc./found 2389.3840/2389.3810 Da $[M + H]^+$.

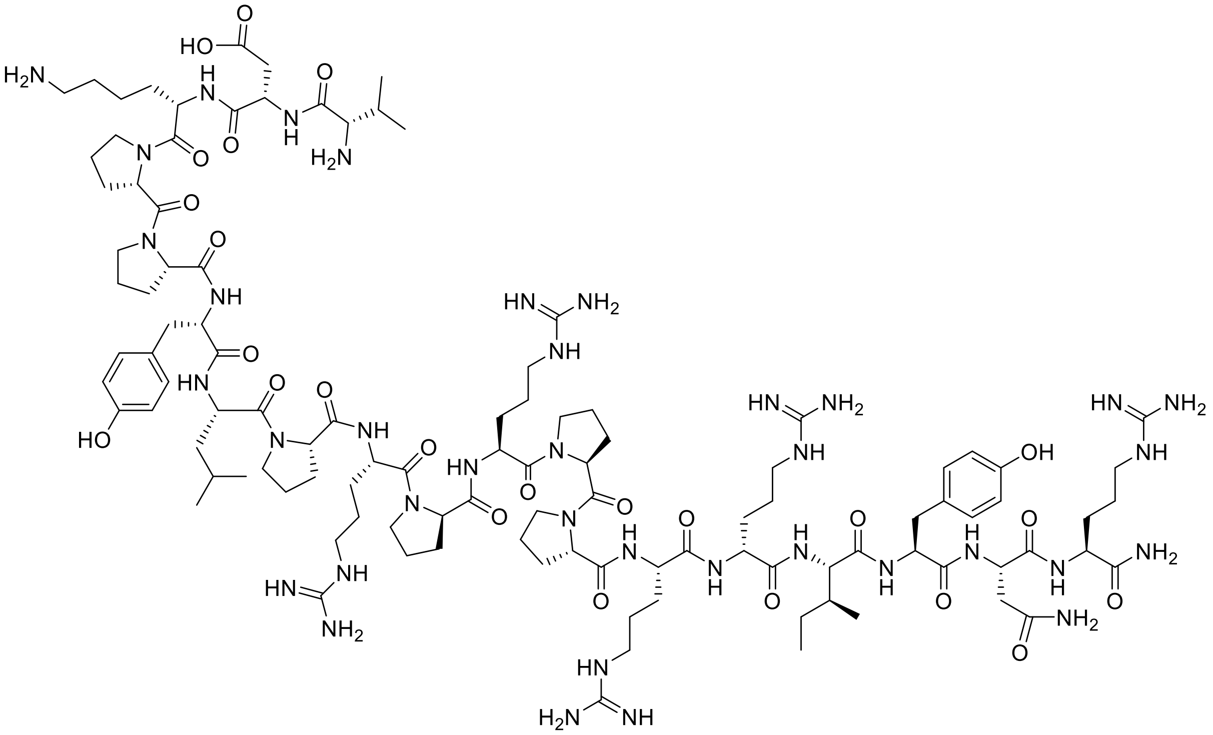

Chemical Formula: $C_{109}H_{177}N_{37}O_{24}$
Exact Mass: 2388.3767
Molecular Weight: 2389.8500

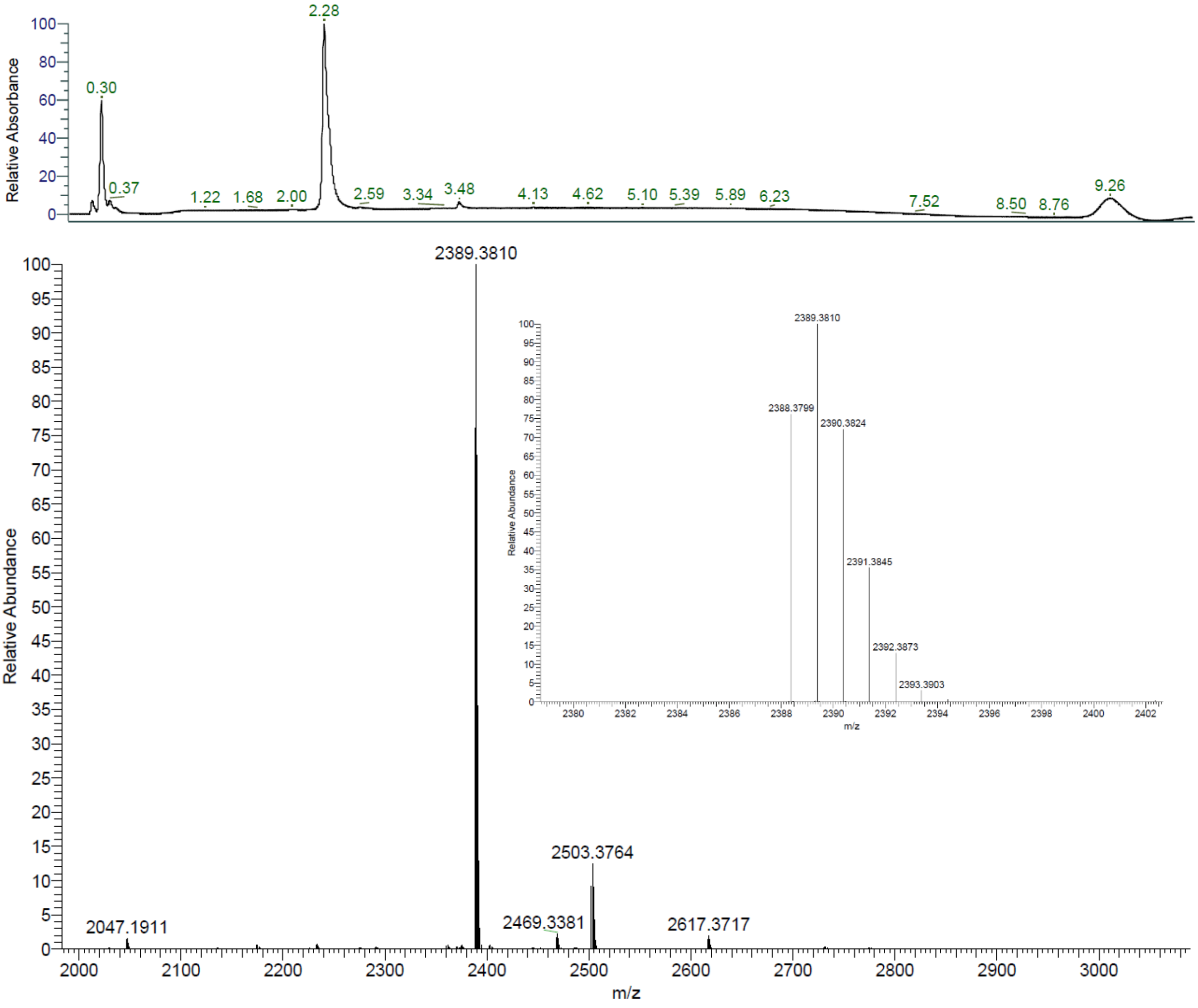
Relative Absorbance
0.30
0.37
1.22
1.68
2.00
2.28
2.59
3.34
3.48
4.13
4.62
5.10
5.39
5.89
6.23
7.52
8.50
8.76
9.26
Relative Abundance
2389.3810
2503.3764
2047.1911
2469.3381
2617.3717
m/z
2388.3799
2390.3824
2391.3845
2392.3873
2393.3903

**DR9-Onc** VDKPPYLPrPRPPRRIYNR

From Rink Amide AM resin (300 mg, 0.29 mmol/g), the peptide was obtained as a white foamy solid after preparative RP-HPLC purification (7.6 mg, 3%).

Analytical RP-HPLC: $t_R$ = 2.29 min (A/D = 100/0 to 0/100 in 10.0 min., λ = 214 nm).

HRMS (ESI+): $C_{109}H_{178}N_{37}O_{24}$ calc./found 2389.3840/2389.3810 Da $[M + H]^+$.

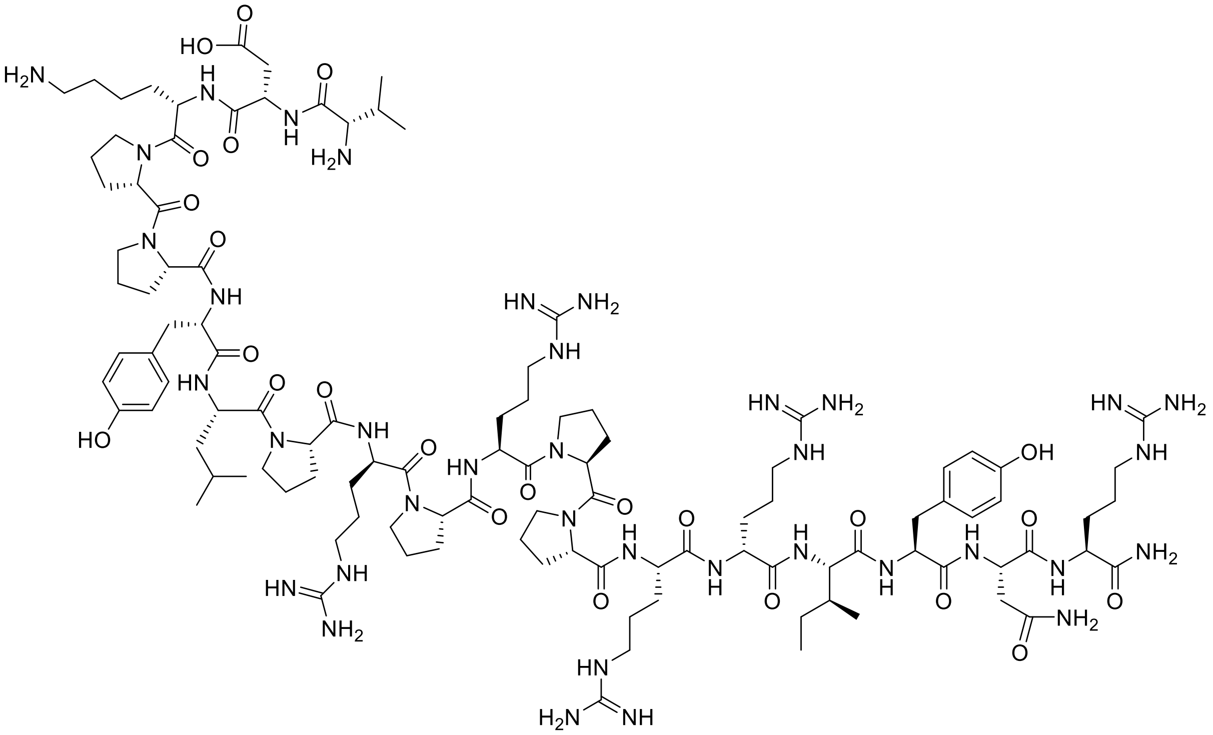

Chemical Formula: $C_{109}H_{177}N_{37}O_{24}$
Exact Mass: 2388.3767
Molecular Weight: 2389.8500

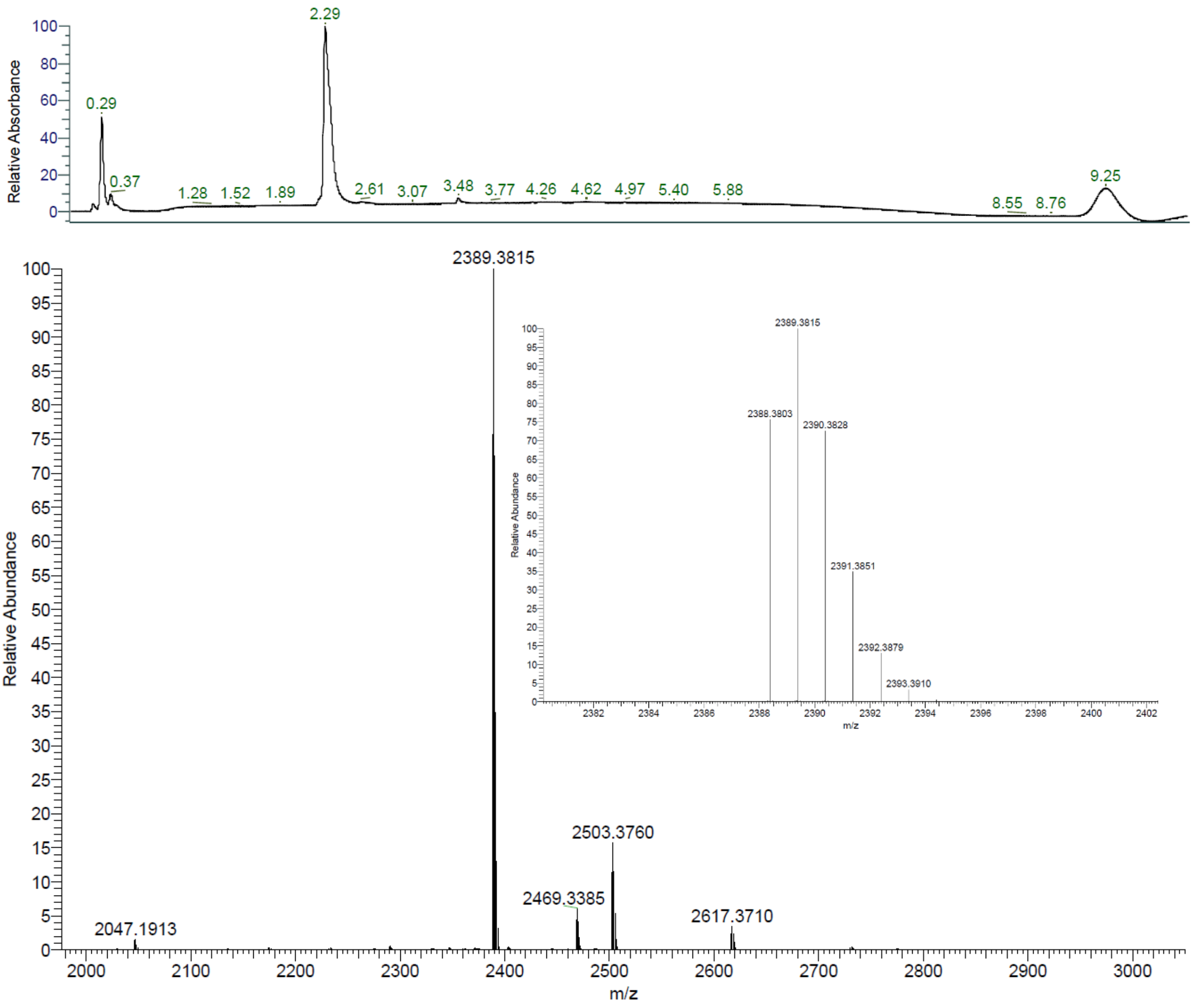

Relative Absorbance
0.29
0.37
1.28
1.52
1.89
2.29
2.61
3.07
3.48
3.77
4.26
4.62
4.97
5.40
5.88
8.55
8.76
9.25
Relative Abundance
2389.3815
2047.1913
2469.3385
2503.3760
2617.3710
m/z
2388.3803
2390.3828
2391.3851
2392.3879
2393.3910

**DP8-Onc** VDKPPYLpRPRPPRRIYNR

From Rink Amide AM resin (305 mg, 0.29 mmol/g), the peptide was obtained as a white foamy solid after preparative RP-HPLC purification (99 mg, 35%).

Analytical RP-HPLC: $t_R$ = 2.33 min (A/D = 100/0 to 0/100 in 10.0 min., λ = 214 nm).

HRMS (ESI+): $C_{109}H_{178}N_{37}O_{24}$ calc./found 2389.3840/2389.3758 Da $[M + H]^+$.

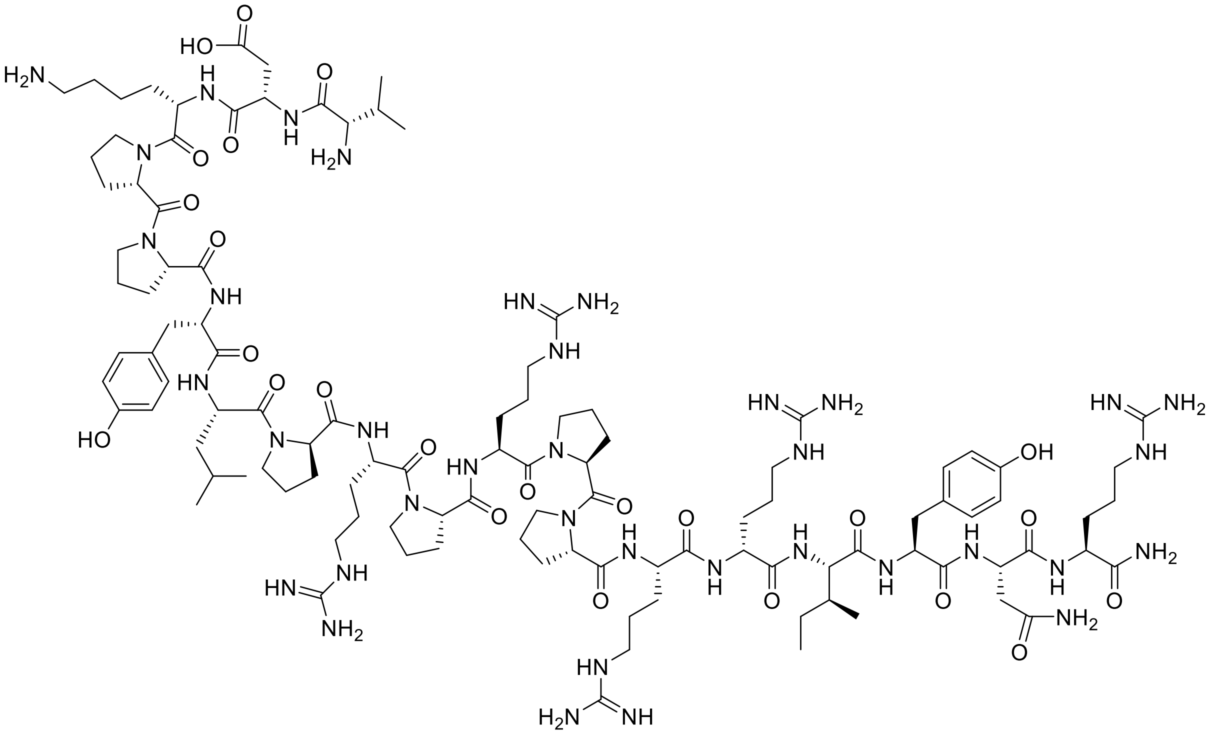

Chemical Formula: $C_{109}H_{177}N_{37}O_{24}$
Exact Mass: 2388.3767
Molecular Weight: 2389.8500

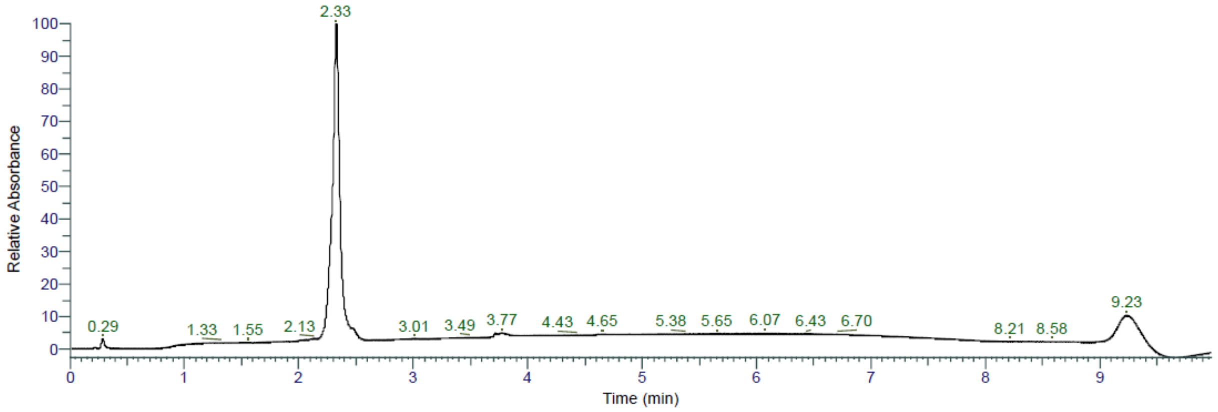
2.33
Relative Absorbance
0.29
1.33
1.55
2.13
3.01
3.49
3.77
4.43
4.65
5.38
5.65
6.07
6.43
6.70
8.21
8.58
9.23
Time (min)

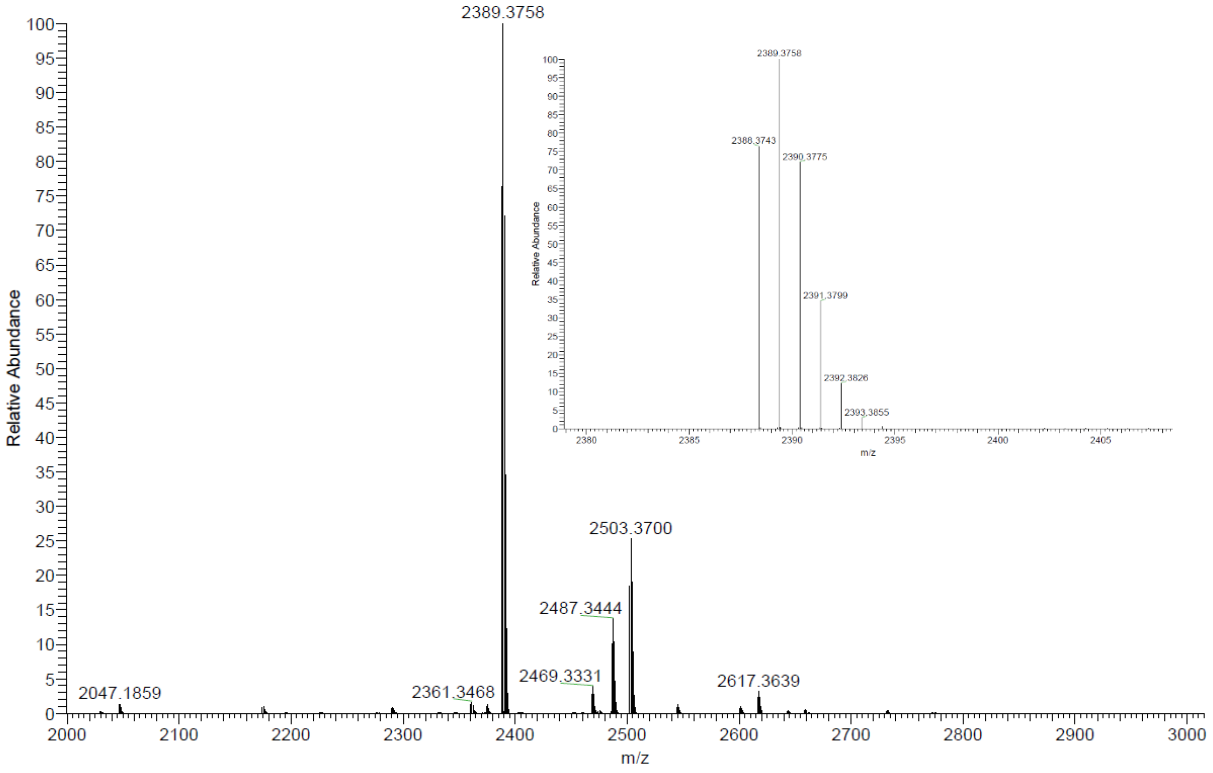
2389.3758
Relative Abundance
2503.3700
2487.3444
2469.3331
2617.3639
2361.3468
2047.1859
m/z
2388.3743
2390.3775
2391.3799
2392.3826
2393.3855

**DL7-Onc** VDKPPYlPRPRPPRRIYNR

From Rink Amide AM resin (326 mg, 0.29 mmol/g), the peptide was obtained as a white foamy solid after preparative RP-HPLC purification (110 mg, 37%).

Analytical RP-HPLC: $t_R$ = 2.38 min (A/D = 100/0 to 0/100 in 10.0 min., $\lambda$ = 214 nm).

HRMS (ESI+): $C_{109}H_{178}N_{37}O_{24}$ calc./found 2389.3840/2389.3763 Da $[M + H]^+$.

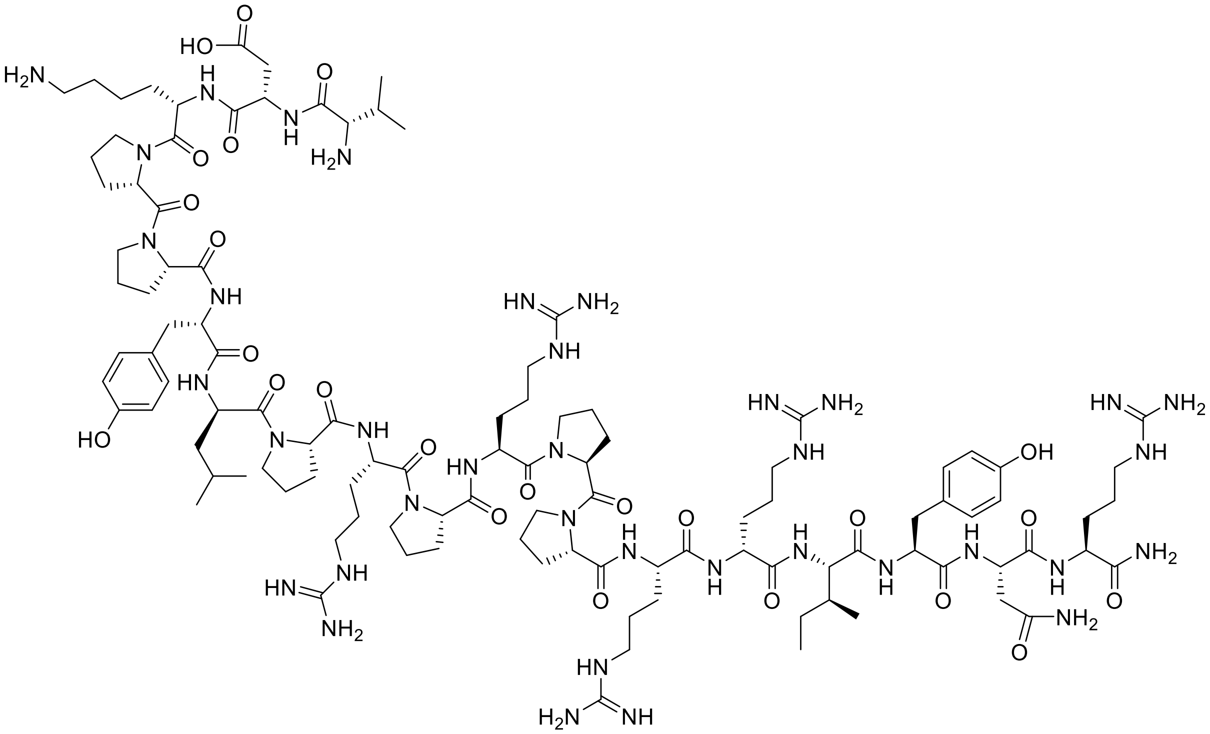

Chemical Formula: $C_{109}H_{177}N_{37}O_{24}$
Exact Mass: 2388.3767
Molecular Weight: 2389.8500

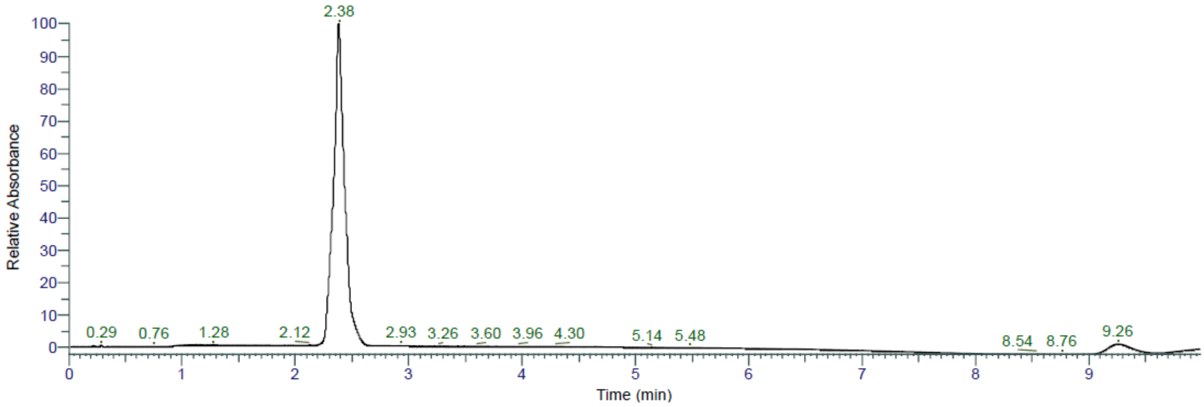
2.38
0.29
0.76
1.28
2.12
2.93
3.26
3.60
3.96
4.30
5.14
5.48
8.54
8.76
9.26
Relative Absorbance
Time (min)

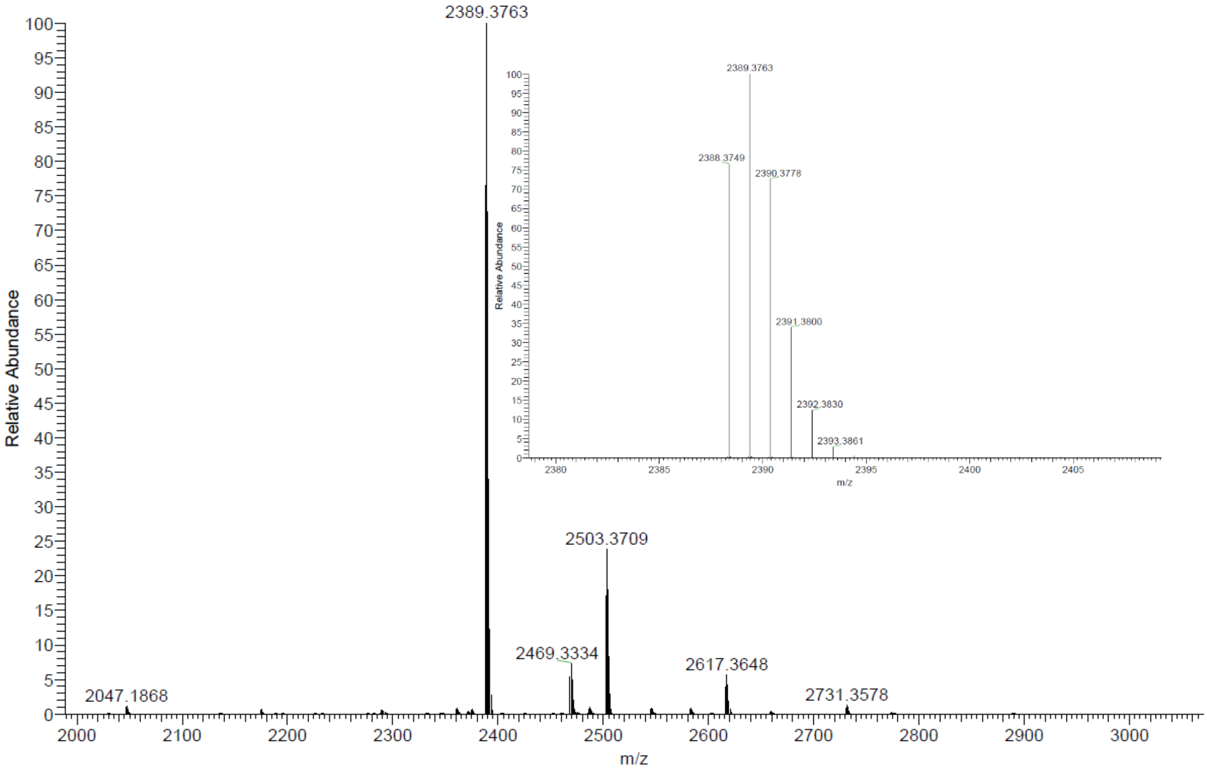
2389.3763
2503.3709
2469.3334
2617.3648
2731.3578
2047.1868
Relative Abundance
m/z
2388.3749
2390.3778
2391.3800
2392.3830
2393.3861

**DY6-Onc** VDKPPyLPRPRPPRRIYNR

From Rink Amide AM resin (329 mg, 0.29 mmol/g), the peptide was obtained as a white foamy solid after preparative RP-HPLC purification (130 mg, 43%).

Analytical RP-HPLC: $t_R$ = 2.34 min (A/D = 100/0 to 0/100 in 10.0 min., $\lambda$ = 214 nm).

HRMS (ESI+): $C_{109}H_{178}N_{37}O_{24}$ calc./found 2389.3840/2389.3782 Da $[M + H]^+$.

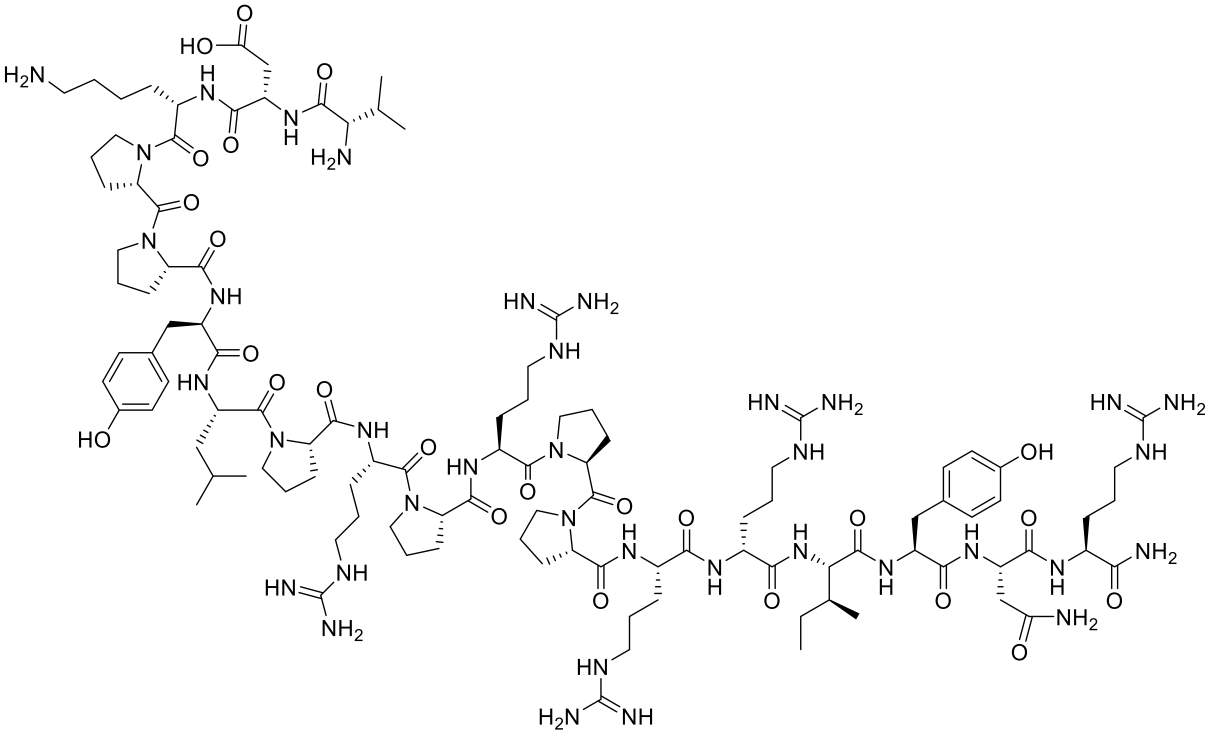

Chemical Formula: $C_{109}H_{177}N_{37}O_{24}$
Exact Mass: 2388.3767
Molecular Weight: 2389.8500

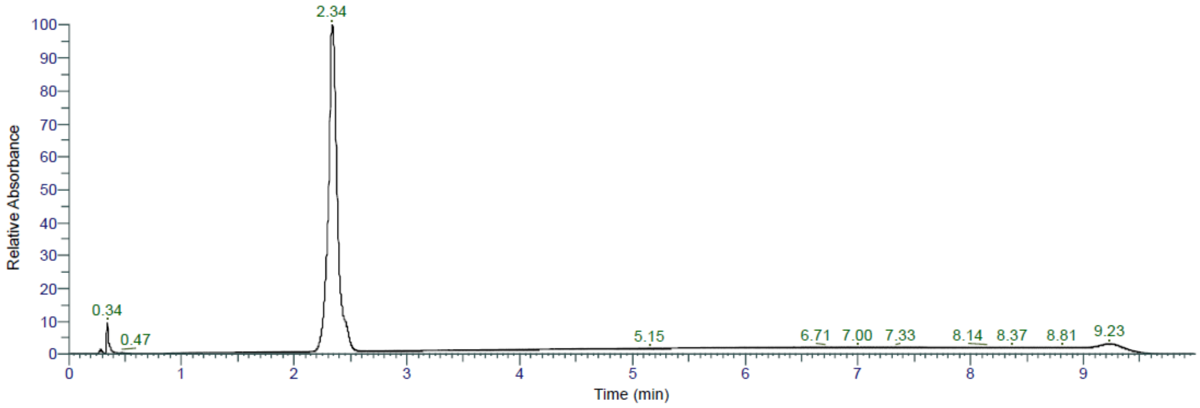
2.34
0.34
0.47
5.15
6.71
7.00
7.33
8.14
8.37
8.81
9.23
Relative Absorbance
Time (min)

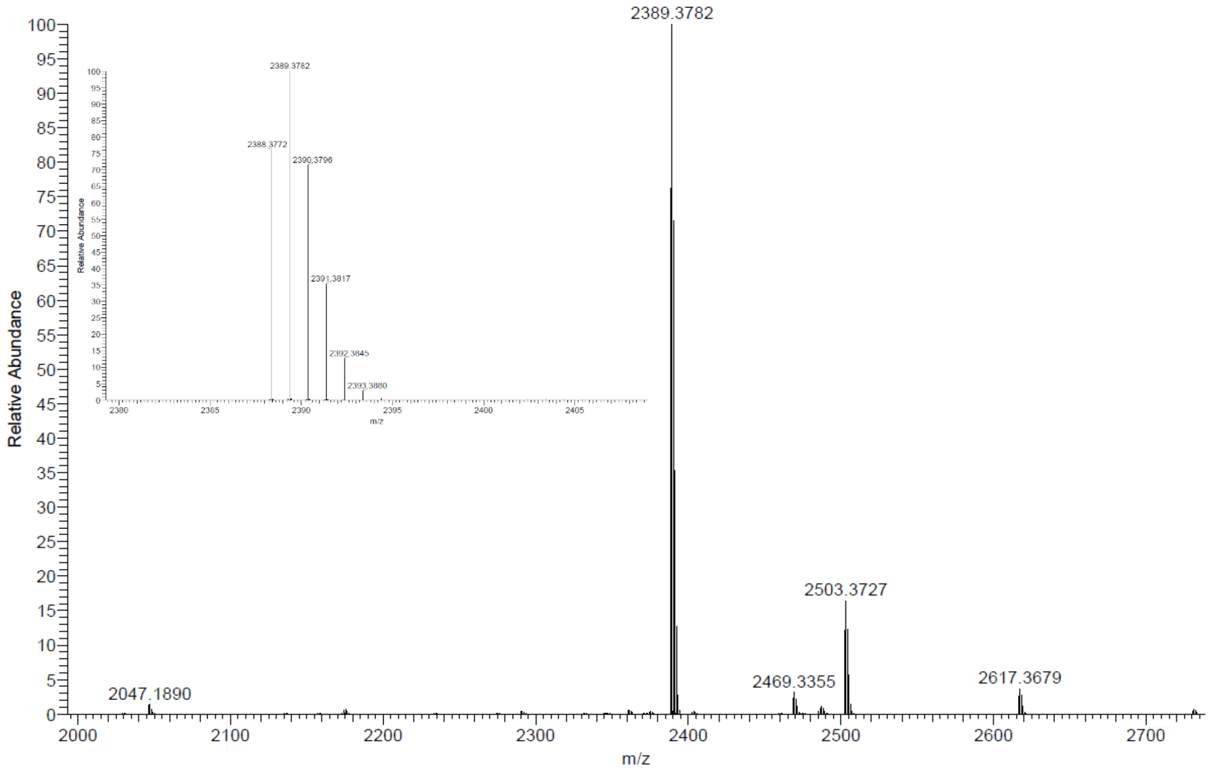
2389.3782
2503.3727
2469.3355
2617.3679
2047.1890
Relative Abundance
m/z
2388.3772
2390.3796
2391.3817
2392.3845
2393.3880

**DP5-Onc** VDKPpYLPRPRPPRRIYNR

From Rink Amide AM resin (315 mg, 0.29 mmol/g), the peptide was obtained as a white foamy solid after preparative RP-HPLC purification (98 mg, 34%).

Analytical RP-HPLC: $t_R$ = 2.23 min (A/D = 100/0 to 0/100 in 10.0 min., λ = 214 nm).

HRMS (ESI+): $C_{109}H_{178}N_{37}O_{24}$ calc./found 2389.3840/2389.3773 Da $[M + H]^+$.

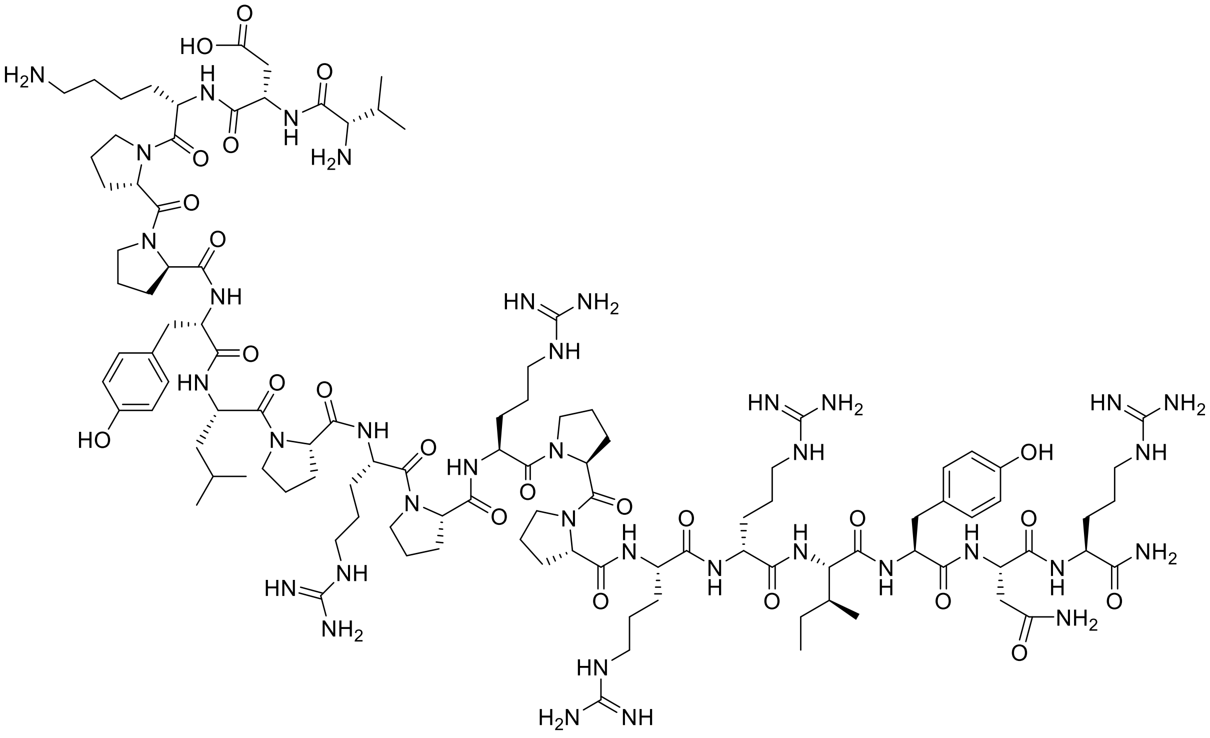

Chemical Formula: $C_{109}H_{177}N_{37}O_{24}$
Exact Mass: 2388.3767
Molecular Weight: 2389.8500

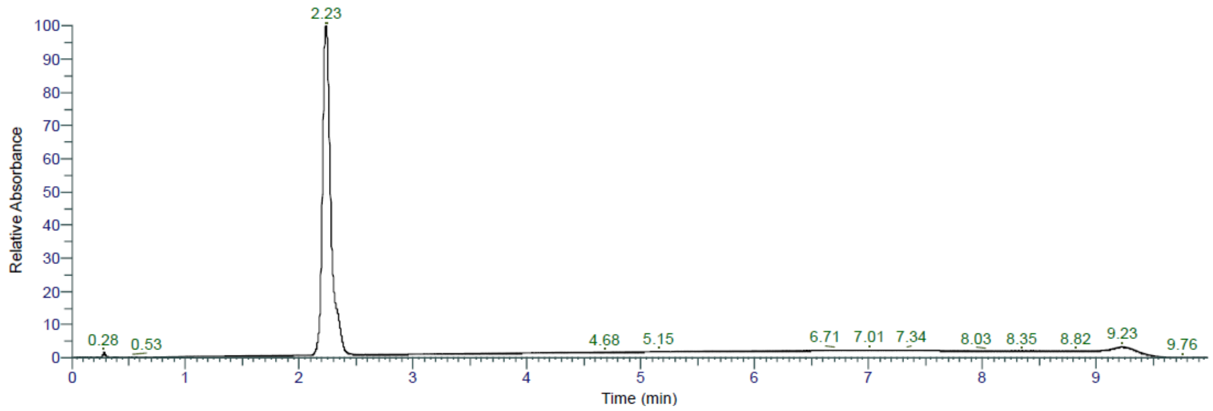
2.23
0.28
0.53
4.68
5.15
6.71
7.01
7.34
8.03
8.35
8.82
9.23
9.76
Relative Absorbance
Time (min)

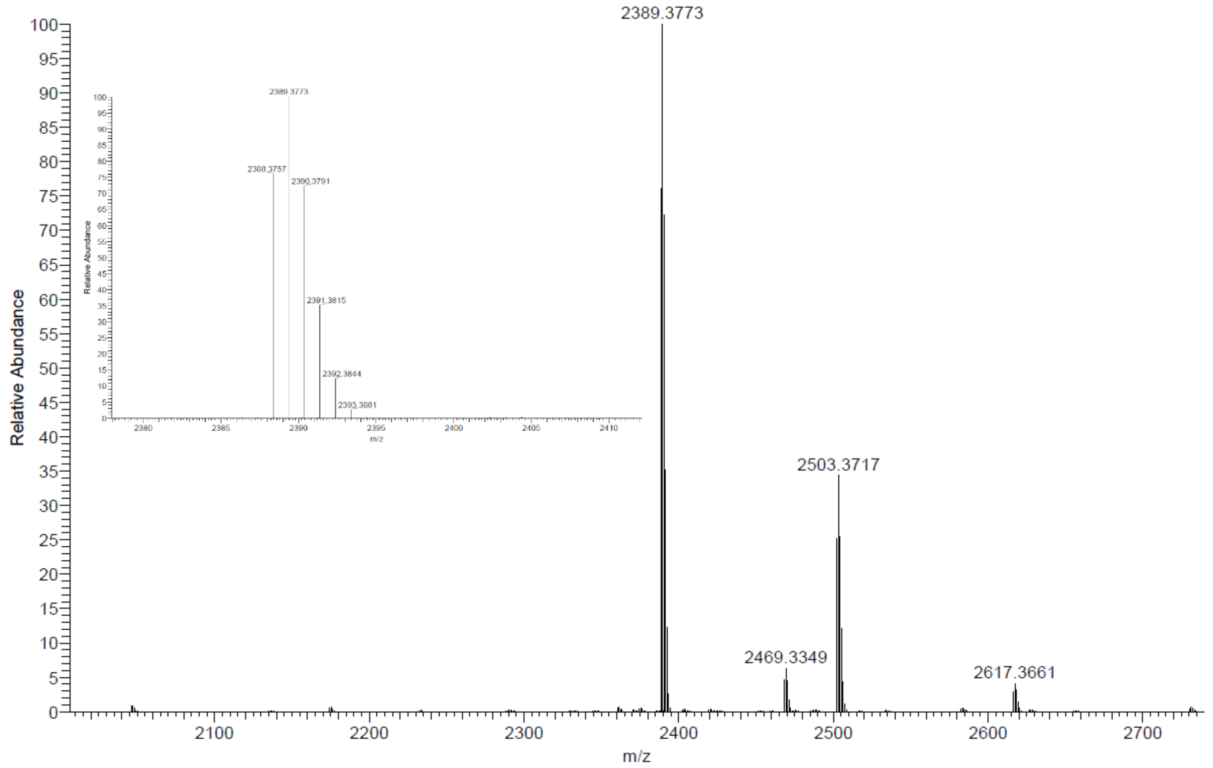
2389.3773
2503.3717
2469.3349
2617.3661
Relative Abundance
m/z

**DP45-Onc** VDKppYLPRPRPPRRIYNR

From Rink Amide AM resin (335 mg, 0.29 mmol/g), the peptide was obtained as a white foamy solid after preparative RP-HPLC purification (104 mg, 34%).

Analytical RP-HPLC: $t_R$ = 2.31 min (A/D = 100/0 to 0/100 in 10.0 min., λ = 214 nm).

HRMS (ESI+): $C_{109}H_{178}N_{37}O_{24}$ calc./found 2389.3840/2389.3746 Da $[M + H]^+$.

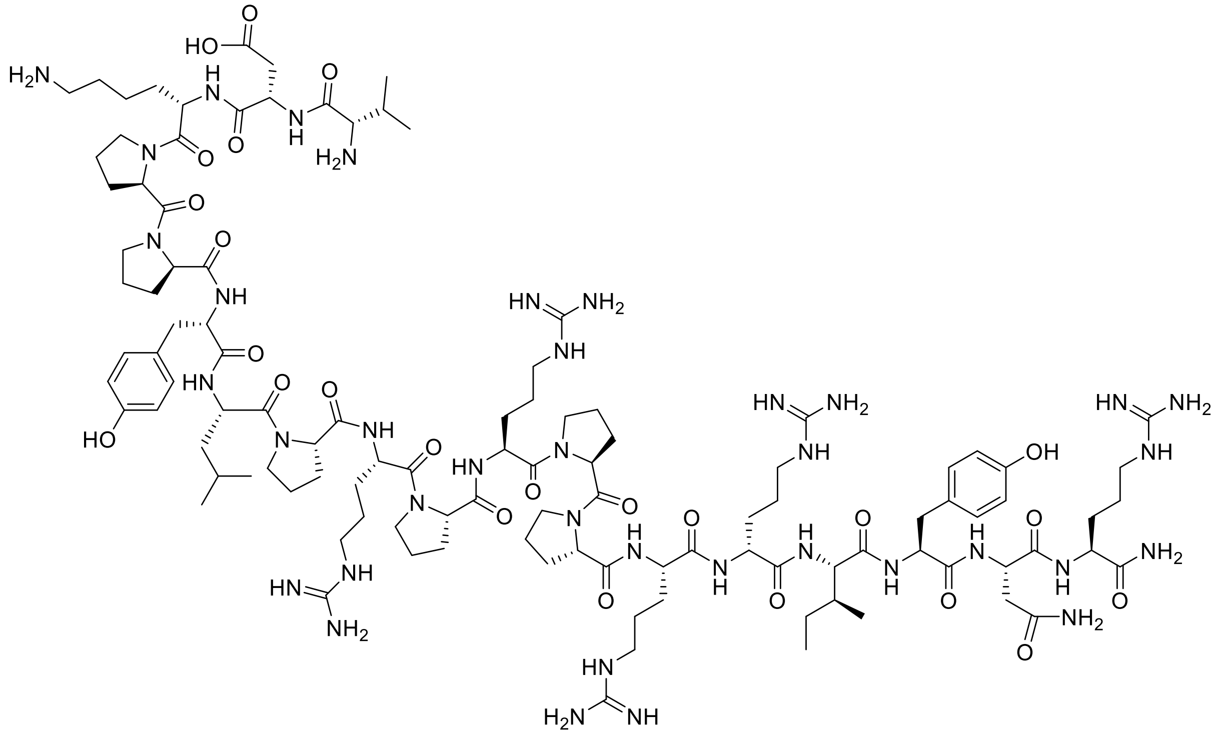

Chemical Formula: $C_{109}H_{177}N_{37}O_{24}$
Exact Mass: 2388.3767
Molecular Weight: 2389.8500

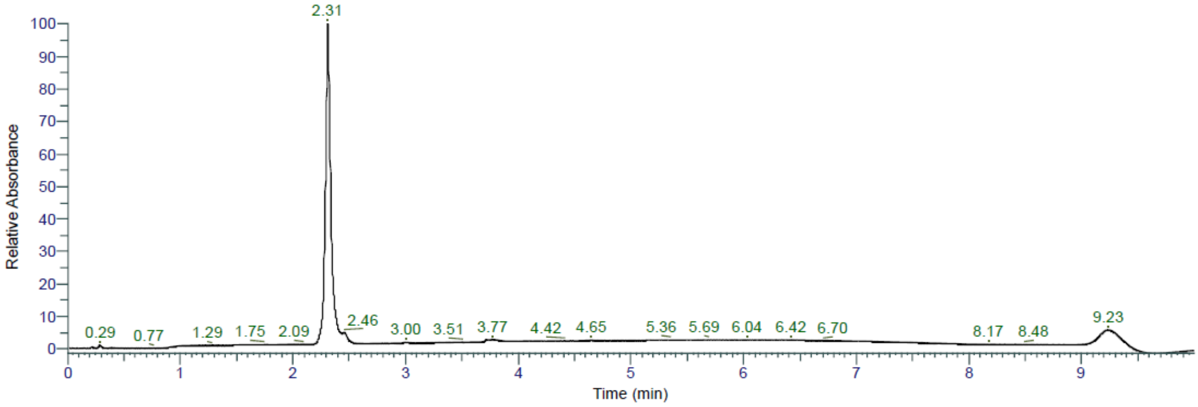
Relative Absorbance
Time (min)
2.31
9.23

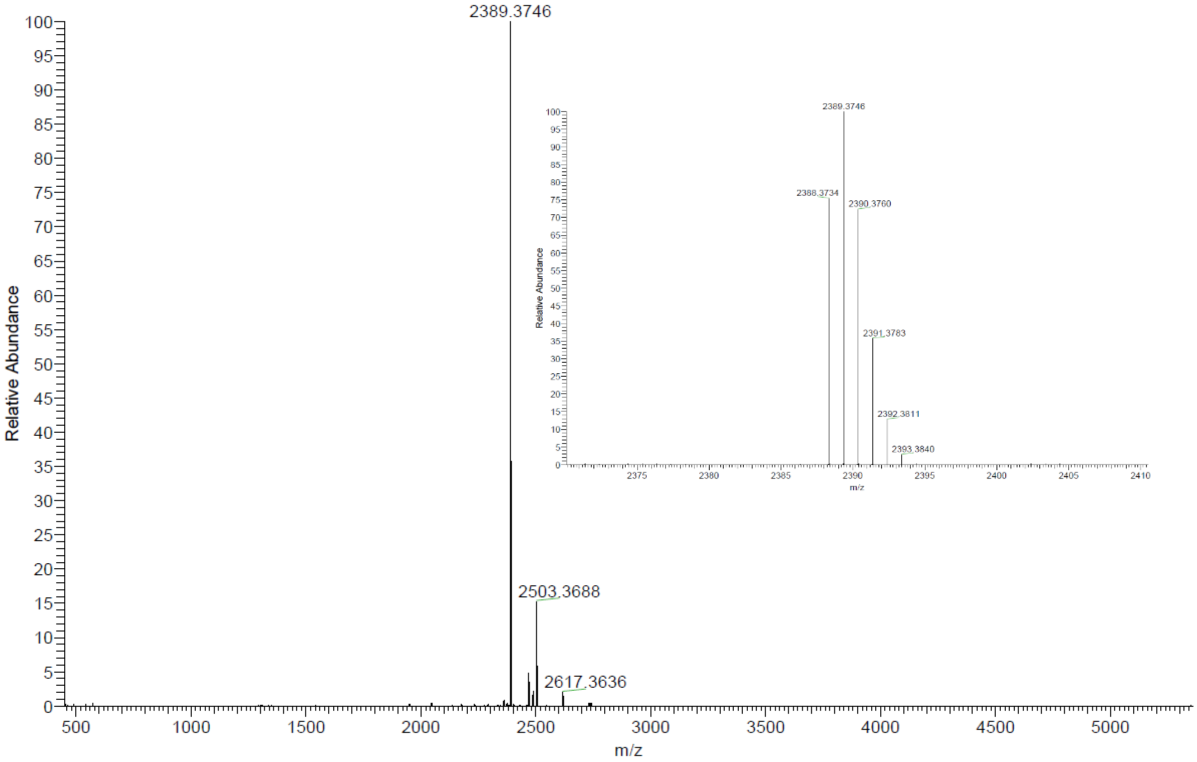
Relative Abundance
m/z
2389.3746
2503.3688
2617.3636
2388.3734
2390.3760
2391.3783
2392.3811
2393.3840

**DP4-Onc** VDKpPYLPRPRPPRRIYNR

From Rink Amide AM resin (310 mg, 0.29 mmol/g), the peptide was obtained as a white foamy solid after preparative RP-HPLC purification (120 mg, 42%).

Analytical RP-HPLC: $t_R$ = 2.31 min (A/D = 100/0 to 0/100 in 10.0 min., λ = 214 nm).

HRMS (ESI+): $C_{109}H_{178}N_{37}O_{24}$ calc./found 2389.3840/2389.3770 Da $[M + H]^+$.

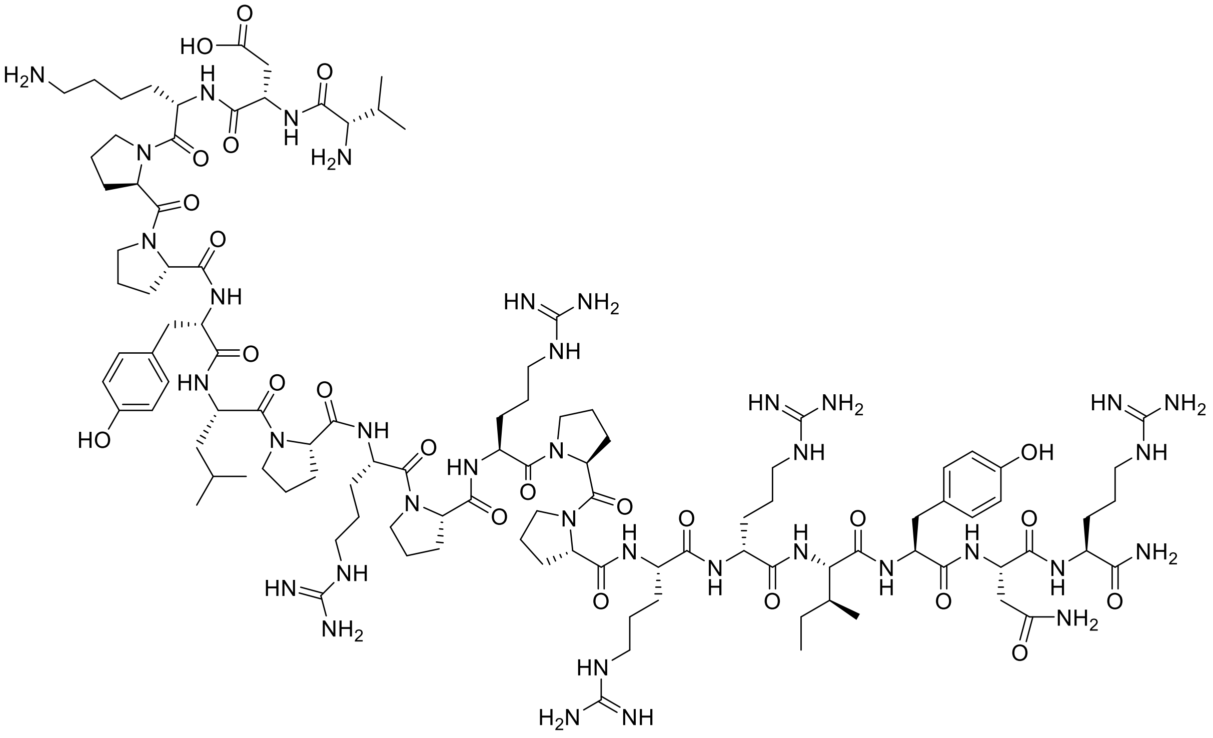

Chemical Formula: $C_{109}H_{177}N_{37}O_{24}$
Exact Mass: 2388.3767
Molecular Weight: 2389.8500

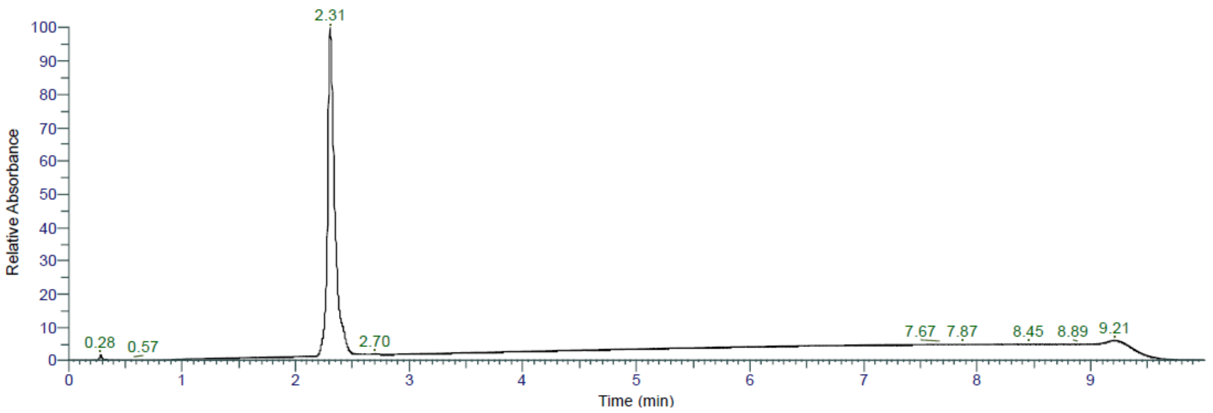
2.31
0.28
0.57
2.70
7.67
7.87
8.45
8.89
9.21
Relative Absorbance
Time (min)

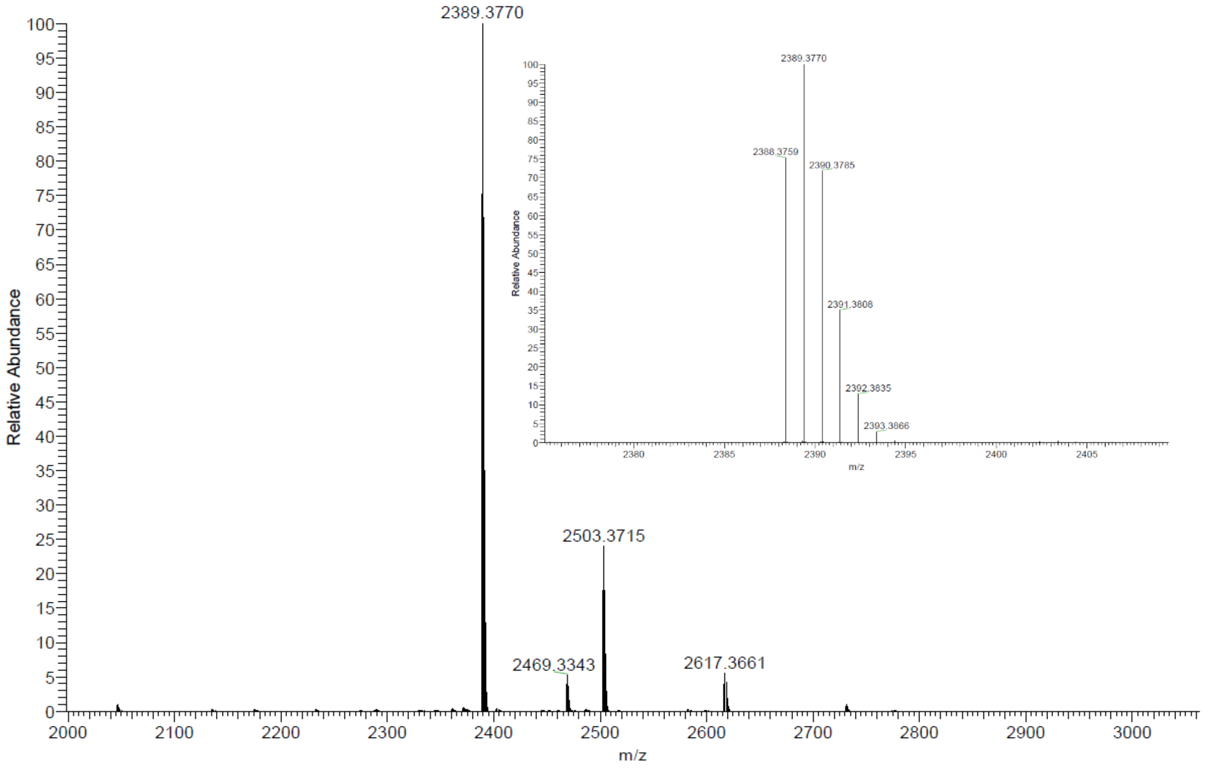
2389.3770
2503.3715
2469.3343
2617.3661
Relative Abundance
m/z
2388.3759
2390.3785
2391.3808
2392.3835
2393.3866

**D-Onc** vdkppylprprpprriynr

From Rink Amide AM resin (348 mg, 0.29 mmol/g), the peptide was obtained as a white foamy solid after preparative RP-HPLC purification (96 mg, 30%).

Analytical RP-HPLC: $t_R$ = 2.36 min (A/D = 100/0 to 0/100 in 10.0 min., λ = 214 nm).

HRMS (ESI+): $C_{109}H_{178}N_{37}O_{24}$ calc./found 2389.3840/2389.3860 Da $[M + H]^+$.

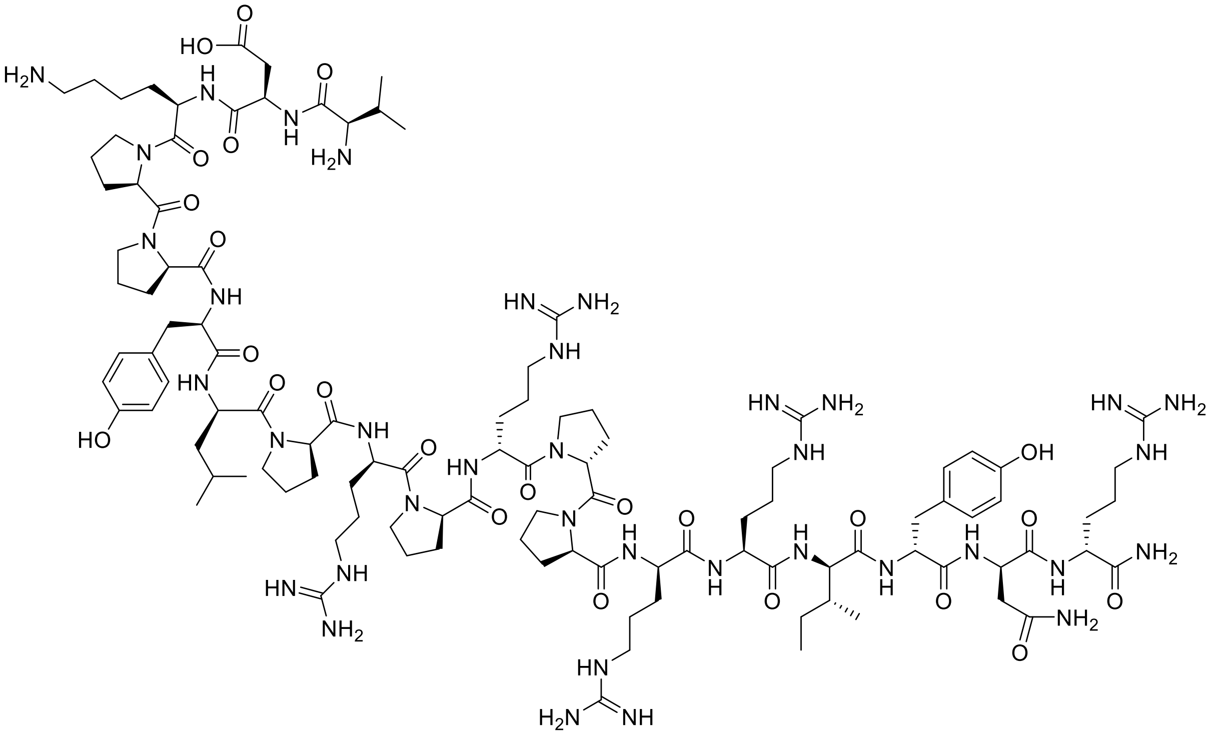

Chemical Formula: $C_{109}H_{177}N_{37}O_{24}$
Exact Mass: 2388.3767
Molecular Weight: 2389.8500

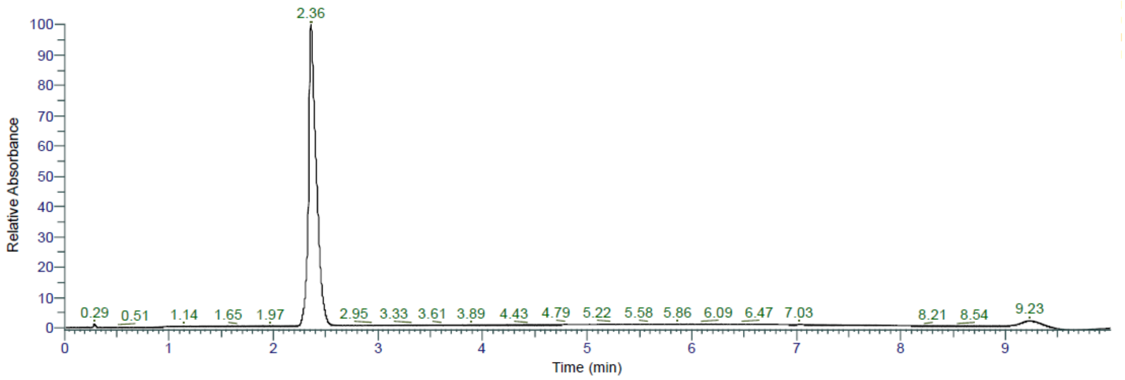
2.36
Relative Absorbance
0.29
0.51
1.14
1.65
1.97
2.95
3.33
3.61
3.89
4.43
4.79
5.22
5.58
5.86
6.09
6.47
7.03
8.21
8.54
9.23
Time (min)

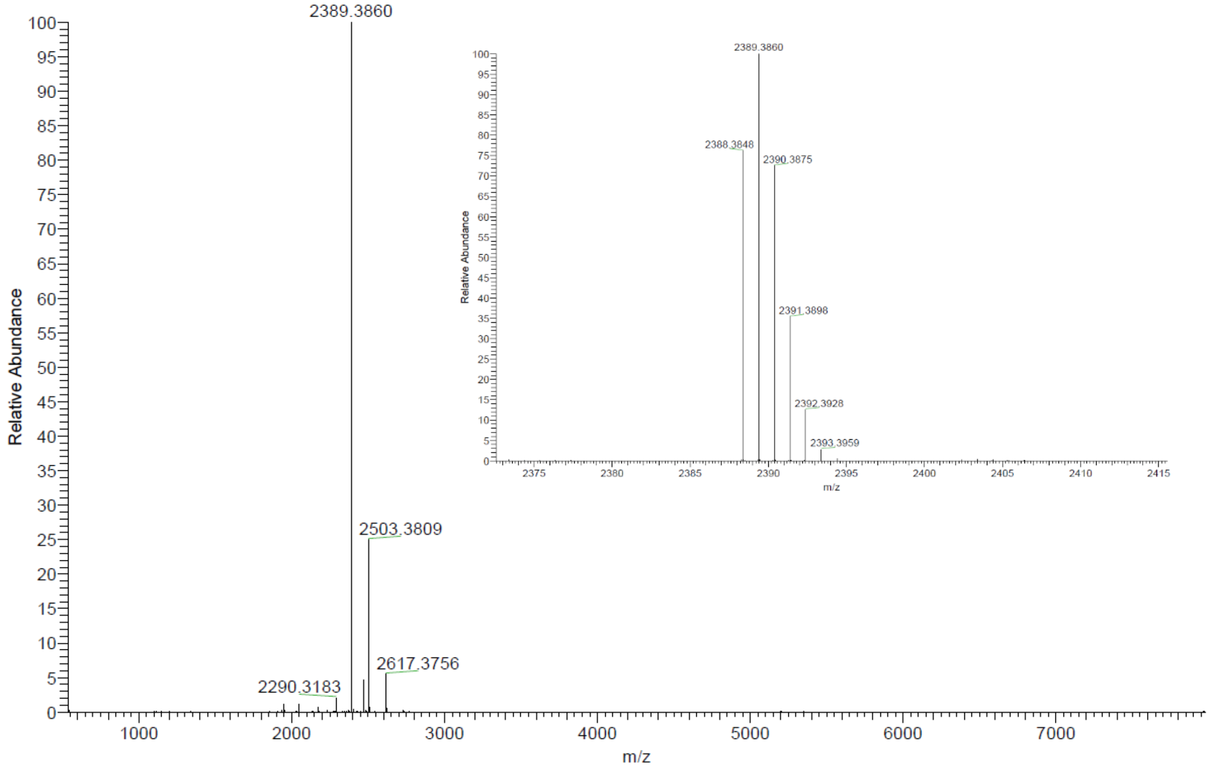
2389.3860
Relative Abundance
2503.3809
2617.3756
2290.3183
m/z
2388.3848
2390.3875
2391.3898
2392.3928
2393.3959

## Microbiology

### Minimum inhibitory concentration assay

Antimicrobial activity was assayed against *P. aeruginosa* PAO1, *K. pneumoniae* NCTC418, *E. coli* W3110, *A. baumannii* ATCC 19606, methicillin-resistant *S. aureus* COL (MRSA), and for selected peptides on *P. aeruginosa* PA14 and the polymyxin-B-resistant derivative PA14 2P4, as well as the clinical isolates ZEM-1A and ZEM9A, *K. pneumoniae* OXA-48, *S. aureus* Newman, *Enterobacter cloacae* and *Burkholderia cenocepacia*. The minimal inhibitory concentration (MIC) was determined by using broth microdilution method. A colony of bacteria was picked and grown in Luria-Bertani (LB) medium overnight at 37°C. For *E. coli* BW25113 *sbmA::kan* the medium was supplemented with 25 mg/L of kanamycin. The next day, the culture was then regrown in LB medium to log phase ($OD_{600}$ = 0.6 to 0.8), which lasted approximately 4 h, and diluted to an $OD_{600}$ of 0.022 in the desired medium. Stock solutions of 1 mg/mL of the samples were prepared in sterilized milliQ water and diluted to the beginning concentration of 64 μg/mL in 300 μL Mueller Hinton (MH)-medium. The diluted samples were added to first well of 96-well microtiter plate (TPP, untreated, Faust Laborbedarf, AG, Schaffhausen) and diluted serially by ½. Finally, 4 μL of the bacterial suspension was inoculated into the sample solutions (150 μL/well) with a final inoculation of about $5 \cdot 10^5$ CFU/mL. The plates were then incubated at 37°C for 15-18h. For each assay, sterility (broth only) and growth control (broth with bacterial inoculum, without antibiotics) were checked with two columns in the plate. The next day, 15 μL of 3-(4,5-dimethylthiazol-2-yl)-2,5-diphenyltetrazolium bromide (MTT) was added to each well of the plate, such a way that MIC was defined as the lowest concentration of the peptide that inhibited visible growth of the tested bacteria.

**Table S1.** Activity of stereorandomized and diastereomeric oncocins against *E. coli* BW25113 WT and BW25113 *sbmA::kan* mutant in full and 12.5% MH, at pH 7.4.

| | Full MH[a)] | | 12.5% MH[a)] | |
|---|---|---|---|---|
| | WT | SbmA- | WT | SbmA- |
| L-**Onc** | 16-32 | 64 - >64 | 4 | 8 |
| **Onc112** | 16 | > 64 | 4 | 4 |
| *sr9Cterm*-**Onc** | 32 | > 64 | 4 | 8 |
| D-**Onc** | > 64 | > 64 | 16 | 16 |
| *sr*-**Onc** | > 64 | > 64 | 8 | 8-16 |
| *sr14Nterm*-**Onc** | > 64 | > 64 | 16 | 16 |
| DL7-**Onc** | > 64 | > 64 | 32 | 32 |
| **PMB** | 2 | 1 | 1-2 | 1 |

a) Minimum inhibitory concentration (MIC, µg/mL) was determined on *E. coli* BW25113 WT and *sbmA::kan* mutant in full and 12.5% Müller-Hinton medium, after incubation for 16-20 h at 37 °C. In full MH, L-**Onc**, **Onc112** and *sr9Cterm*-**Onc** lost their activities against *E. coli* BW25113 SbmA deletion mutant. There was no change in activity for inactive compounds. In 12.5 % MH, all the compounds exhibited activities that were conserved on the SbmA deletion mutant.

## Bacteria growth

A single colony of *P. aeruginosa* PAO1, *K. pneumoniae* NCTC418, *E. coli* W3110, *A. baumannii* ATCC 19606, and *S. aureus* COL (MRSA strain) was picked and grown overnight with shaking (180 rpm) in 5 mL of LB (Sigma Aldrich, Buchs, Switzerland) medium overnight at 37 °C. The overnight bacterial culture was diluted to $OD_{600}$ of 0.002 ($2 \times 10^6$ CFU/mL) in fresh, diluted, or full MH (Sigma Aldrich, Buchs, Switzerland, full media at pH 7.4 and 12.5% at pH 8.5) medium. 100 µL of the prepared bacteria solution in MH and 100 µL of the corresponding MH (full or diluted) were mixed in 96-well microtiter plates (TPP, untreated, Corning Incorporated, Kennebunk, USA). The 96-well microtiter plates were incubated at 37 °C with shaking (180 rpm). Bacteria were quantified at 0, 1, 2, 3, 4, 5, and 7 h by plating 10-fold dilutions of the sample in sterilized normal saline (NaCl 0.9%) on LB agar plates. The plates were then incubated at 37 °C for 14–16 h, and the number of individual colonies was counted for each time-point. The assay was performed twice in triplicate.

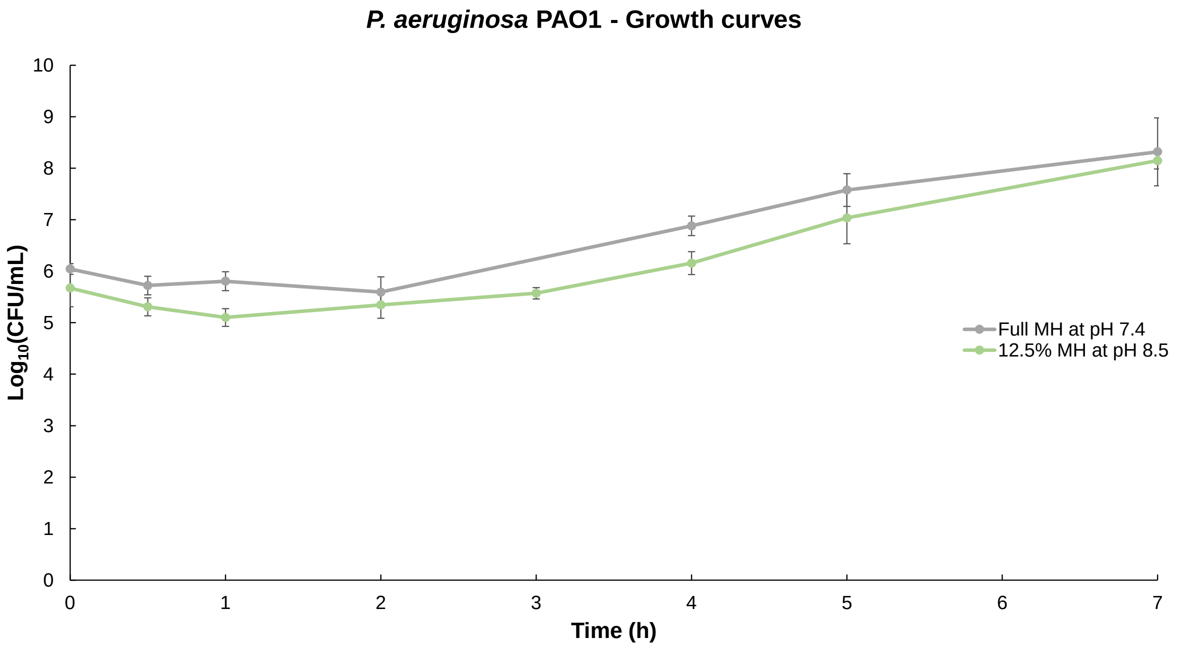
P. aeruginosa PAO1 - Growth curves
Log10(CFU/mL)
10
9
8
7
6
5
4
3
2
1
0
0
1
2
3
4
5
6
7
Time (h)
Full MH at pH 7.4
12.5% MH at pH 8.5

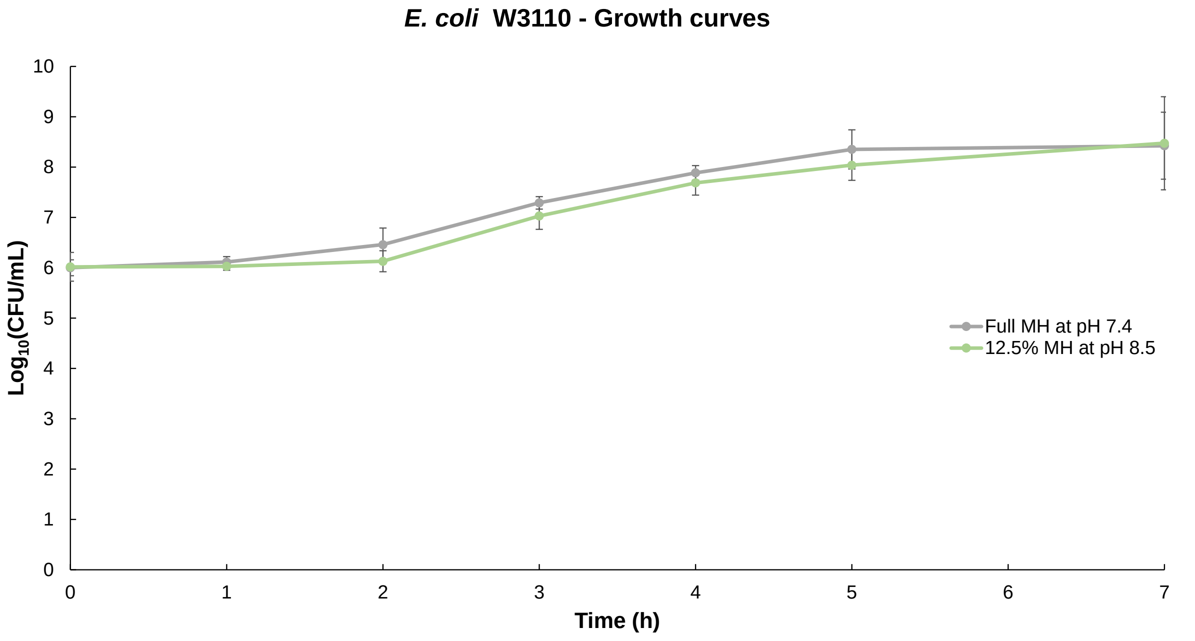
E. coli W3110 - Growth curves
Log10(CFU/mL)
10
9
8
7
6
5
4
3
2
1
0
0
1
2
3
4
5
6
7
Time (h)
Full MH at pH 7.4
12.5% MH at pH 8.5

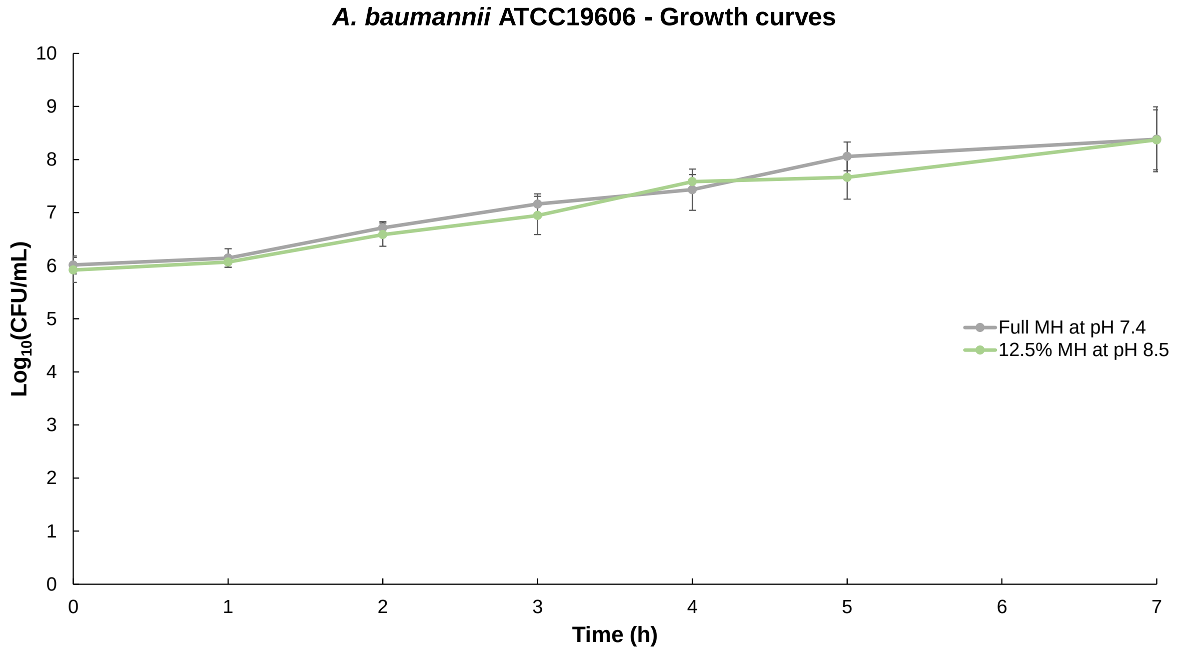
A. baumannii ATCC19606 - Growth curves
Log10(CFU/mL)
0
1
2
3
4
5
6
7
8
9
10
Time (h)
0
1
2
3
4
5
6
7
Full MH at pH 7.4
12.5% MH at pH 8.5

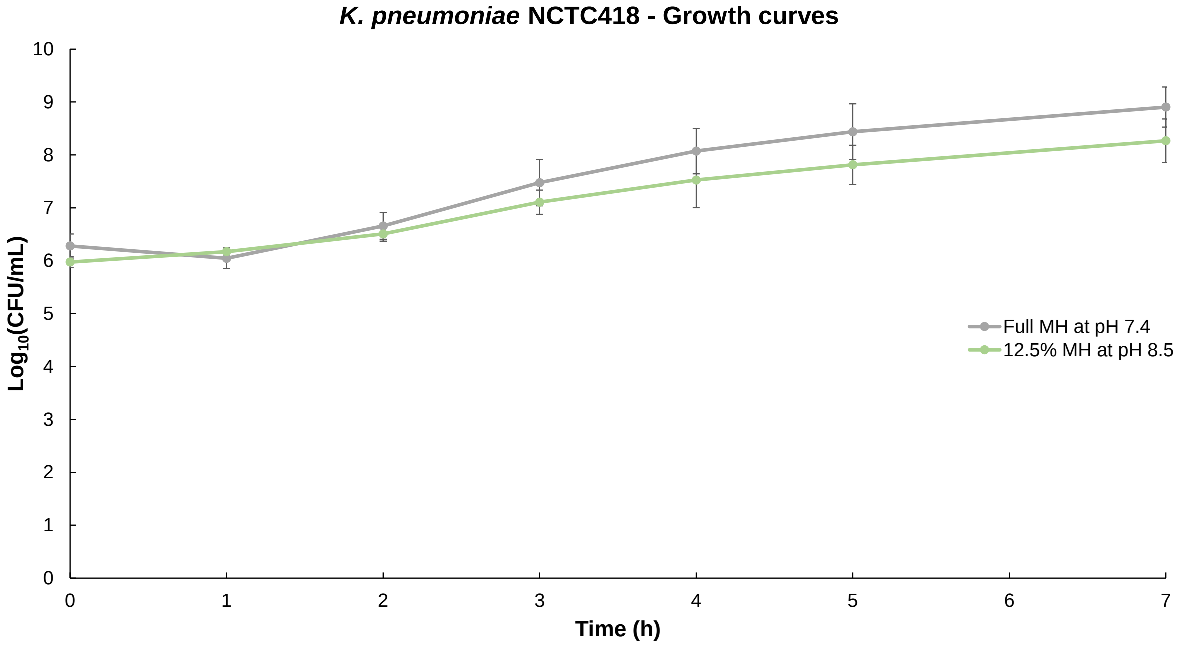
K. pneumoniae NCTC418 - Growth curves
Log10(CFU/mL)
0
1
2
3
4
5
6
7
8
9
10
Time (h)
0
1
2
3
4
5
6
7
Full MH at pH 7.4
12.5% MH at pH 8.5

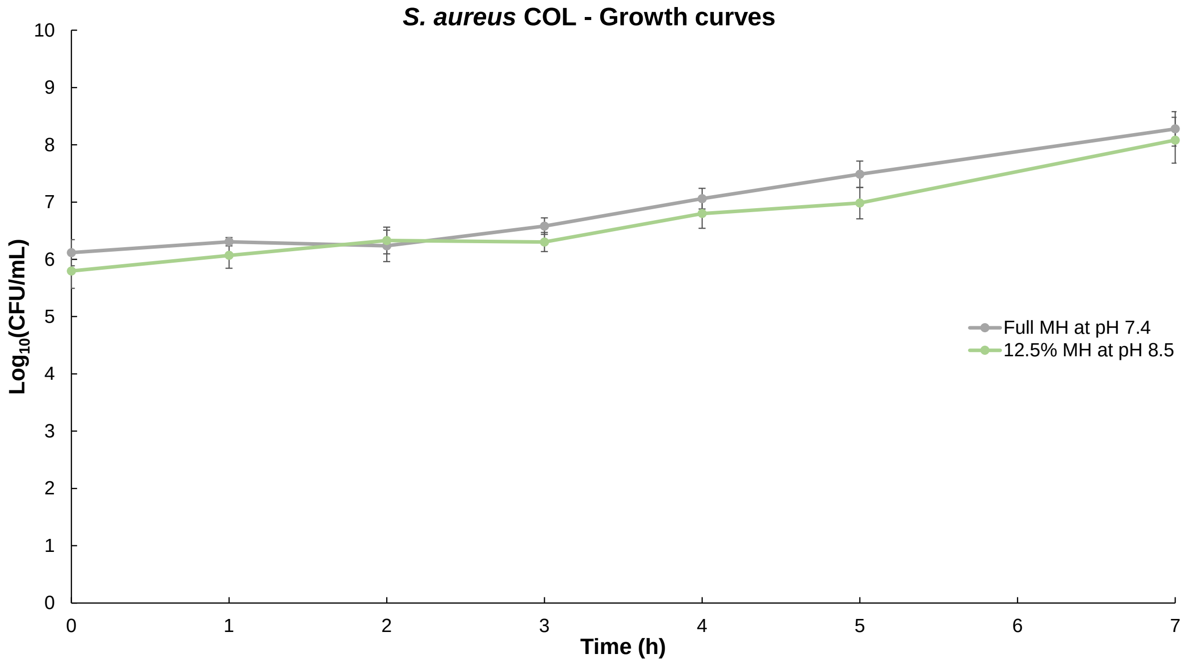


**Figure S1**. Bacteria growth curves, measured for the five bacteria strains.

**Time-Killing Kinetic Assay**

Single colonies of E. *coli* W3110 and *K. pneumoniae* NCTC418 were picked and grown overnight with shaking (180 rpm) in LB (Sigma-Aldrich, Buchs, Switzerland) medium 5 mL overnight at 37 °C. The overnight bacterial cultures were diluted to OD600 = 0.002 ($2 \times 10^6$ CFU/mL) in fresh MHB medium. Stock solutions of AMPs in sterilized milliQ water were prepared in 1 mg/mL and were diluted to two times more than required concentration in fresh MHB (Sigma- Aldrich, Buchs, Switzerland) medium at pH 7.4. 100 μL of prepared bacteria solution in MHB and 100 μL of samples in MHB were mixed in a 96-well microtiter plate (TPP, untreated, Corning Incorporated, Kennebunk, USA). Untreated bacteria at $1 \times 10^6$ CFU/mL were used as a growth control. 96-well microtiter plates were incubated in 37 °C with shaking (180 rpm). Surviving bacteria were quantified at 0, 0.5, 1, 2, 4 and 6 h by plating 10-fold dilutions of the sample in sterilized normal saline on LB agar plates. LB agar plates were incubated at 37 °C for 10 h, and the number of individual colonies was counted at each time point. The assay was performed in triplicate in a biosafety level 2 lab.

## Hemolysis assay

Minimum hemolytic concentration (MHC) was determined by serial dilution of a stock solution of 8 mg/mL of the peptide dendrimers in $H_2O$. 50 μL of the solution was diluted serially by ½ with 50 μL of PBS (pH 7.4) in 96-well plate (Corning-Costar or Nunc, polystyrene, untreated) and 50 μL of blood sample (see the blood preparation below) was added to the wells resulting in the final concentration of the compounds ranging from 1000 to 0.49 μg/mL. Human red blood cells (hRBC) were obtained from Interregionale Blutspende SRK AG, Bern. 1.5 mL of whole blood was centrifuged at 3000 rpm for 15 minutes. The plasma was discarded and the pellet was re-suspended in 5 mL of PBS. The washing was repeated three times and the remaining pellet was re-suspended in 10 mL of PBS at a final hRBC concentration of 5%. The hRBC suspension (50 μL) was added to each well and the plate was incubated at room

temperature for 4 hours. Minimal hemolytic concentration (MHC) end points were determined by visual inspection of the wells after the incubation period. Controls on each plate included a blank medium control (50 µL PBS + 50 µL of hRBC suspension) and a hemolytic activity control (mQ-deionized water 50 µL + 50 µL hRBC suspension).

## Ribosome Footprinting

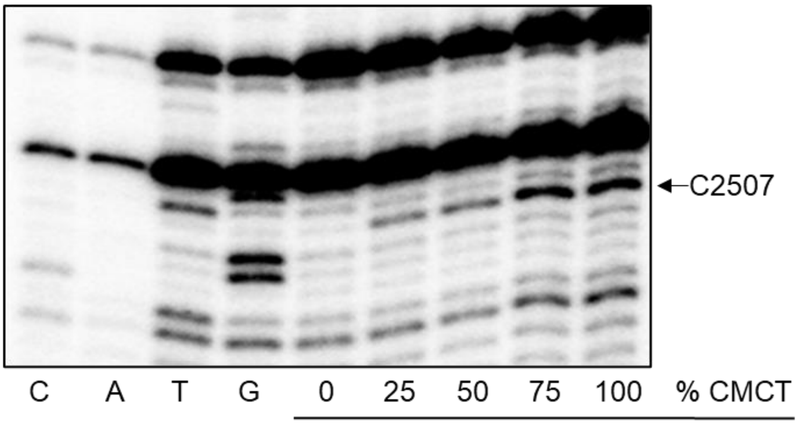


**Figure S2.** Gel electrophoresis to determine the optimal concentration of CMCT. A footprinting test experiment was conducted with concentration of CMCT ranging from 0 to 100%. We decided to use a concentration of 25% CMCT for our next experiments.

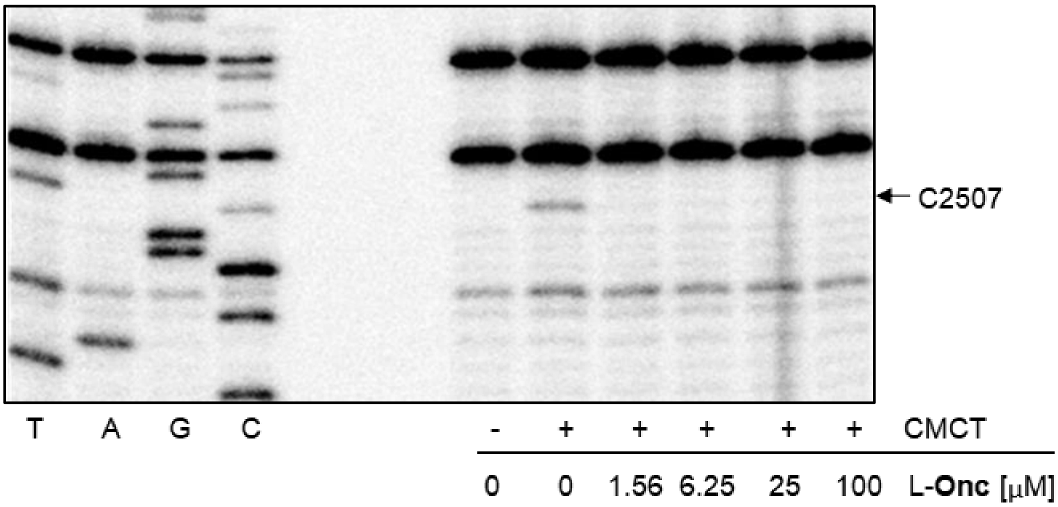


**Figure S3**. Gel electrophoresis for the determination of the optimal concentration of peptide. A footprinting test experiment was conducted with **L-Onc** concentrations ranging from 0 to 100 µM. Based on this result, we chose a peptide concentration of 1 µM for the following experiments.

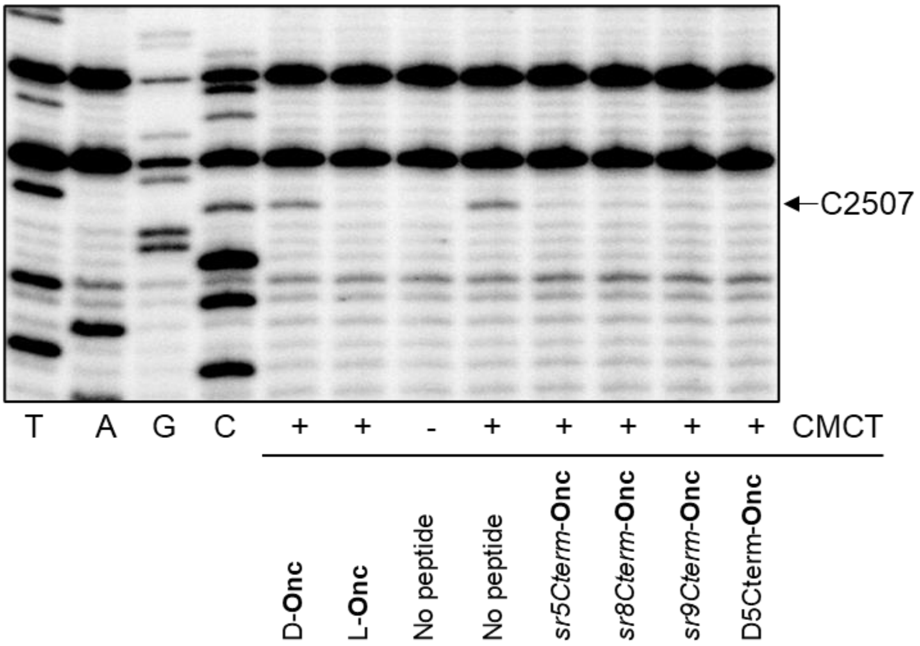


**Figure S4**. Sequencing gel of the ribosome footprinting experiment 1. The dark bands result from the U2506 CMCT modification in absence of peptide binding. CMCT+ modifies U2506 and produces a visible band at the position C2507. CMCT- does not modifies U2506. No band are observable with L-**Onc**, *sr5Cterm*-**Onc**, *sr8Cterm*-**Onc**, *sr9Cterm*-**Onc** and D5Cterm-**Onc** compared to background; revealing the interaction of the peptide with U2506. On the other hand, a band is visible with D-**Onc** resulting from the absence of peptide binding.

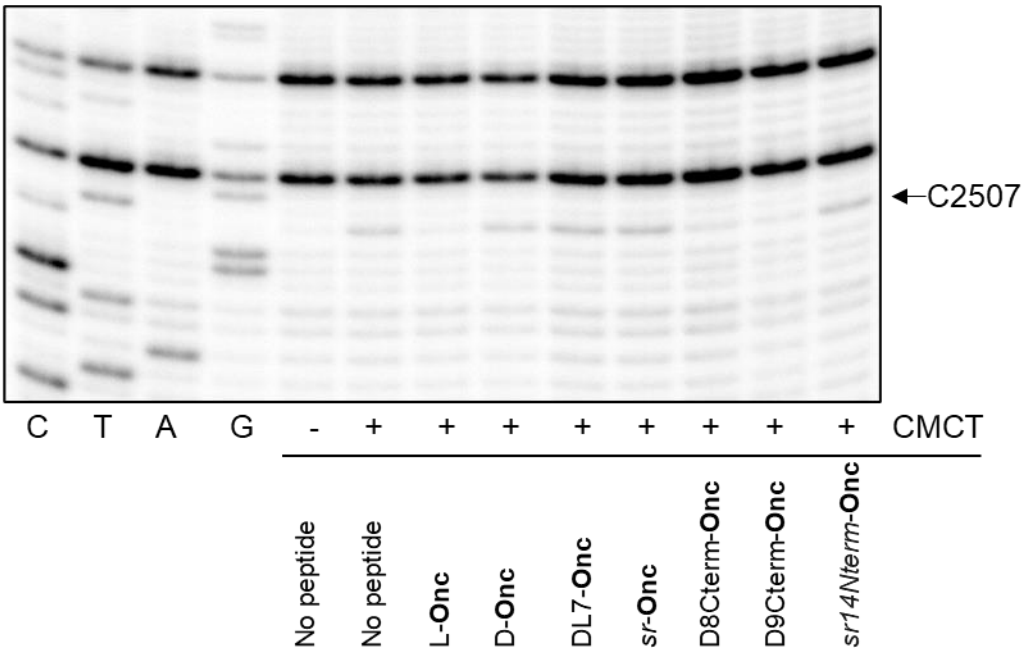


**Figure S5**. Sequencing gel of the ribosome footprinting experiment 2. The dark bands result from the U2506 CMCT modification in absence of peptide binding. CMCT+ modifies U2506 and produces a visible band at the position C2507. CMCT- does not modifies U2506. No band are observable with L-**Onc**, D8Cterm-**Onc** and D9Cterm-**Onc** compared to background; revealing the interaction of the peptide with U2506. On the other hand, a band is visible with D-**Onc,** DL7-**Onc,** *sr*-**Onc** and *sr14Nterm*-**Onc** resulting from the absence of peptide binding.

## Transmission electron microcopy

Exponential phase of *K. pneumoniae* NCTC418 and *E. coli* W3110 were washed with PBS and treated with azithromycin (20 µg/mL), **L-Onc** (40 µg/mL), ***sr5Cterm*-Onc** (20 µg/mL), ***sr9Cterm*-Onc** (40 µg/mL), and **D5Cterm-Onc** (40 µg/mL) in MH broth at 10x MIC with shaking. Each time, 1 ml of the bacteria were centrifuged after 60 min at 12’000 rpm for 3 min and fixed overnight with 2.5% glutaraldehyde (Agar Scientific, Stansted, Essex, UK) in 0.15M HEPES (Fluka, Buchs, Switzerland) with an osmolarity of 670 mOsm and adjusted to a pH of 7.35. The next day, bacteria were washed with 0.15 M HEPES three times for 5 min, postfixed with 1% OsO4 (SPI Supplies, West Chester, USA) in 0.1 M Na-cacodylate-buffer (Merck, Darmstadt, Germany) at 4°C for 1 h. Thereafter, bacteria cells were washed in 0.1 M Na-cacodylate-buffer three times for 5 min and dehydrated in 70, 80, and 96% ethanol (Alcosuisse, Switzerland) for 15 min each at room temperature. Subsequently, they were immersed in 100% ethanol (Merck, Darmstadt, Germany) three times for 10 min, in acetone (Merck, Darmstadt, Germany) two times for 10 min, and finally in acetone-Epon (1:1) overnight at room temperature. The next day, bacteria cells were embedded in Epon (Fluka, Buchs, Switzerland) and hardened at 60°C for 5 days.

Sections were produced with an ultramicrotome UC6 (Leica Microsystems, Vienna, Austria), first semithin sections (1um) for light microscopy which were stained with a solution of 0.5% toluidine blue O (Merck, Darmstadt, Germany) and then ultrathin sections (70-80nm) for electron microscopy. The sections, mounted on single slot copper grids, were stained with uranyl acetate and lead citrate with an ultrostainer (Leica Microsystems, Vienna, Austria).

Sections were then examined with a Tecnai Spirit transmission electron microscope equipped with two digital cameras (Olympus-SIS Veleta CCD Camera, FEI Eagle CCD Camera).

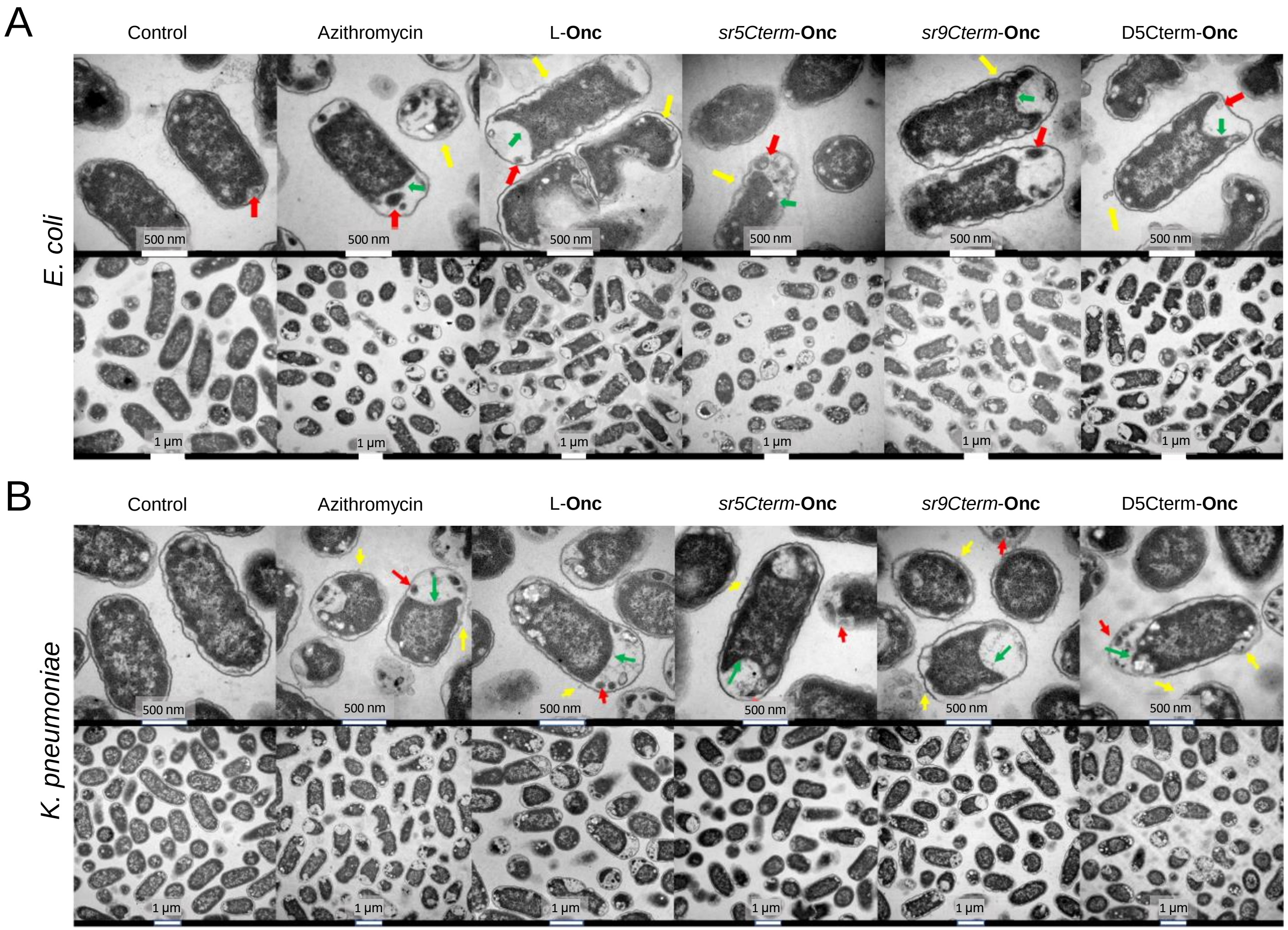


**Figure S6**. TEM images. **A.** Untreated *E. coli* or treated with 10x MIC of azithromycin (20 µg/mL), L-**Onc** (40 µg/mL), *sr5Cterm*-**Onc** (20 µg/mL), *sr9Cterm*-**Onc** (40µg/mL), and D5Cterm-**Onc** (40 µg/mL). Green arrows: inner membrane detached from the outer membrane; Red arrows: intracellular vesicles; yellow arrows: membrane perturbations. White scale bar: 500 nm or 1 µm. **B.** TEM images of untreated K. pneumoniae or treated with 10x MIC, with the same compounds and at the same concentration as in **A.**

## Serum stability

Human serum was diluted in 0.1 M filtered TRIS buffer pH 7.4 (50%, 1:1, v/v). Selected peptides were diluted in 0.1 M filtered TRIS buffer pH 7.4 to a concentration of 400 μM and 0.1 mg/mL 4- hydroxybenzoic acid was added as internal standard. Aliquots of peptide solution (300 μL) were added to aliquots of serum (300 μL) in sterile 1.5 mL Eppendorf tubes, to reach a peptide concentration of 200 μM and 25% (v/v) human serum during the assay. Samples, in triplicate, were incubated at 37 °C under gentle stirring (200 rpm). At different time points (0, 24, 48 and 72 hours), 100 μL of each sample was collected and quenched by precipitating serum proteins through the addition of (0.1 M) $ZnSO_4 \cdot 7\,H_2O$ / ACN (1:1) (0.1 M, 100 μL) and cooling in ice bath for 10 minutes. Protein precipitates were pelleted under centrifugation (5 min at 14’000 rpm) and the supernatants were analysed by LC-MS. Peaks corresponding to the internal standard and the undegraded peptides were integrated from the area under the curves (AUC) of UV traces, with the ratio peptide/standard at t = 0 h as 100%.

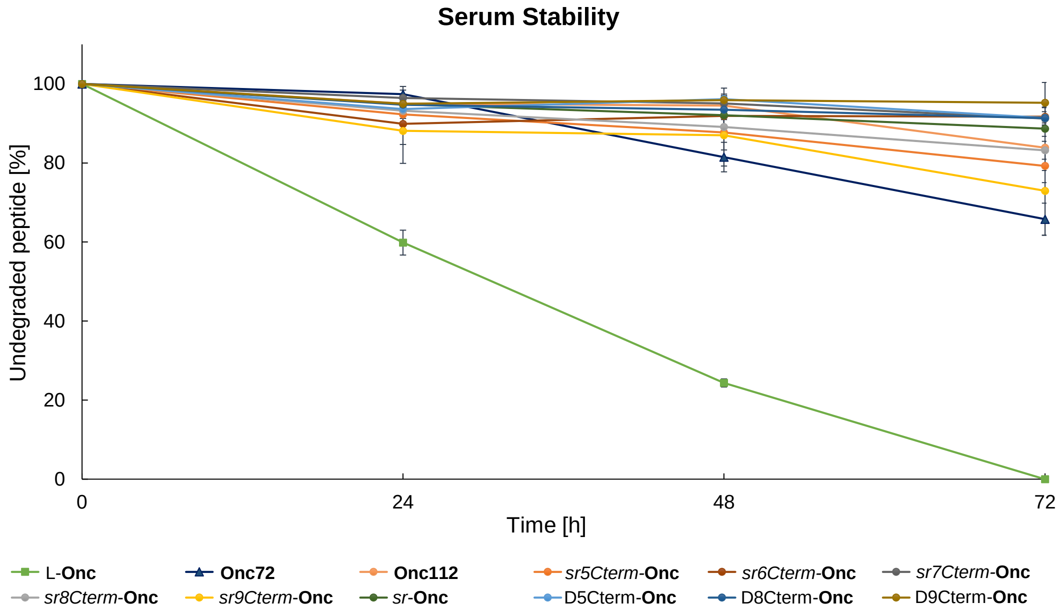


**Figure S7**. Serum stability assay of the respective compound (200 μM), incubated with human serum (25% in TRIS buffer, 0.1 M, pH 7.4) at 37 °C. Aliquots were taken at different time (0 / 24 / 48 and 72 h). Undegraded peptide values determined by RP-HPLC analysis using hydroxybenzoic acid as internal standard. Measured in triplicates and the data represent mean ± SD, n = 3.

## Vesicle Leakage Assay

*Vesicle preparation.* 5(6)-carboxyfluorescein (CF) was purchased from Sigma Aldrich. Egg Yolk Phosphatidylcholine (EYPC), Egg Yolk Phosphatidylglycerol (EYPG) and a Mini-Extruder were purchased from Avanti Polar Lipids. Egg PC or Egg PG thin lipid layers were prepared by evaporating a solution of 100 mg EYPC or EYPG in 4 mL MeOH/$CHCl_3$ (1:1) on a rotary evaporator at room temperature and then dried in vacuo overnight. The resulting film was then hydrated with 2 mL CF buffer (50 mM CF, 10 mM TRIS, 10 mM NaCl, pH 7.4) for 30 min, subjected to freeze-thaw cycles (7×) and extrusion (15×) through a polycarbonate membrane (pore size 100 nm). Extra vesicular components were removed by gel filtration (Sephadex G-50) with 10 mM TRIS, 107 mM NaCl, pH 7.4 buffer. Final conditions: ca. 2.5 mM EYPC or EYPG; inside: 50 mM CF, 10 mM TRIS, 10 mM NaCl, pH 7.4 buffer; outside: 10 mM TRIS, 107 mM NaCl, pH 7.4.

*Vesicle leakage assay*. EYPC or EYPG vesicle stock solutions (37.5 μL) were diluted to 3000 μL with a buffer (10 mM TRIS, 107 mM NaCl, pH 7.4) in a thermostated fluorescence cuvette (25 °C) and gently stirred (final lipid concentration ca. 10 μg/mL for both EYPG and EYPC). The desired volume of peptide (1 or 20 mg/mL stock in MilliQ water) was added at t = 45 s to obtain final peptide concentrations of 10 and 50 μg/mL, and CF efflux was monitored at $\lambda_{em}$ 517 nm ($\lambda_{ex}$ 492 nm) as a function of time. Finally, 30 μL of 1.2% Triton X-100 was added to the cuvette (0.012% final concentration) at time between 240 s and 280 s to reach the maximum intensity. Fluorescence intensities were then normalized to the maximal emission intensity using $I(t) = (I_t - I_0)/(I_\infty - I_0)$ where $I_0 = I_t$ at peptide addition and $I_\infty = I_t$ at saturation of lysis.

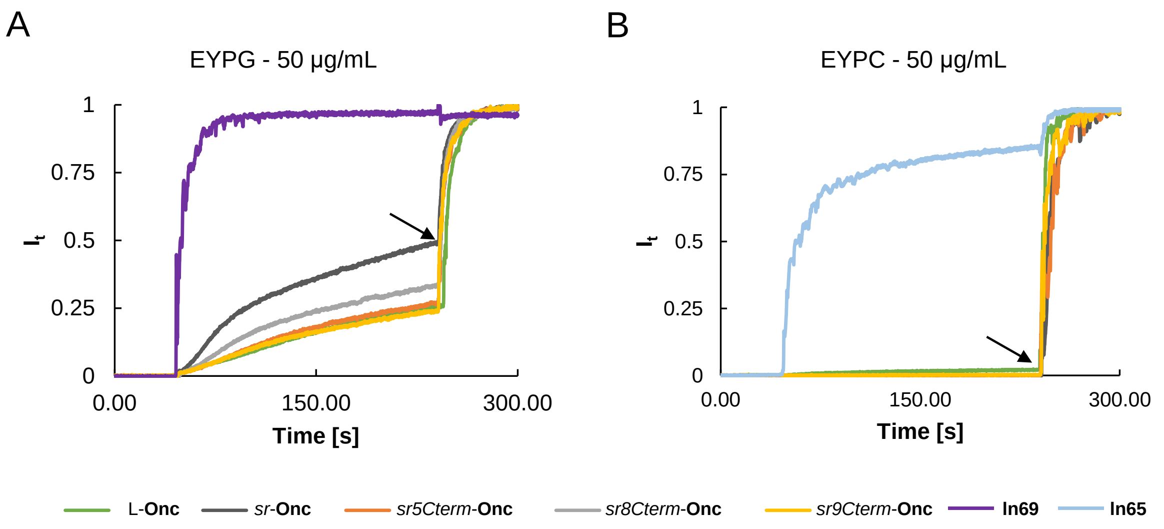


**Figure S8**. Fluorescein leakage assay from egg yolk phosphatidyl glycerol (EYPG, **A**) and egg yolk phosphatidyl choline (EYPC, **B**) vesicles. The indicated compound at 50 µg/mL (10 µg/mL for positive controls **ln69** and **ln65**) was added to a suspension of fluorescein loaded EYPG or EYPC vesicles suspended in buffer (10 mM Tris, 107 mM NaCl, pH adjusted to 7.4). The black arrows indicate the time of addition of Triton X-100 at 240s.

## Circular Dichroism Spectroscopy

Circular dichroism (CD) experiments were measured on a Jasco J-715 Spectropolarimeter. All the experiments were performed using Hellma Suprasil 110-QS 0.1 cm cuvettes. For each peptide, the measurements were performed in phosphate buffer (PB, pH = 7.4, 7 mM), 20% trifluoroethanol (TFE) in PB (pH = 7.4, 7 mM) and in 5 mM dodecylphosphocholine (DPC) in PB. The buffer was degassed for 30 min under high vacuum before each set of experiments. The concentration of the peptides was 0.1 mg/mL and each sample was measured in one accumulation. The scan rate was 20 nm/min, pitch 0.5 nm, response 16 sec and bandwidth 1.0 nm. The nitrogen flow was kept > 8.5 L/min. After each measurement, the cuvettes were washed successively with MilliQ water and PB buffer. The different baselines were recorded under the same conditions and subtracted manually.

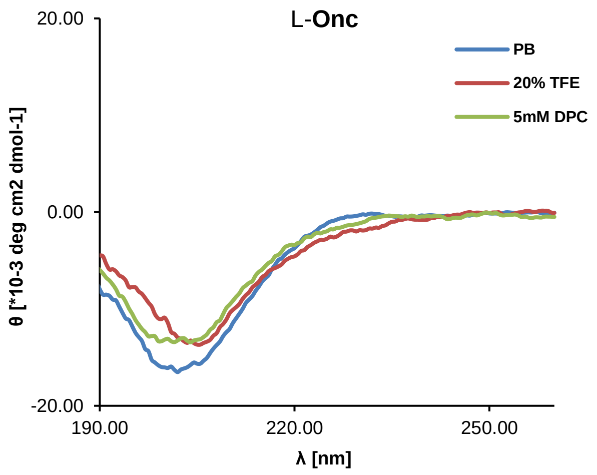
L-**Onc**
PB
20% TFE
5mM DPC
20.00
0.00
-20.00
190.00
220.00
250.00
θ [*10-3 deg cm2 dmol-1]
λ [nm]

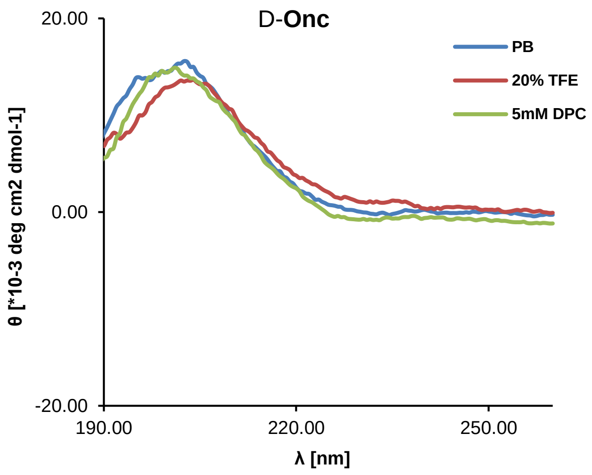
D-**Onc**
PB
20% TFE
5mM DPC
20.00
0.00
-20.00
190.00
220.00
250.00
θ [*10-3 deg cm2 dmol-1]
λ [nm]

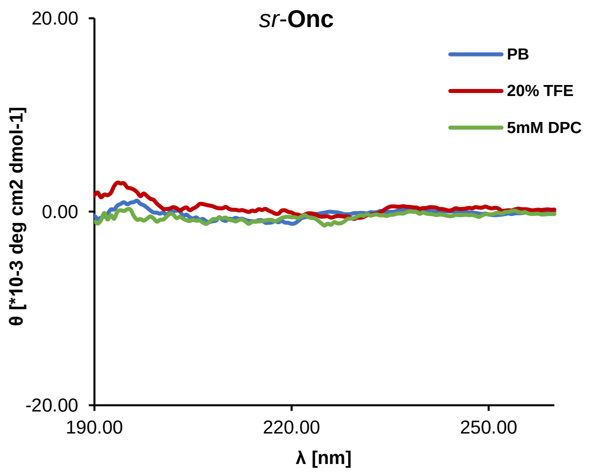
*sr*-**Onc**
PB
20% TFE
5mM DPC
20.00
0.00
-20.00
190.00
220.00
250.00
θ [*10-3 deg cm2 dmol-1]
λ [nm]

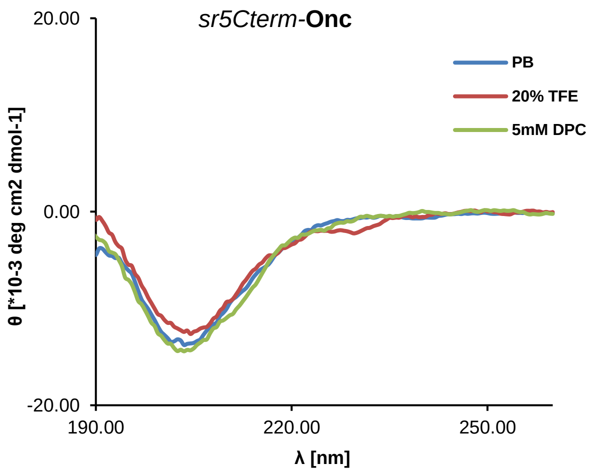
*sr5Cterm*-**Onc**
PB
20% TFE
5mM DPC
20.00
0.00
-20.00
190.00
220.00
250.00
θ [*10-3 deg cm2 dmol-1]
λ [nm]

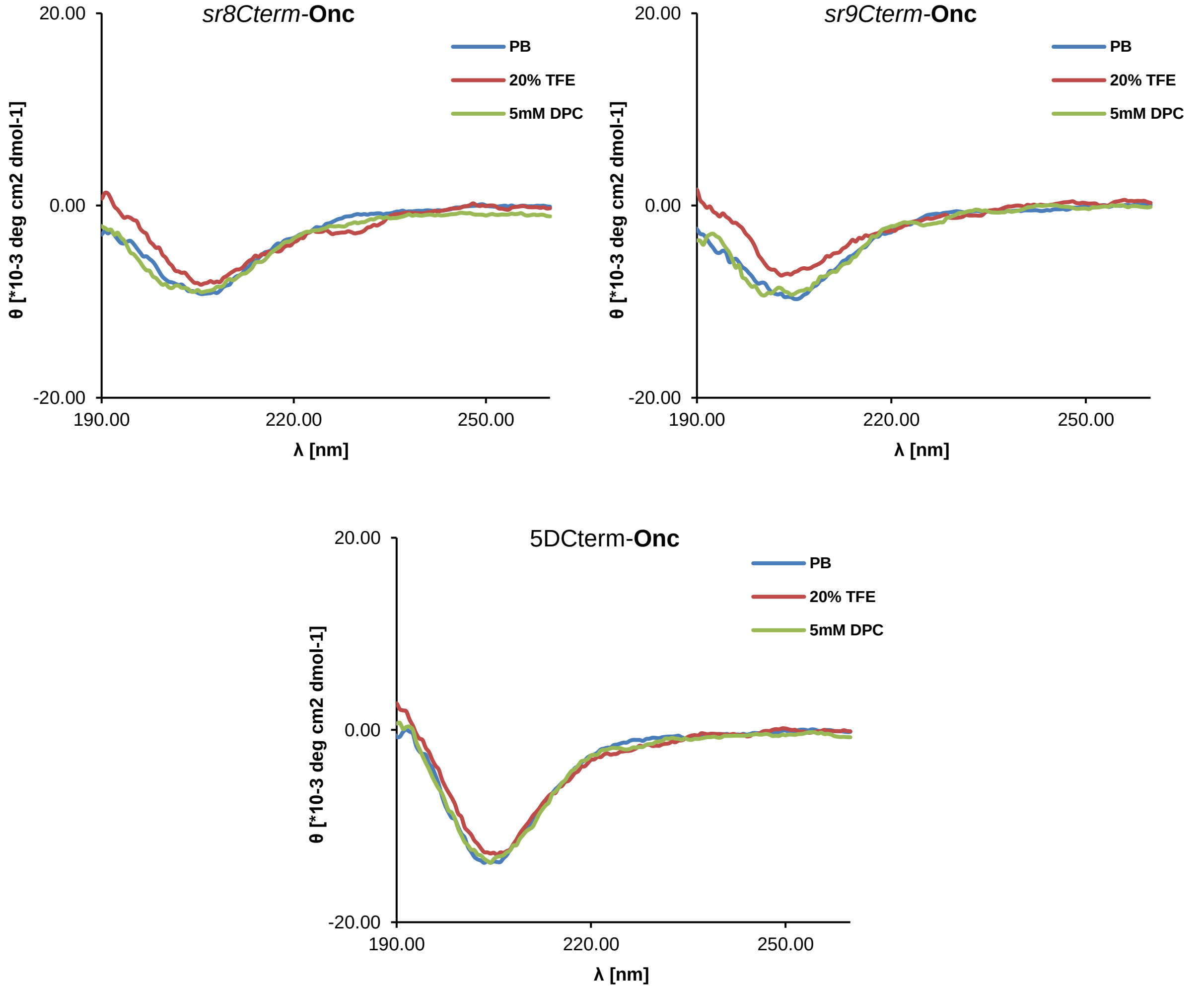


**Figure S9**. Circular dichroism spectra of the peptides, at 100 µg/mL, measured in three different conditions: phosphate buffer (10 µM, pH 7.4, blue line) / with additional trifluoroethanol (20%, red line) / with additional dodecylphosphocholine (5 mM, green line).

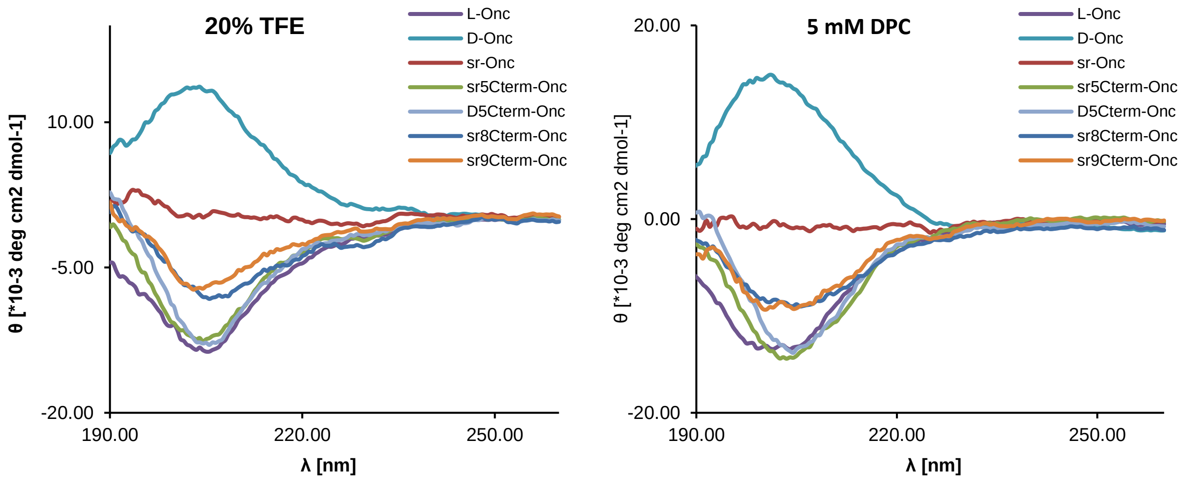


**Figure S10**. Comparisons of the circular dichroism, measured in 20% trifluoroethanol and 5 mM dodecylphosphocholine respectively.

## DnaK experiments

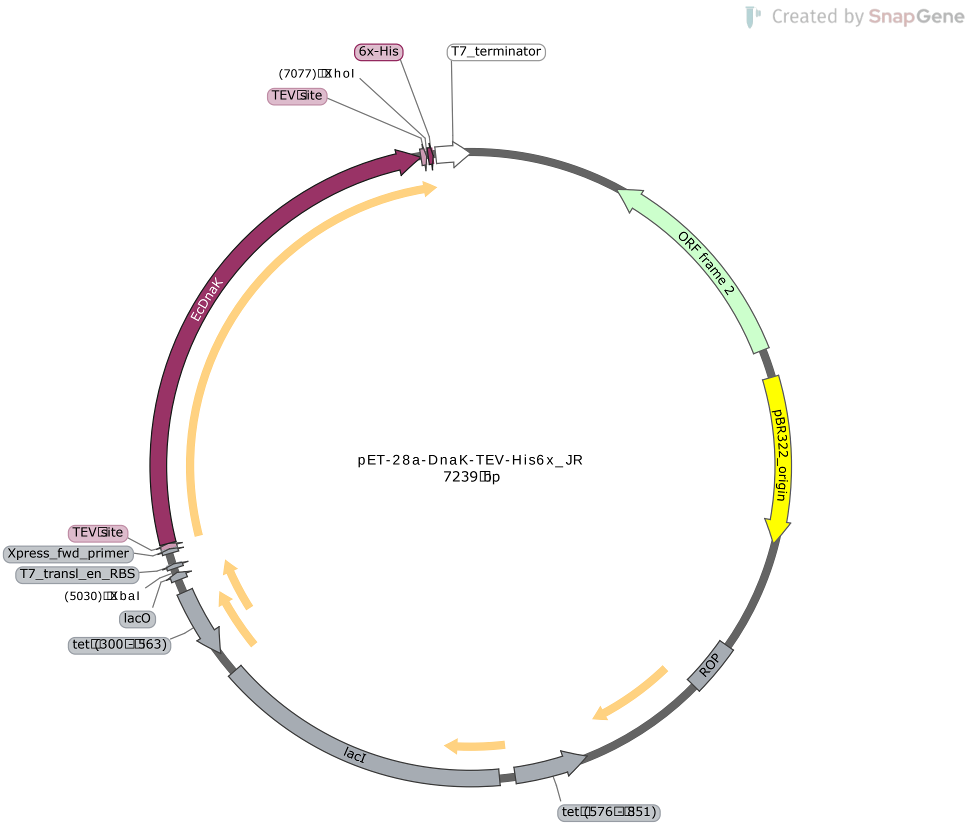


**Figure S11**. Map of pET-28a T7 expression vector with DnaK insert. Translated sequence: MGKIIGIDLGTTNSCVAIMDGTTPRVLENAEGDRTTPSIIAYTQDGETLVGQPAKRQAVTNPQ NTLFAIKRLIGRRFQDEEVQRDVSIMPFKIIAADNGDAWVEVKGQKMAPPQISAEVLKKMKK TAEDYLGEPVTEAVITVPAYFNDAQRQATKDAGRIAGLEVKRIINEPTAAALAYGLDKGTGN RTIAVYDLGGGTFDISIIEIDEVDGEKTFEVLATNGDTHLGGEDFDSRLINYLVEEFKKDQGIDL RNDPLAMQRLKEAAEKAKIELSSAQQTDVNLPYITADATGPKHMNIKVTRAKLESLVEDLVN RSIEPLKVALQDAGLSVSDIDDVILVGGQTRMPMVQKKVAEFFGKEPRKDVNPDEAVAIGAA VQGGVLTGDVKDVLLLDVTPLSLGIETMGGVMTTLIAKNTTIPTKHSQVFSTAEDNQSAVTIH VLQGERKRAADNKSLGQFNLDGINPAPRGMPQIEVTFDIDADGILHVSAKDKNSGKEQKITIK ASSGLNEDEIQKMVRDAEANAEADRKFEELVQTRNQGDHLLHSTRKQVEEAGDKLPADDKT AIESALTALETALKGEDKAAIEAKMQELAQVSQKLMEIAQQQHAQQQTAGADASANNAKDD DVVDAEFEEVKDKKENLYFQSLEHHHHHH.

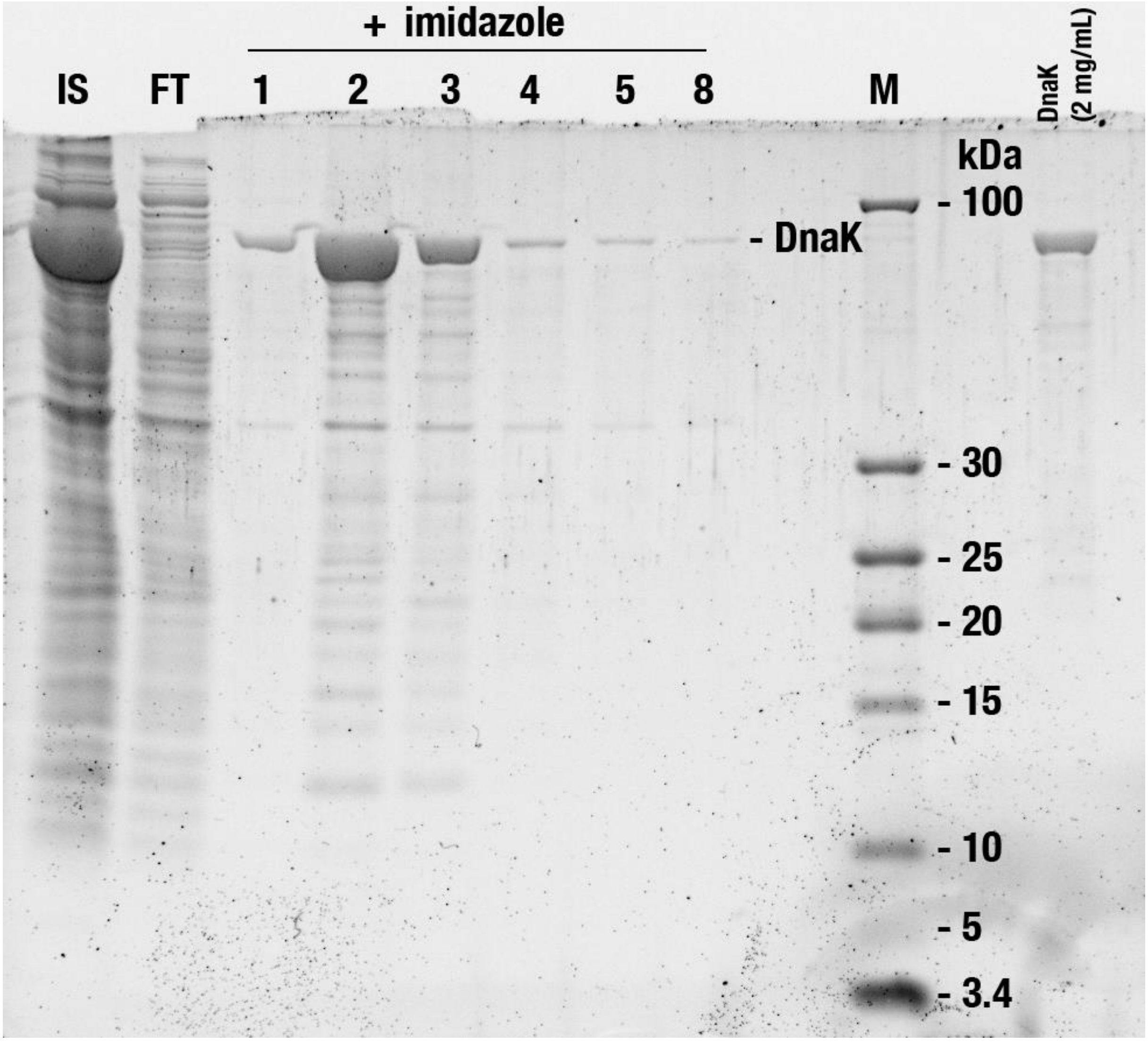


**Figure S12**. 10% 1:19 bis-/acrylamide Tris-tricine SDS-PAGE of the his-tagged DnaK purification. (IS – input sample/crude *E. coli* lysate, FT – flowthrough/unbound protein) After combining the eluted fractions containing DnaK and adjusting the loading to 2 mg/mL, purity of >90% is obtained.

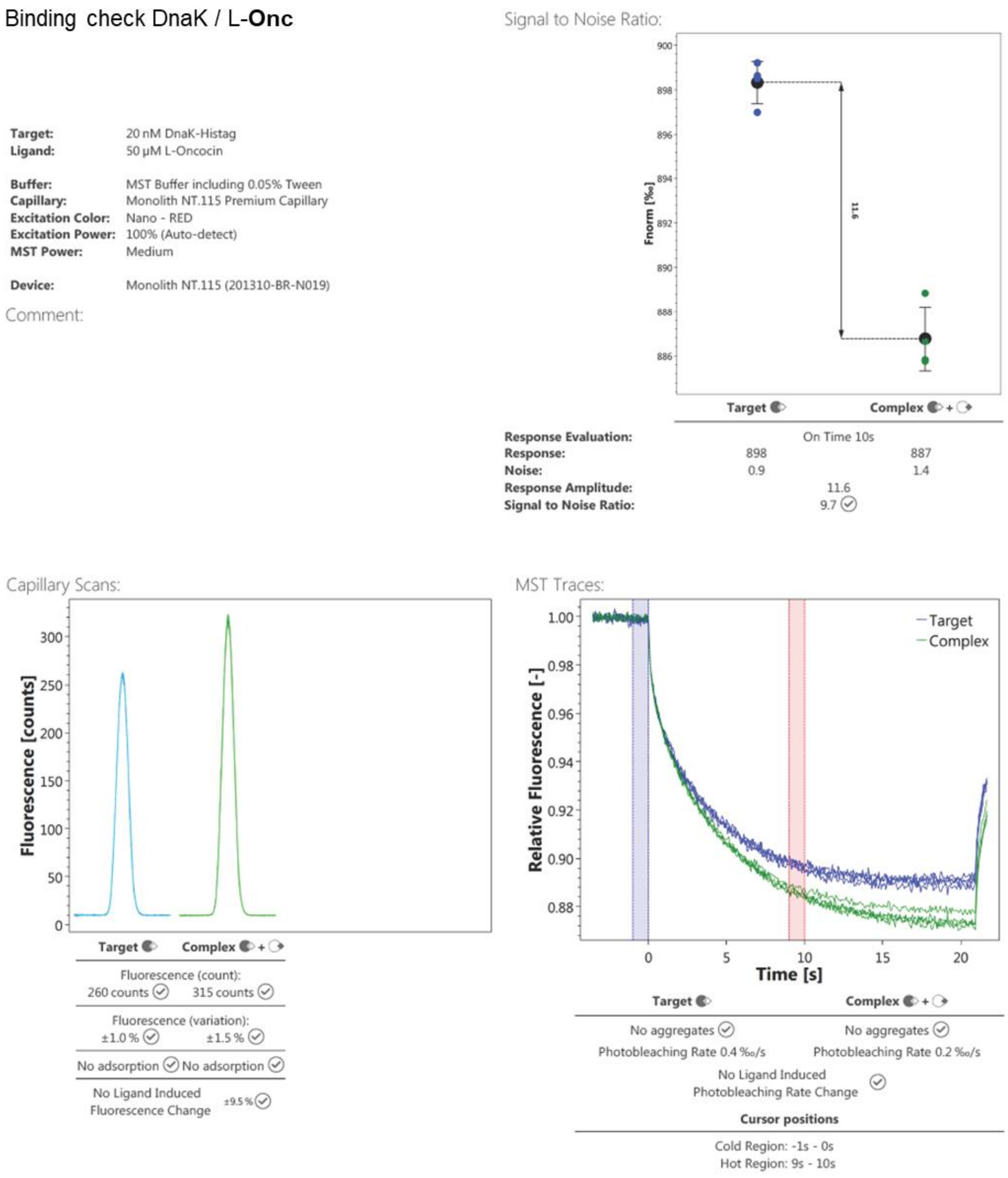


**Figure S13**. Microscale thermophoresis, binding check experiment between his-tag labelled DnaK and L-**Onc**. Signal to noise ratio showed a difference of fluorescence signal between his-tag labelled DnaK alone (blue trace) and the complex his-tag labelled DnaK-L-**Onc** (green trace). Capillary scans showed the fluorescence intensity of each sample and demonstrated that no adsorption happened in the capillary. MST traces showed the thermophoresis curves for his-tag labelled DnaK alone and the complex his-tag labelled DnaK-L-**Onc**. This experiment was positive for binding.

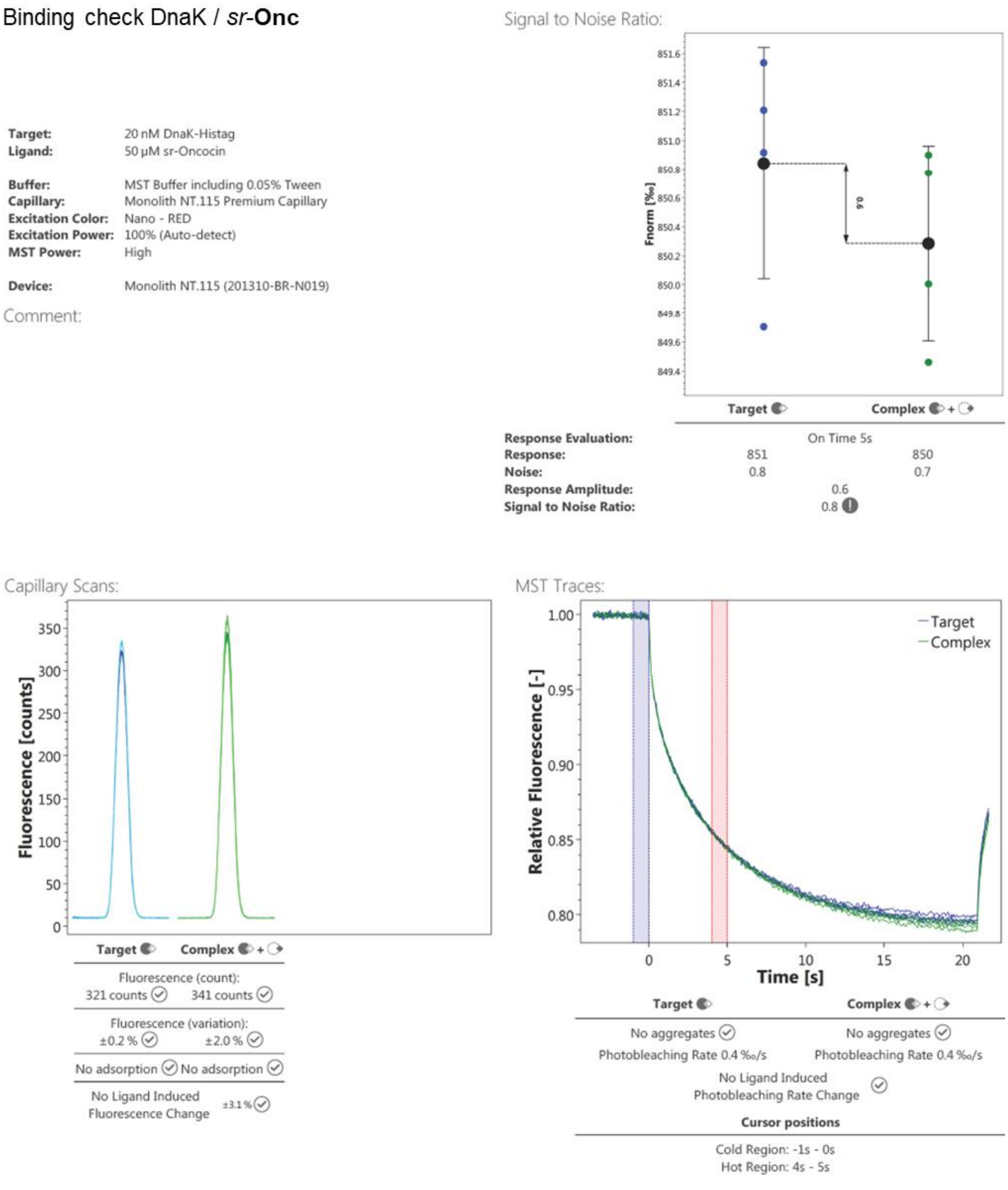


**Figure S14**. Microscale thermophoresis, binding check experiment between his-tag labelled DnaK and *sr*-**Onc**. Signal to noise ratio showed the difference of fluorescent signal between his-tag labelled DnaK alone (blue trace) and the complex his-tag labelled DnaK-*sr*-**Onc** (green trace). Capillary scans showed the fluorescence intensity of each sample and demonstrated that no adsorption happened in the capillary. MST traces showed the thermophoresis curves for his-tag labelled DnaK alone and the complex his-tag labelled DnaK-*sr*-**Onc**. This experiment was negative for binding.

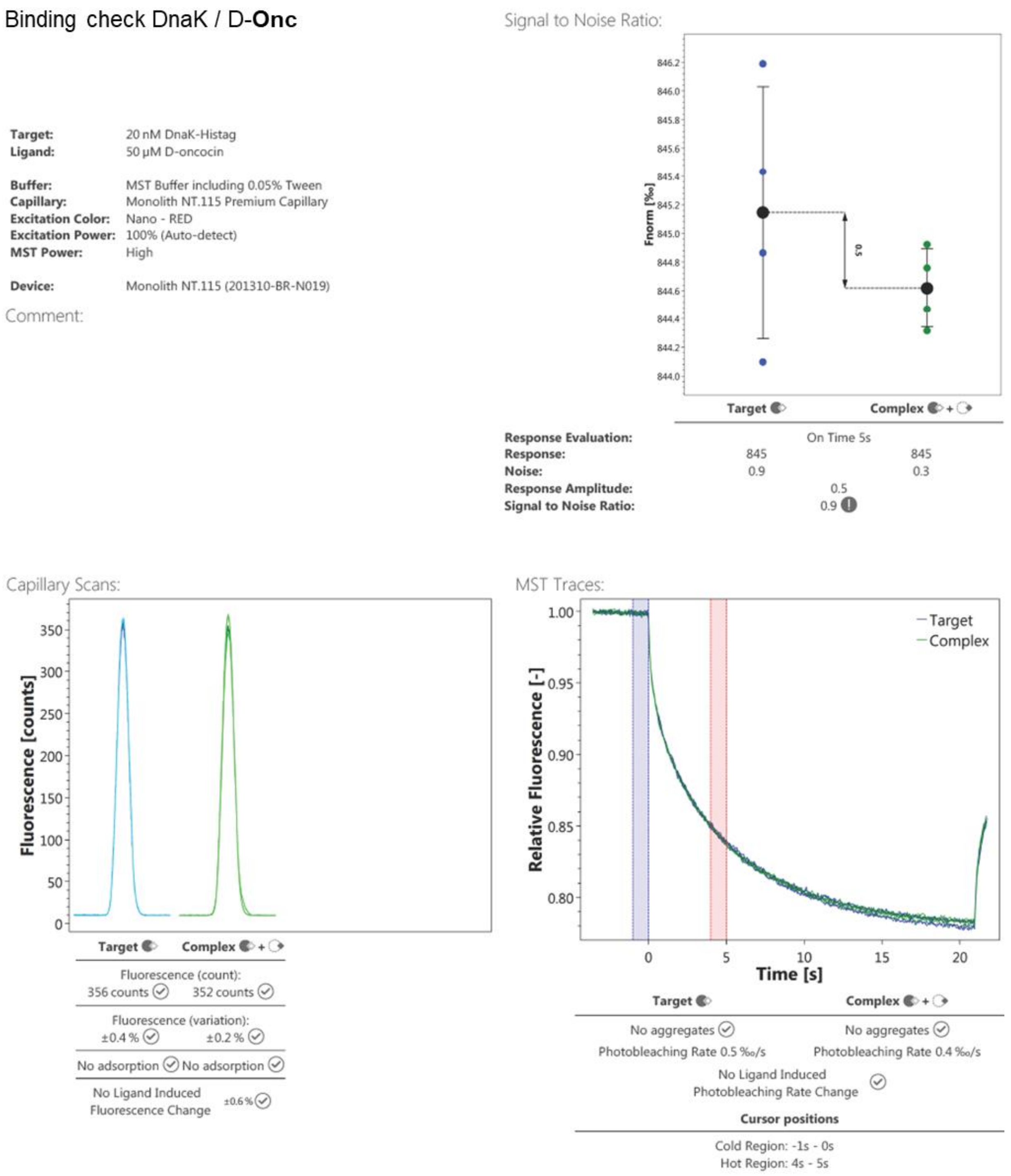


**Figure S15**. Microscale thermophoresis, binding check experiment between his-tag labelled DnaK and D-**Onc**. Signal to noise ratio showed the difference of fluorescent signal between his-tag labelled DnaK alone (blue trace) and the complex his-tag labelled DnaK-D-**Onc** (green trace). Capillary scans showed the fluorescence intensity of each sample and demonstrated that no adsorption happened in the capillary. MST traces showed the thermophoresis curves for his-tag labelled DnaK alone and the complex his-tag labelled DnaK-D-**Onc**. This experiment was negative for binding.

## Binding affinity DnaK / L-**Onc**

| | |
|---|---|
| **Target:** | 20 nM DnaK-Histag |
| **Ligand:** | 100 µM L-Oncocin |
| **Buffer:** | MST Buffer including 0.05% Tween |
| **Capillary:** | Monolith NT.115 Premium Capillary |
| **Excitation Color:** | Nano - RED |
| **Excitation Power:** | 100% (Auto-detect) |
| **MST Power:** | Medium |
| **Device:** | Monolith NT.115 (201310-BR-N019) |

Comment:

### Dose Response

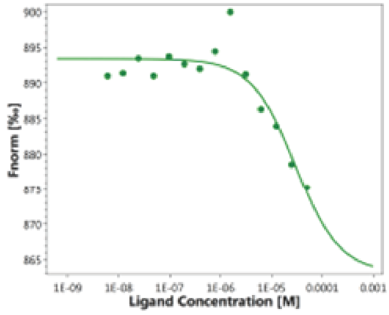


**Response Evaluation:** On Time 5s

**Kd model**

| | |
|---|---|
| **Unbound** | 893.4 |
| **Bound** | 863.2 |
| **Kd** | 29.8 µM |
| **TargetConc** | 20 nM |

**Response Amplitude:** 30.2
**Noise:** 2.6
**Signal to Noise Ratio:** 11.4

### Capillary Scans

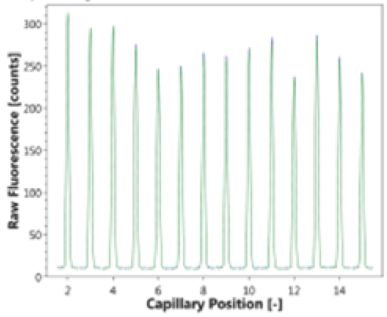


**Initial Fluorescence:**
**Average:** 267 counts
**Variation:** ±17.5 %

No adsorption

No Ligand Induced Fluorescence Change

### MST Traces

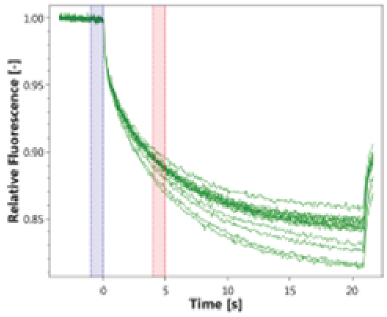


**Cursor positions:**
**Cold Region:** -1s - 0s
**Hot Region:** 4s - 5s

No Aggregation

No Ligand Induced Photobleaching Rate Change

**Figure S16**. Microscale thermophoresis, binding affinity experiment between his-tag labelled DnaK and L-**Onc**. The dose response curve showed a binding affinity of 29.8 µM. Capillary scans showed the fluorescence intensity of each sample and demonstrated that no adsorption happened in the capillary. MST traces showed the thermophoresis curves for each sample.
